# Synthesis and biochemical evaluation of cephalosporin analogues equipped with chemical tethers†

**DOI:** 10.1039/d0ra04893c

**Published:** 2020-10-02

**Authors:** Lisa M. Miller, Reyme Herman, Ivan Gyulev, Thomas F. Krauss, Gavin H. Thomas, Anne-Kathrin Duhme-Klair

**Affiliations:** Department of Chemistry, University of York Heslington York YO10 5DD UK lisa.miller@york.ac.uk; Department of Biology, University of York Heslington York YO10 5DD UK; Department of Physics, University of York Heslington York YO10 5DD UK

## Abstract

Molecular probes typically require structural modifications to allow for the immobilisation or bioconjugation with a desired substrate but the effects of these changes are often not evaluated. Here, we set out to determine the effects of attaching functional handles to a first-generation cephalosporin. A series of cephalexin derivatives was prepared, equipped with chemical tethers suitable for the site-selective conjugation of antibiotics to functionalised surfaces. The tethers were positioned remotely from the β-lactam ring to ensure minimal effect to the antibiotic's pharmacophore. Herein, the activity of the modified antibiotics was evaluated for binding to the therapeutic target, the penicillin binding proteins, and shown to maintain binding interactions. In addition, the deactivation of the modified drugs by four β-lactamases (TEM-1, CTX-M-15, AmpC, NDM-1) was investigated and the effect of the tethers on the catalytic efficiencies determined. CTX-M-15 was found to favour hydrolysis of the parent antibiotic without a tether, whereas AmpC and NDM-1 were found to favour the modified analogues. Furthermore, the antimicrobial activity of the derivatives was evaluated to investigate the effect of the structural modifications on the antimicrobial activity of the parent drug, cephalexin.

## Abstract 2

Tethered β-lactam antibiotics provide insights into designing chemical tools to target specific β-lactamases.

## Graphical abstract

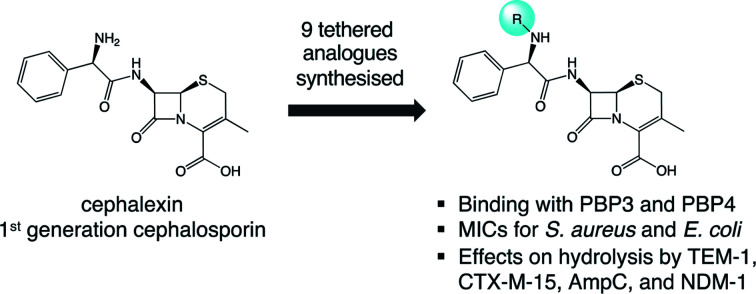

## Introduction

The controlled functionalisation of surfaces is imperative for the preparation of functional materials. This a key step in the preparation of many (bio)sensors, for example, which are designed for selective and sensitive detection of analytes. The immobilisation or bioconjugation of a molecular probe often requires structural modification to introduce a functional handle, able to react with a desired substrate. A common approach is to attach a linker to the probe, such as a bifunctional polyethylene glycol (PEG), with orthogonal functional groups that allows for controlled reaction with the probe and with the desired substrate.^1^ However, the effect of such modifications on the function of a probe are often not evaluated, even though binding interactions are likely to be affected. Consequently, opportunities to gain insights into the structure–activity relationships (SAR) are missed.

With the difficulties faced in the development of novel antibiotics and the increasing challenges of fighting against antibiotic resistance,^2,3^ investigations into the SAR of antibiotic analogues could reveal valuable information. The β-lactam antibiotics are widely used and are typically considered to be one of the safest classes of antibiotics.^4^ Since their discovery, there has been extensive research carried out into the derivatisation of the β-lactam scaffold, resulting in the successful development of numerous antibiotics.^5,6^

**Fig. 1:**
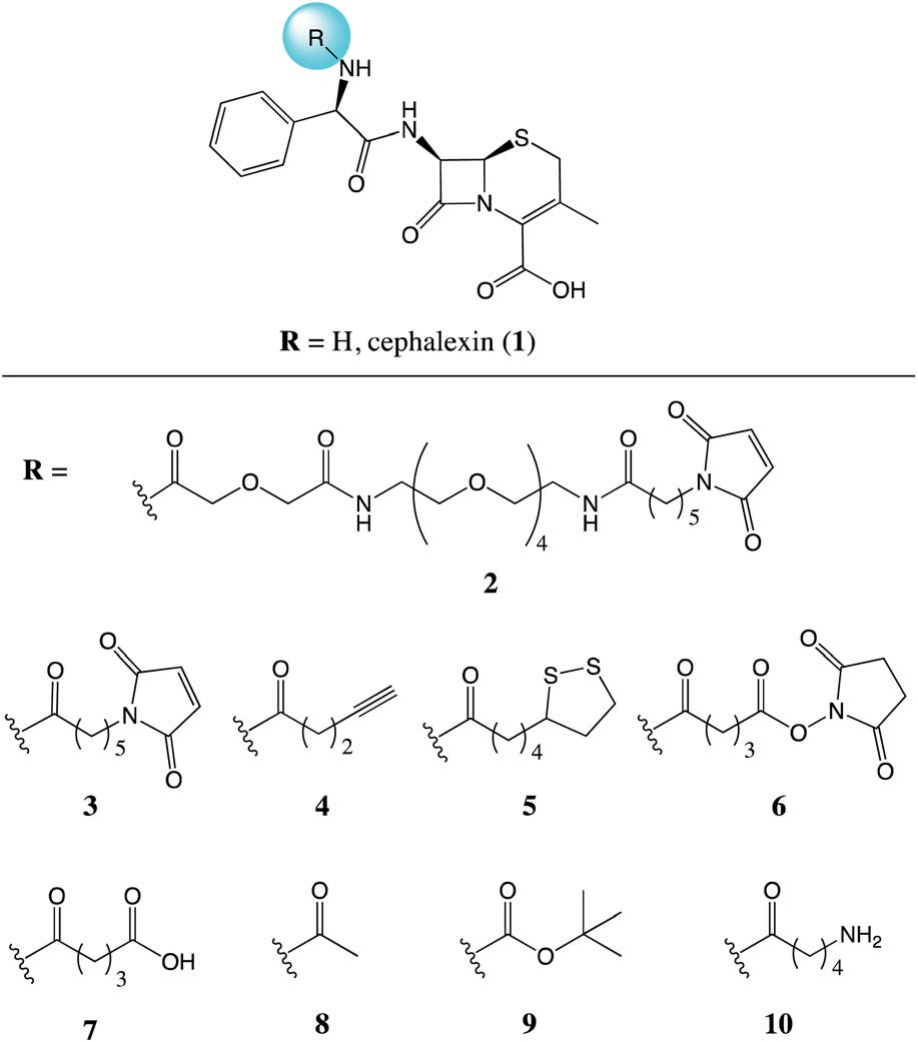
Chemical structures of the parent antibiotic cephalexin (1) and the nine analogues (2–10) evaluated for the effects of addition of a chemical tether.

Recently, we demonstrated the ability of surface bound β-lactam drugs to be recognised by the therapeutic target proteins as well as enzymes produced by resistant bacteria.^7^ In order to attach the antibiotic molecule to the surface, an analogue of cephalexin (1) was prepared featuring a maleimide group attached *via* a PEG linker (2), Fig. 1. Studies of the surface-bound antibiotic demonstrated that β-lactamases and a penicillin binding protein (PBP) were able to recognise and bind the immobilised drugs. Here, we set out to investigate the effect of the addition of a chemical tether on the properties of the parent compound, thus contributing new SAR information.

## Results and discussion

### Chemistry

#### Compound design

The modifications to 1 described herein were performed *via* the amine of the molecule. This position was chosen to minimise the effect of the tether on the pharmacophore, the β-lactam ring. The amine motif provided a functional handle for the addition of the desired chemical tether through amidation reactions, allowing access to a selection of compounds with reactive pendant groups. In addition to the previously reported compounds 2 and 3,^7^ a further seven analogues were synthesised, Fig. 1. Compounds 2–6 were designed to include commonly employed functional groups that are used in biocompatible reactions with surfaces and other substrates of interest.

Compounds 2 and 3 both feature a maleimide; this motif is a prevalent functional handle used for selective reaction through conjugation with thiol groups.^8^ Another commonly used bio-compatible reaction is the copper-catalysed click reaction in which an alkyne, such as in compound 4, reacts with an azide to form a triazole.^9^ For the direct attachment to gold surfaces disulfides, such as the lipoic acid tether of compound 5, are ubiquitous.^10^ Whereas, for the attachment to a protein, or another source of amine functional groups, *N*-hydroxysuccinimide (NHS) esters are common.^11^ Compounds with this activated ester, as in compound 6, react readily with lysine residues and other amines, providing attachment through the formation of an amide bond.

NHS-esters, such as compound 6, are known to have a short half-life in aqueous media;^11^ thus, the hydrolysis product with the carboxylic acid was prepared for comparison, compound 7. Three further control compounds were synthesised: one to test the effect of a small modification with the acetyl group of compound 8, whereas the *tert*-butyloxycarbonyl (Boc) group of compound 9 was designed to test the effect of a large sterically demanding group in this position. Lastly, compound 10 was included featuring a small aliphatic tether with a terminal amine group. By attaching the tethers *via* an amide linker in compounds 2–9, the amine of the parent antibiotic is lost. Therefore, compound 10 was included to determine the effects of introducing an amide and a short flexible tether, while maintaining an amine group.

#### Compound synthesis

**Scheme 1:**
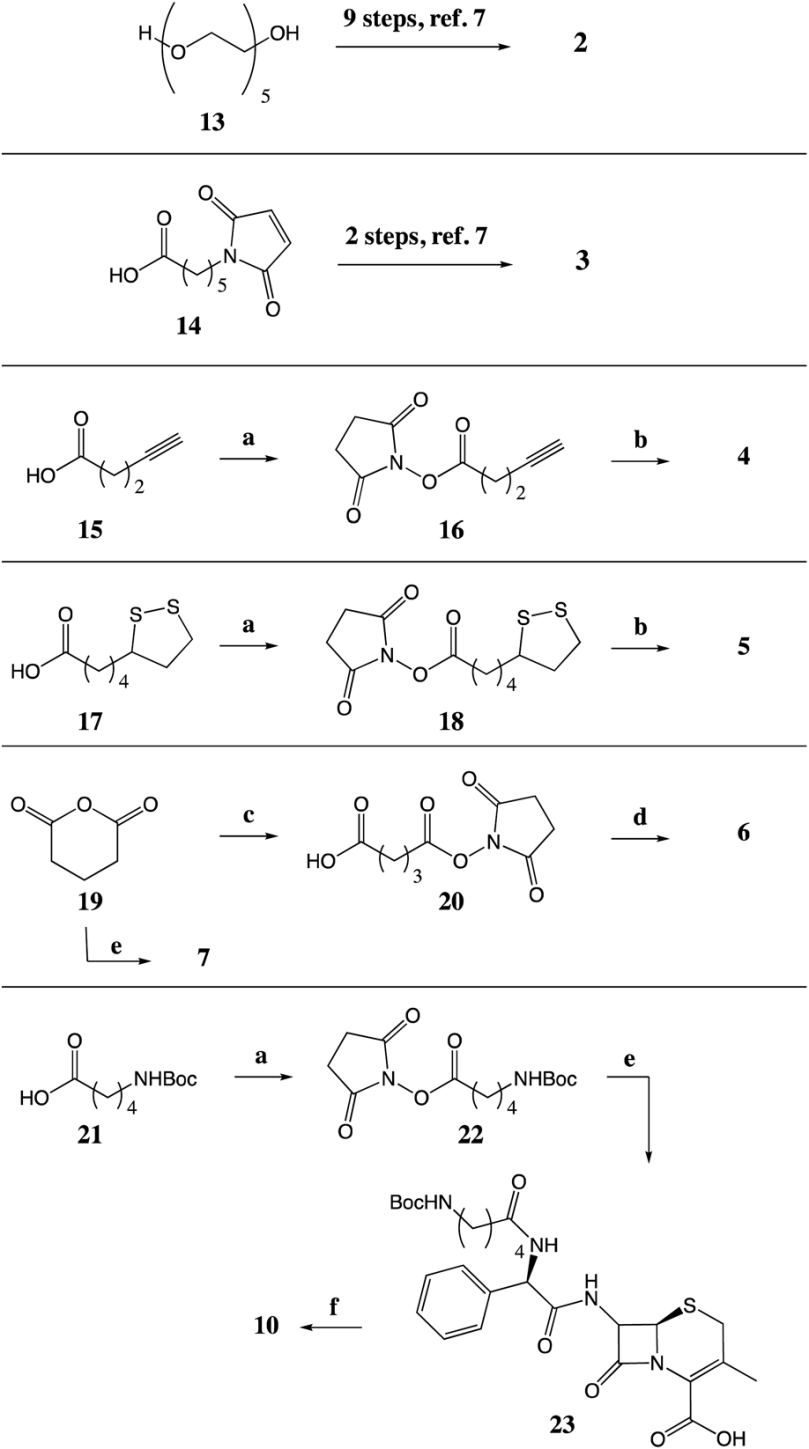
Synthesis of tethered analogues. (a) NHS, DCC, DCM; (b) 1, DIPEA, MeCN; (c) NHS, DMAP, THF; (d) (i) (ClCO)_2_, DMF, DCM, (ii) 1, anhydrous pyridine, MeCN; (e) 1, Et_3_N, MeCN; (f) TFA, TES, DCM.

The two maleimide analogues (2 and 3) were prepared as previously reported.^7^ All analogues were prepared by addition of the tethers to the parent antibiotic, as described in Scheme 1. Compounds 4 and 5 were prepared from reaction of 1 with the NHS-esters of the corresponding tether, intermediates 16 and 18. In the preparation of compound 6, the previously reported acid (20)^12^ was used to allow for the desired amidation *via* the acid chloride, while maintaining the NHS-ester. Compounds 7, 8, and 9 were prepared by reaction with the required anhydride reagent: glutaric anhydride, acetic anhydride and Boc anhydride, respectively. Finally, compound 10 was prepared in three steps from the *N*-Boc protected 5-aminovaleric acid 21, Scheme 1. All compounds were characterised by ^1^H, ^13^C NMR, IR spectroscopy, and HRMS. Purity of compounds for *in vitro* and *in vivo* testing was determined using HPLC or QNMR.

### Biological evaluation

#### PBP binding assay

**Fig. 2:**
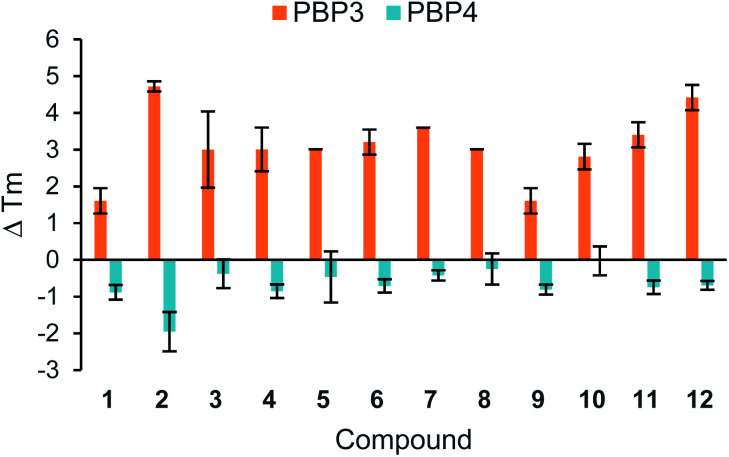
Thermal shift assay of compounds 1–12 with PBP3 and PBP4. Melting temperatures (*T*_m_) were calculated from the average of three measurements, with ±s.d. error. Δ*T*_m_ was calculated by subtracting the *T*_m_ of the PBP. The assay protocol is provided in the Experimental section.

A thermal shift assay was used to investigate the binding of the cephalexin analogues to the therapeutic target, PBP, *in vitro*. This assay provides a fast and inexpensive method of detecting a ligand binding to a protein of interest. Briefly, the protein is gradually heated in the presence of a fluorescent dye that binds non-specifically to the hydrophobic surfaces. As the protein unfolds, hydrophobic surfaces within the protein are revealed, causing the dye to fluoresce. A change in the melting temperature (*T*_m_) when the protein is heated in the presence of a ligand, indicates a protein–ligand interaction. Fig. 2 shows the results of the thermal shift assay carried out using recombinant PBP3 and PBP4 with compounds 1–10. The PBPs can be divided into two main categories: high molecular mass (HMM) and low molecular mass (LMM).^13^ PBP3 was chosen as a representative HMM PBP, and PBP4 as a representative LMM PBP. Cephalexin (1) has been previously reported to show good binding affinities for both PBP3 and PBP4.^14^ Penicillin (11) and cefpodoxime (12) were included as positive controls.

The results from this assay showed that all of the analogues tested caused an increase in the *T*_m_ with PBP3, thus increasing the thermostability of this particular PBP upon binding. Whereas, with PBP4 there was either no shift or a small decrease in the *T*_m_ observed, indicating the ligands are facilitating the unfolding of the protein. Previously reported thermal shift assays with PBPs have noted both increases and decreases in *T*_m_ of the protein after the binding of different β-lactam analogues.^15–17^

**Fig. 3:**
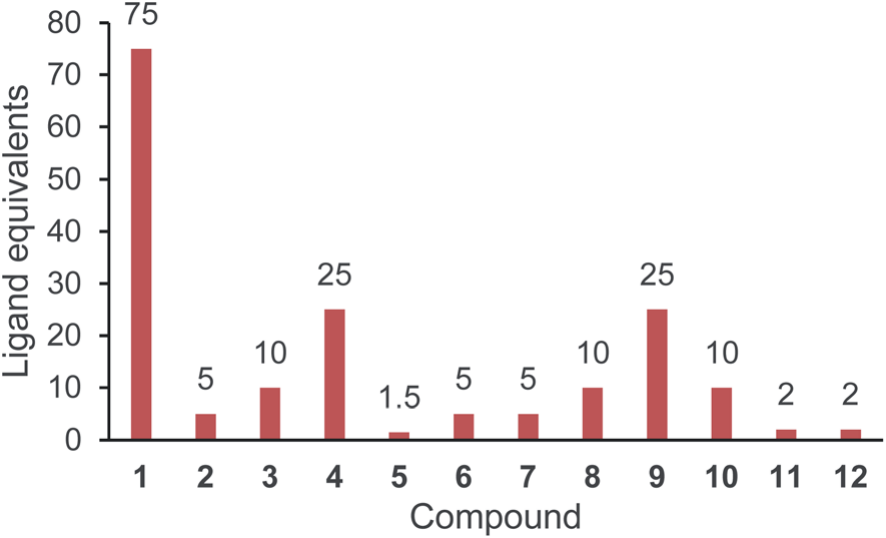
Relative affinities for compounds 1–12 with PBP3. Ligand equivalents required to cause >*T*_m1/2_ shift. Lower equivalents conveys higher affinity. The assay protocol is provided in the Experimental section.

Further thermal shift studies were carried out to approximate the affinities of compounds 1–12 with PBP3. PBP3 was selected for this study as this particular PBP is essential for cell division in *E. coli*, making PBP3 an important target for β-lactam antibiotics.^13^ By measuring the *T*_m_ values of PBP3 with compounds 1–12 at a range of ligand concentrations, after a constant incubation time, the equivalents of each ligand required to cause a shift greater than half the *T*_m_ value (*T*_m1/2_) were determined, Fig. 3. Using this assay, it was determined that 75 equivalents of compound 1 was required to achieve >*T*_m1/2_ but with analogues 2–10, fewer equivalents were required. This study also showed that penicillin (11) has a great affinity for PBP3 than cephalexin (1), which is consistent with previously reported data.^14^ The results, as shown in Fig. 3, suggest that addition of the tethers has improved the binding affinity of analogues 2–10 with PBP3, compared to that of the parent antibiotic 1.

#### β-Lactamase kinetics studies

**Fig. 4:**
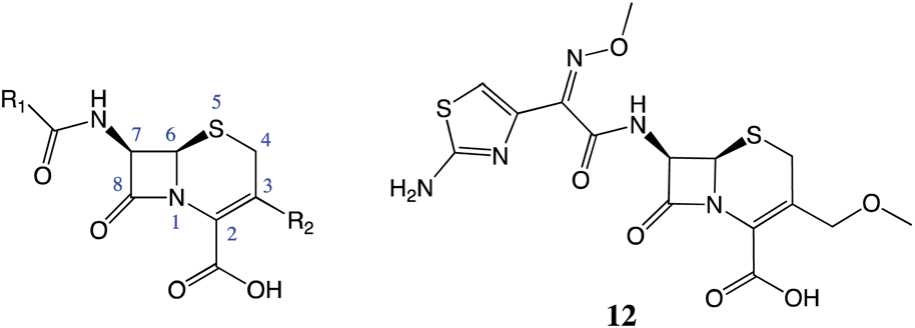
The cephalosporin structure. Cefpodoxime (12), has increased stability in the presence of β-lactamases due to the increased steric bulk at the 7-position.

Previously reported SAR around the β-lactam class of antibiotics has shown that increased steric bulk at the 7-position of cephalosporins can reduce the rates of drug hydrolysis by the β-lactamases.^18^ Many of the β-lactam antibiotics from the later generations, for example cefpodoxime (12, Fig. 4), which are stable in the presence of many β-lactamases, feature sterically demanding groups in this position. The SAR of cephalosporins suggested that increasing the steric bulk at the 7-position of cephalexin (1) will provide resistance to β-lactamase-mediated hydrolysis. Therefore, it was hypothesised that analogues 2–10, which all feature tethers at this position, would have improved stability, compared to that of the parent drug 1.

To investigate the effect that the addition of the chemical tethers reported herein had on the rate of β-lactamase-mediated hydrolysis, a previously reported absorbance assay was employed.^19^ The rates of hydrolysis were measured by the decrease in absorbance at 260 nm caused by hydrolysis of the β-lactam ring. The rates of hydrolysis were measured and the initial velocity of each determined. The high *K*_m_ values prevented determinations of the *V*_max_ values, therefore the catalytic efficiencies (*k*_cat_/*K*_m_) were determined using *v* = (*k*_cat_/*K*_m_)[E][S].^20,21^ The relative catalytic efficiencies, with respect to the (*k*_cat_/*K*_m_) value for the parent antibiotic cephalexin (1), were used to compare the reaction specificities of four selected β-lactamases with the tethered analogues, Table 1.

**Table 1:** Relative catalytic efficiencies (*k*_cat_/*K*_m_)^a^ of compounds 1–10 with TEM-1, CTX-M-15, AmpC, and NDM-1

Compound	TEM-1	CTX-M-15	AmpC	NDM-1
1	1.0	1.0	1.0	1.0
2	0.2	0.2	0.7	1.1
3	1.5	0.3	5.7	1.7
4	1.2	0.3	17	4.9
5	0.6	0.8	11	3.7
6	0.4	0.5	4.3	1.2
7	2.2	0.5	19	5.2
8	1.8	1.0	11	4.7
9	2.6	0.3	4.7	1.3
10	0.3	0.6	13	3.5

a Catalytic efficiencies (*k*_cat_/*K*_m_) were determined using *v* = (*k*_cat_/*K*_m_)[E][S].^20,21^ Relative values calculated as a ratio with respect to cephalexin, 1.

The β-lactamase enzymes are well studied, with over 2500 unique proteins identified.^22^ This vast family of enzymes can be categorised into subsets (class A, B, C, or D), based on their protein sequence. Initial tests were carried out using TEM-1, one of the most common β-lactamases found in Gram-negative bacteria.^23^ This class A β-lactamase is able to hydrolyse penicillins and the early generations of cephalosporins. Of the ten analogues tested, compound 9 was the preferred substrate with TEM-1, suggesting that the large Boc group attached to this analogue does not hinder the hydrolysis. The least preferred substrate with TEM-1 was compound 2, which is the largest of the compounds tested with a PEG tether. However, there was no clear trend in the relative catalytic efficiencies of compounds 1–10 with TEM-1.

As with TEM-1, compound 2 was also observed to be the least preferred substrate with CTX-M-15, an extended spectrum β-lactamase (ESBL) from class A. ESBLs are plasmid-encoded enzymes that confer increased antibiotic resistance to commonly used antibiotics.^24^ CTX-M-14 and CTX-M-15 are the most prevalent ESBLs and known to contribute towards many cases of multidrug resistant infections.^25,26^ The relative catalytic efficiencies showed that the two more preferred substrates of CTX-M-15 had no tether (compound 1) and the smallest tether tested, the acetyl group (compound 8). This trend suggests that modifications to this point of the antibiotic has a direct effect on the stability against hydrolysis by CTX-M-15. Thus structural modifications to this point of the antibiotic could be an effective strategy in the production of antibiotics with increased stability to this ESBL.

The relative catalytic efficiencies of compounds 1–10 with AmpC demonstrated that introduction of the tethers resulted, surprisingly, in increased substrate activity against the modified β-lactam. AmpC is a class C β-lactamase known to confer resistance to the cephalosporins such as cephalexin (1), as well as cephamycins and carbapenems.^22^ Compound 2 was less favoured than 1, but all other analogues had a significantly higher catalytic efficiency than the parent antibiotic, particularly compound 7, which featured a glutaric acid tether. This observation suggests that modifications to this point of the antibiotic results in reduced stability to AmpC mediated hydrolysis. This is in contrast to the trend observed with CTX-M-15, showing that the effect of these structural modifications, to the 7-position of cephalexin (1), is specific to the β-lactamase under investigation.

Further tests were carried out to investigate the effects of the tethers on the hydrolysis by a metallo-β-lactamase, NDM-1. The metallo-β-lactamases are known to hydrolyse penicillins, cephalosporins and even last resort carbapenems. These class B β-lactamases hydrolyse β-lactams by an alternative mechanism that relies on one or two zinc ions present in the active site.^27^ These enzymes are often produced by clinical strains with multiple forms of resistance that are only susceptible to last line antibiotics.^28^ The relative catalytic efficiencies of compounds 1–10 with NDM-1 demonstrated that introduction of the tethers to the antibiotic resulted in increased substrate activity, the same trend that was observed with AmpC. These results suggest that modifications to this point of the antibiotic results in reduced stability to NDM-1 mediated hydrolysis and should be avoided in developing cephalexin (1) analogues stable to NDM-1.

#### *In vivo* assays

The *in vitro* assays demonstrated that the modified analogues were able to interact with the therapeutic target proteins (PBP3 and PBP4) and four selected β-lactamases (TEM-1, CTX-M-15, AmpC, and NDM-1), as shown in Fig. 2, 3 and Table 1. Further testing was carried out *in vivo* to determine whether the tethered compounds had any antibacterial activity. Table 2 summarises the MIC values determined for 1–12, MBC_50_ data is included in the ESI.†

**Table 2:** MIC results for compounds 1–12

Compound	MIC^a^	
	*S. aureus* (NCTC 6571)	*E. coli* (BW25113)
1	18.9 μM	94.9 μM
2	92.6 μM	>400 μM^b^
3	55.8 μM	>400 μM^b^
4	35.0 μM	>400 μM^b^
5	24.9 μM	>400 μM^b^
6	82.9 μM	>400 μM^b^
7	81.4 μM	>400 μM^b^
8	59.0 μM	>400 μM^b^
9	11.0 μM	>400 μM^b^
10	51.4 μM	>400 μM^b^
11^c^	97.7 nM	371 μM
12^c^	20.9 μM	7.7 μM

a MIC values were determined after 16 h incubation. Dose–response curves are provided in the ESI, MIC data was analysed using GraphPad Prism (version 8.3.0).

b MIC not determined in concentration range tested (up to 400 μM).

c Penicillin (11) and cefpodoxime (12).

The activity of 1–12 was investigated with growth assays against *Staphylococcus aureus* (NCTC 6571). *S. aureus* is a virulent Gram-positive pathogen that can cause a wide range of infections in humans.^29,30^ Each compound was tested at 400–6 μM concentrations, with lower concentrations tested as required. All tethered analogues were still able to inhibit the growth of this strain of *S. aureus*; however, the tethers were detrimental to the antimicrobial activity against this Gram-positive bacteria, with all but one MIC value (compound 9) determined to be greater than that of the parent antimicrobial 1.

Further growth assays tested 1–12 against *Escherichia coli* BW25113, a K-12 strain. *E. coli* K-12 is the workhorse of many microbiology laboratories; however, *E. coli* is a versatile Gram-negative bacterium and the evolution of pathogenic *E. coli* can cause a number of harmful infections in humans.^31^ Only the parent antibiotic 1 and the two control compounds 11 and 12 were observed to have antimicrobial activity against *E. coli*. Thus showing that the addition of the chemical tethers has a significant detrimental effect on the activity of the antibiotic against *E. coli*.

#### Physicochemical properties

Since the abilities of 2–10 to bind the therapeutic target had been confirmed *in vitro* (Fig. 2), the *in vivo* results suggest that the tethered analogues may be unable to access the PBPs within the periplasm of *E. coli*. To gain a further insight into the effects of the chemical tethers, the physicochemical properties of each compound was evaluated. The compounds were scored on the properties favourable for accumulation in Gram-negative bacteria, based on the observations of Richter *et al.* who reported that there are three key properties in predicting the accumulation: a primary amine, low globularity, and high rigidity.^32,33^ In order to score the likelihood of the compounds accumulating, each was scored based on the “eNTRy rules” reported by Richter *et al.*: number of primary amines (NoA) ≥ 1; globularity (Glob) ≤ 0.25; number of rotatable bonds (NoRB) ≤ 5, Table 3. The predicted accumulation scores were determined to be below ideal for all of the tethered analogues (2–10), primarily due to the loss of the amine group used in the introduction of the tethers *via* an amide bond. Compound 10, which maintained an amine group, failed based on the increased NoRB. Chemical tethers are often designed with flexibility to favour the subsequent reactions with other substrates, particularly in examples that form a monolayer on a surface. However, this flexibility increases the NoRB, reducing the rigidity of these analogues, which reduces the likelihood of accumulation. Based on the predicted accumulation scores of Table 3, all of the tethered analogues are expected to have lower activity than that of 1 against Gram-negative bacteria. As shown in Table 2, this was found to be true for all tethered analogues, 2–10. Compounds 8 and 10 both scored two but for different reasons: compound 8 lacked the required amine whereas compound 10 exceeded the limit for NoRB. The growth assay results showed that both compounds were inactive against *E. coli* demonstrating the importance of these two criteria of the “eNTRy rules”. By using more rigid tethers and including an amine group, it may be possible to produce tethered analogues that are active against Gram-negative bacteria. However, the criteria compared in Table 3 fail to predict the measured antimicrobial activity of 11 and 12 against *E. coli*. A reliable method of predicting antimicrobial activity is very much coveted, but it is yet to be achieved due to the complex factors involved.^32–35^

**Table 3:** Score for the likelihood of accumulation inside Gram-negative bacteria

Compound	NoA^a^	Glob^b^	NoRB^c^	Score^d^
1	1	0.057	4	3
2	0	0.022	30	1
3	0	0.064	11	1
4	0	0.098	7	1
5	0	0.083	10	1
6	0	0.035	11	1
7	0	0.085	9	1
8	0	0.134	5	2
9	0	0.133	7	1
10	1	0.088	9	2
11^e^	0	0.095	4	2
12^e^	0	0.068	7	1

a Number of primary amines (NoA).

b Globularity (Glob).

c Number of rotatable bonds (NoRB).

d Score out of three based on the following criteria: NoA ≥ 1; Glob ≤ 0.25; NoRB ≤ 5.

e Penicillin (11) and cefpodoxime (12) included for comparison. NoRB determined using Marvin 17.21.0, ChemAxon. Glob determined using https://entry-way.org.

## Summary and conclusions

A series of cephalexin (1) analogues equipped with chemical tethers was evaluated for binding to the therapeutic target, the penicillin binding proteins, and shown to maintain binding interactions *in vitro*. Further investigations with four β-lactamases (TEM-1, CTX-M-15, AmpC, and NDM-1) were carried out and revealed that the modifications affected each enzyme's catalytic rates differently. With TEM-1, there was no clear trend in the catalytic efficiencies of 1–10. CTX-M-15 was found to favour hydrolysis of the parent antibiotic without a tether, thus demonstrating that modifications to this position of 1 could produce antibiotics with increased stability to this ESBL. Conversely, both AmpC and NDM-1 were found to favour the modified analogues suggesting that these types of structural modifications should be avoided in the design of analogues stable to AmpC and/or NDM-1. The tethers were found to lower the antimicrobial activities when testing against *S. aureus* and cause complete loss of activity against *E. coli*. The loss of activity against *E. coli* was consistent with previously reported observations linking the globularity, rigidity, and amine functionality of antibiotics with accumulation in Gram-negatives.

These results show that the addition of the tethers directly affected the properties of the antibiotic, thus highlighting the importance of evaluating the changes that occur from modifying molecular probes. Most notably, the effect of the tethers on the rate of β-lactamase-mediated hydrolysis was specific to the β-lactamase under investigation. This suggests that modifying the 7-position of 1 could be key in the development of surface-bound antibiotics for the selective detection of β-lactamases associated with multidrug resistant infections, such as NDM-1.

## Experimental

### Chemistry

#### General experimental

Analytical thin layer chromatography (TLC) was performed with EM Science silica gel 60 F254 aluminium plates. Visualisation was carried out using a UV lamp (254 nm) and by immersion in potassium permanganate (KMnO_4_), followed by heating using a heat gun. Organic solutions were concentrated by rotary evaporation at 40–45 °C. Purification of reaction products by flash column chromatography was carried out using Fluka Silica, pore size 60 Å, 220–440 mesh, 35–75 μm.

#### Materials

Unless otherwise noted, all purchased materials were used without purification. All standard solvents were purchased from Sigma Aldrich. All standard acids, bases, and drying agents were purchased from Fisher Scientific. NHS and *N*,*N*-diisopropylethylamine (DIPEA) were purchased from Acros Organics. Cephalexin monohydrate and Boc_2_O were purchased from Fluorochem. Pentynoic acid, *N*,*N*′-dicyclohexylcarbodiimide (DCC), lipoic acid, glutaric anhydride, oxalyl chloride, and Et_3_N were purchased from Sigma Aldrich. DMAP and 5-(Boc-amino)pentanoic acid were purchased from TCI.

#### Instrumentation

^1^H and ^13^C NMR spectra were recorded on a Jeol ECS 400 (400 MHz for ^1^H, 101 MHz for ^13^C) at ambient temperature. Chemical shifts are reported relative to residual solvent peaks and coupling constants (*J*) are given in hertz. High-resolution ESI mass spectra were recorded on a Bruker microTOF electrospray mass spectrometer. Infrared (IR) spectra were recorded on a PerkinElmer Spectrum Two (ATIR). Analytical HPLC measurements were performed on a Shimadzu HPLC system (Prominence) equipped with a LC-20AD pump, SIL-20A autosampler, DGU-20AS degasser, CTO-20AC column oven, CBM-20A communication bus module and SPD-M20A diode array detector using an Athena C18-WP column (100 Å, 4.6 × 250 mm, 5 μm). Eluent gradient: 5–95% MeCN/H_2_O with a 0.1% formic acid modifier, over 15 minutes.

#### Compound synthesis

##### Preparations of 2 and 3

Compound 2 (6*R*,7*R*)-7-((*R*)-30-(2,5-Dioxo-2,5-dihydro-1*H*-pyrrol-1-yl)-4,8,25-trioxo-2-phenyl-6,12,15,18,21-pentaoxa-3,9,24-triazatriacontanamido)-3-methyl-8-oxo-5-thia-1-azabicyclo[4.2.0]oct-2-ene-2-carboxylic acid and 3, (6*R*,7*R*)-7-((*R*)-2-(6-(2,5-dioxo-2,5-dihyrdo-1*H*-pyrrol-1-yl)hexanamido)-2-phenylacetamido)-3-methyl-8-oxo-5-thia-1-azabicyclo[4.2.0]oct-2-ene-2-carboxylic acid were previously reported.^7^

##### Preparation of 4

(6*R*,7*R*)-3-Methyl-8-oxo-7-[(2*R*)-2-(pent-4-ynamido)-2-phenylacetamido]-5-thia-1-azabicyclo[4.2.0]oct-2-ene-2-carboxylic acid. To a stirred solution of pentynoic acid (15) (100 mg, 1.020 mmol, 1 equiv.) in anhydrous DCM (1 mL, 1 M) at 0 °C was added NHS (123 mg, 1.071 mmol, 1.05 equiv.). A solution of DCC (221 mg, 1.071 mmol, 1.05 equiv.) in DCM (1 mL) was added slowly. The reaction mixture was then allowed to warm to room temperature. After 16 h, the urea precipitate formed during the reaction was filtered off, and the filter cake washed with DCM. The filtrate was concentrated under reduced pressure to afford 2,5-dioxopyrrolidin-1-yl pent-4-ynoate (16), which was used without further purification. The residue was dissolved in anhydrous MeCN (10 mL) and cephalexin monohydrate (338 mg, 0.927 mmol, 0.9 equiv.) was added. The mixture was cooled in an ice bath followed by the slow addition of DIPEA (443 μL, 2.550 mmol, 2.5 equiv.). Once the addition was complete, the ice bath was removed, and the reaction was allowed to stir at room temperature for 2 h at which time the reaction appeared complete by TLC. The reaction mixture was concentrated under reduced pressure, dissolved in EtOAc (20 mL), and washed with 0.1 M HCl (2 × 20 mL). The organic layer was collected and concentrated to a cream solid, which was the purified by trituration from diethyl ether to afford (6*R*,7*R*)-3-methyl-8-oxo-7-[(2*R*)-2-(pent-4-ynamido)-2-phenylacetamido]-5-thia-1-azabicyclo[4.2.0]oct-2-ene-2-carboxylic acid (4) (277 mg, 70% yield) as a white solid. ^1^H NMR (400 MHz, DMSO-*d*_6_) *δ* 9.32 (d, *J* = 8.4 Hz, 1H), 8.65 (d, *J* = 8.2 Hz, 1H), 7.44 (d, *J* = 7.0 Hz, 2H), 7.37–7.25 (m, 3H), 5.71 (d, *J* = 8.2 Hz, 1H), 5.62 (dd, *J* = 8.3, 4.7 Hz, 1H), 4.96 (d, *J* = 4.7 Hz, 1H), 3.46 (d, *J* = 18.5 Hz, 1H), 3.28 (d, *J* = 18.4 Hz, 1H), 2.76 (t, *J* = 2.6 Hz, 1H), 2.47–2.40 (m, 2H), 2.39–2.31 (m, 2H), 1.98 (s, 3H). ^13^C NMR (101 MHz, DMSO-*d*_6_) *δ* 170.8, 170.2, 164.1, 163.6, 138.3, 129.7, 128.2, 127.6, 127.1, 122.8, 83.8, 71.4, 58.4, 57.2, 55.6, 33.7, 28.9, 19.4, 14.1. HRMS: exact mass calculated for [M − H]^−^ (C_21_H_20_N_3_O_5_S) requires *m*/*z* 426.1129, measured *m*/*z* 426.1148. IR (neat): 3280, 3010, 1762, 1721, 1650, 1643, 1539, 1377, 1218, 1108, 1070 cm^−1^. HPLC purity (254 nm): 96%.

##### Preparation of 5

(6*R*,7*R*)-7-[(2*R*)-2-[5-(1,2-Dithiolan-3-yl)pentanamido]-2-phenylacetamido]-3-methyl-8-oxo-5-thia-1-azabicyclo[4.2.0]oct-2-ene-2-carboxylic acid. To a stirred solution of lipoic acid (17) (1 g, 4.854 mmol, 1 equiv.) in anhydrous DCM (30 mL, 0.2 M) at 0 °C was added NHS (614 mg, 5.340 mmol, 1.1 equiv.). A solution of DCC (1.3 g, 6.311 mmol, 1.3 equiv.) in DCM (10 mL) was added slowly. The reaction mixture was then allowed to warm to room temperature. After 16 h, the urea precipitate formed during the reaction was filtered off, and the filter cake washed with DCM. The filtrate was concentrated under reduced pressure to afford a yellow residue. Recrystallisation from EtOAc : hexane (1 : 1) afforded 2,5-dioxopyrrolidin-1-yl 5-(1,2-dithiolan-3-yl)pentanoate (18) (978 mg, 66%) as an off-white solid. ^1^H NMR (400 MHz, chloroform-*d*) *δ* 3.58 (dq, *J* = 8.2, 6.4 Hz, 1H), 3.25–3.06 (m, 2H), 2.84 (d, *J* = 4.1 Hz, 4H), 2.63 (t, *J* = 7.3 Hz, 2H), 2.47 (dtd, *J* = 13.1, 6.6, 5.4 Hz, 1H), 1.93 (dq, *J* = 12.7, 6.9 Hz, 1H), 1.79 (p, *J* = 7.4 Hz, 2H), 1.77–1.65 (m, 2H), 1.63–1.52 (m, 2H). ^13^C NMR (101 MHz, chloroform-*d*) *δ* 169.3, 168.6, 56.2, 40.3, 38.7, 34.6, 30.9, 28.5, 25.7, 24.5. ^1^H and ^13^C are consistent with previously reported data.^36^ HRMS: exact mass calculated for [M + Na]^+^ (C_12_H_17_NNaO_4_S_2_) requires *m*/*z* 326.0491, measured *m*/*z* 326.0490. IR (neat): 2934, 2915, 2861, 1809, 1780, 1729, 1630, 1574 cm^−1^. Mp 93–94 °C, consistent with reported data.^37^

2,5-Dioxopyrrolidin-1-yl 5-(1,2-dithiolan-3-yl)pentanoate (18) (294 mg, 0.971 mmol, 1 equiv.) was dissolved in anhydrous MeCN (20 mL, 0.05 M) and cephalexin monohydrate (235 mg, 0.647 mmol, 0.66 equiv.) was added. The mixture was cooled in an ice bath followed by the slow addition of DIPEA (169 μL, 0.971 mmol, 1 equiv.). Once the addition was complete, the ice bath was removed and the reaction was allowed to stir at room temperature for 6 h. The reaction mixture was then concentrated under reduced pressure and triturated using 5% DCM/diethyl ether to afford (6*R*,7*R*)-7-[(2*R*)-2-[5-(1,2-dithiolan-3-yl)pentanamido]-2-phenylacetamido]-3-methyl-8-oxo-5-thia-1-azabicyclo[4.2.0]oct-2-ene-2-carboxylic acid (5) (190 mg, 55%) as a white solid. ^1^H NMR (400 MHz, DMSO-*d*_6_) *δ* 9.29 (d, *J* = 8.3 Hz, 1H), 8.55 (d, *J* = 8.3 Hz, 1H), 7.48–7.39 (m, 2H), 7.35–7.23 (m, 3H), 5.70 (d, *J* = 8.3 Hz, 1H), 5.61 (dd, *J* = 8.4, 4.7 Hz, 1H), 4.95 (d, *J* = 4.7 Hz, 1H), 3.65–3.54 (m, 1H), 3.46 (d, *J* = 18.2 Hz, 1H), 3.27 (d, *J* = 18.2 Hz, 1H), 3.22–3.06 (m, 2H), 2.40 (dt, *J* = 12.5, 6.2 Hz, 1H), 2.22 (t, *J* = 7.4 Hz, 2H), 1.98 (s, 3H), 1.90–1.79 (m, 1H), 1.76–1.57 (m, 2H), 1.57–1.46 (m, 2H), 1.34 (q, *J* = 7.4, 6.9 Hz, 2H). ^13^C NMR (101 MHz, DMSO-*d*_6_) *δ* 171.9, 170.9, 164.0, 163.6, 138.4, 128.2, 127.6, 127.1, 58.4, 57.2, 56.2, 55.5, 38.1, 34.7, 34.1, 33.4, 28.9, 28.3, 25.3, 25.0, 24.5, 19.4. HRMS: exact mass calculated for [M + Na]^+^ (C_24_H_29_N_3_NaO_5_S_3_) requires *m*/*z* 558.1162, measured *m*/*z* 558.1152. IR (neat): 3287, 2927, 1766, 1642, 1513, 1360, 1219 cm^−1^. HPLC purity (254 nm): 95%.

##### Preparation of 6

(6*R*,7*R*)-7-[(2*R*)-2-{5-[(2,5-Dioxopyrrolidin-1-yl)oxy]-5-oxopentanamido}-2-phenylacetamido]-3-methyl-8-oxo-5-thia-1-azabicyclo[4.2.0]oct-2-ene-2-carboxylic acid. NHS (1 g, 8.772 mmol, 1 equiv.) and DMAP (1 g, 8.772 mmol, 1 equiv.) were dissolved in anhydrous THF (50 mL) and cooled in an ice bath. Glutaric anhydride (19) (1.6 g, 13.158 mmol, 1.5 equiv.) was then added portion-wise and the resulting reaction mixture was allowed to warm to room temperature. After a further 4 h at room temperature, the reaction was concentrated under reduced pressure. The residue was dissolved in EtOAc (60 mL) and washed with 0.1 M HCl (2 × 30 mL) followed by brine (1 × 30 mL). The organic layer was concentrated to afford 5-[(2,5-dioxopyrrolidin-1-yl)oxy]-5-oxopentanoic acid (20) (592 mg) as a colourless oil, which was used without further purification. The prepared NHS ester (20) (500 mg, 2.183 mmol, 1 equiv.) was then dissolved in anhydrous DCM (10 mL) under an atmosphere of N_2_ and cooled in an ice bath. Oxalyl chloride (225 μL, 2.621 mmol, 1.2 equiv.) was then added followed by one drop of DMF. The resultant reaction mixture was allowed to return to room temperature and stirred for 16 h, after which it was concentrated under reduced pressure to afford the acid chloride. In a separate flask, cephalexin monohydrate (598 mg, 1.637 mmol, 0.75 equiv.) was suspended in anhydrous MeCN (20 mL) then anhydrous pyridine (329 μL, 4.093 mmol, 1.9 equiv.) was added. The previously prepared acid chloride was then dissolved in anhydrous MeCN (10 mL) and added to the cephalexin/pyridine mixture. The reaction was then stirred at room temperature for 24 h after which it was concentrated under reduced pressure and the residue was triturated in EtOAc to afford a cream solid. The solid was then recrystallised from MeCN to afford (6*R*,7*R*)-7-[(2*R*)-2-{5-[(2,5-dioxopyrrolidin-1-yl)oxy]-5-oxopentanamido}-2-phenylacetamido]-3-methyl-8-oxo-5-thia-1-azabicyclo[4.2.0]oct-2-ene-2-carboxylic acid (6) (501 mg, 52% over 3 steps) as a cream solid. ^1^H NMR (400 MHz, DMSO-*d*_6_) *δ* 9.27 (dd, *J* = 8.3, 3.1 Hz, 1H), 8.57 (dd, *J* = 21.1, 8.1 Hz, 1H), 7.43 (d, *J* = 7.1 Hz, 1H), 7.38–7.25 (m, 3H), 5.67 (d, *J* = 8.0 Hz, 1H), 5.61 (dd, *J* = 8.3, 4.6 Hz, 1H), 4.95 (d, *J* = 4.6 Hz, 1H), 3.46 (d, *J* = 18.5 Hz, 1H), 3.27 (d, *J* = 18.3 Hz, 1H), 2.81 (s, 4H), 2.73–2.63 (m, 2H), 2.33 (t, *J* = 7.3 Hz, 2H), 2.27–2.16 (m, 2H), 1.98 (s, 3H). ^13^C NMR (101 MHz, DMSO-*d*_6_) *δ* 171.2, 170.9, 170.3, 168.8, 168.5, 164.1, 163.5, 138.2, 129.8, 128.2, 127.6, 127.2, 122.7, 58.4, 57.2, 55.7, 33.4, 29.7, 28.9, 25.5, 20.5, 19.4. HRMS: exact mass calculated for [M + Na]^+^ (C_25_H_26_N_4_NaO_9_S) requires *m*/*z* 581.1313, measured *m*/*z* 581.1322. IR (neat): 3286, 2945, 1776, 1728, 1643, 1524, 1360, 1204, 1158, 1066 cm^−1^. QNMR purity (^1^H NMR, maleic acid reference, DMSO-*d*_6_): 90%.

##### Preparation of 7

(6*R*,7*R*)-7-[(2*R*)-2-(4-Carboxybutanamido)-2-phenylacetamido]-3-methyl-8-oxo-5-thia-1-azabicyclo[4.2.0]oct-2-ene-2-carboxylic acid. A solution of cephalexin monohydrate (100 mg, 0.274 mmol, 1 equiv.) and Et_3_N (46 μL, 0.329 mmol, 1.2 equiv.) was stirred at room temperature for 10 min. Glutaric anhydride (19) (38 mg, 0.329 mmol, 1.2 equiv.) was then added and the resultant reaction mixture stirred for 3 h at room temperature. The reaction mixture was then concentrated under reduced pressure to afford a yellow solid, which was then triturated using 10% DCM/diethyl ether to afford the Et_3_N salt of (6*R*,7*R*)-7-[(2*R*)-2-(4-carboxybutanamido)-2-phenylacetamido]-3-methyl-8-oxo-5-thia-1-azabicyclo[4.2.0]oct-2-ene-2-carboxylic acid (7) (144 mg, 94%), as a cream solid. ^1^H NMR (400 MHz, acetonitrile-*d*_3_) *δ* 8.18 (t, *J* = 8.4 Hz, 1H), 7.58 (t, *J* = 8.5 Hz, 1H), 7.44–7.36 (m, 2H), 7.35–7.27 (m, 3H), 5.77 (dd, *J* = 7.7, 4.3 Hz, 1H), 5.61 (dd, *J* = 9.2, 4.7 Hz, 1H), 4.83 (d, *J* = 4.7 Hz, 1H), 3.39 (d, *J* = 17.4 Hz, 1H), 3.11–2.98 (m, 7H), 2.37–2.24 (m, 4H), 1.87 (s, 3H), 1.84–1.73 (m, 2H), 1.22 (t, *J* = 7.3 Hz, 9H). ^13^C NMR (101 MHz, DMSO-*d*_6_) *δ* 174.4, 171.6, 170.9, 165.4, 162.8, 138.4, 128.2, 127.9, 127.5, 127.2, 58.1, 56.9, 55.6, 45.0, 34.1, 33.3, 28.5, 20.8, 19.3, 9.0. HRMS: exact mass calculated for [M + H]^+^ (C_21_H_24_N_3_O_7_S) requires *m*/*z* 462.1329, measured *m*/*z* 462.1331. IR (neat): 3260, 2987, 1770, 1682, 1635, 1532, 1383, 1351, 1276, 1208, 1154 cm^−1^. HPLC purity (254 nm): 95%.

##### Preparation of 8

(6*R*,7*R*)-7-[(2*R*)-2-Acetamido-2-phenylacetamido]-3-methyl-8-oxo-5-thia-1-azabicyclo[4.2.0]oct-2-ene-2-carboxylic acid. A solution of cephalexin monohydrate (200 mg, 0.548 mmol, 1 equiv.) and DIPEA (143 μL, 0.822 mmol, 1.5 equiv.) was stirred at room temperature for 10 min. Acetic anhydride (62 μL, 0.658 mmol, 1.2 equiv.) was then added and the resultant reaction mixture was stirred at room temperature for 4 h. The reaction mixture was then concentrated under reduced pressure to afford a cream solid, which was then triturated using diethyl ether to afford (6*R*,7*R*)-7-[(2*R*)-2-acetamido-2-phenylacetamido]-3-methyl-8-oxo-5-thia-1-azabicyclo[4.2.0]oct-2-ene-2-carboxylic acid (8) (220 mg, quantitative) as a white solid. ^1^H NMR (400 MHz, DMSO-*d*_6_) *δ* 9.26 (d, *J* = 8.3 Hz, 1H), 8.59 (d, *J* = 8.4 Hz, 1H), 7.43 (d, *J* = 6.9 Hz, 2H), 7.36–7.22 (m, 3H), 5.69 (d, *J* = 8.3 Hz, 1H), 5.55 (dd, *J* = 8.3, 4.6 Hz, 1H), 4.91 (d, *J* = 4.7 Hz, 1H), 3.41 (d, *J* = 17.5 Hz, 1H), 3.19 (d, *J* = 17.9 Hz, 1H), 1.94 (s, 3H), 1.91 (s, 3H). ^13^C NMR (101 MHz, DMSO-*d*_6_) *δ* 170.9, 169.0, 164.0, 163.5, 138.4, 128.2, 127.6, 127.2, 58.3, 57.1, 55.6, 28.7, 22.4, 19.4. Two carbon resonances not observed/coincident. HRMS: exact mass calculated for [M + H]^+^ (C_18_H_20_N_3_O_5_S) requires *m*/*z* 390.1116, measured *m*/*z* 390.1115. IR (neat): 3283, 1761, 1640, 1538, 1497, 1374, 1298, 1188, 1125 cm^−1^. HPLC purity (254 nm): 98%.

##### Preparation of 9

(6*R*,7*R*)-7-[(2*R*)-2-{[(*Tert*-butoxy)carbonyl]amino}-2-phenylacetamido]-3-methyl-8-oxo-5-thia-1-azabicyclo[4.2.0]oct-2-ene-2-carboxylic acid. A solution of cephalexin monohydrate (500 mg, 1.370 mmol, 1 equiv.) and Et_3_N (190 μL, 1.370 mmol, 1 equiv.) was stirred at room temperature for 10 min. Boc_2_O (388 mg, 1.781 mmol, 1.3 equiv.) was then added and the resultant reaction mixture was stirred at room temperature for 3 h. The reaction mixture was then concentrated under reduced pressure and the residue dissolved in DCM (20 mL). This organic solution was then washed with 0.1 M HCl (2 × 20 mL) and brine (1 × 20 mL). The organic layer was collected, dried (MgSO_4_) and concentrated under reduced pressure. The residue was then triturated using diethyl ether to afford (6*R*,7*R*)-7-[(2*R*)-2-{[(*tert*-butoxy)carbonyl]amino}-2-phenylacetamido]-3-methyl-8-oxo-5-thia-1-azabicyclo[4.2.0]oct-2-ene-2-carboxylic acid (9) (608 mg, 93%) as a cream solid. ^1^H NMR (400 MHz, chloroform-*d*) *δ* 9.57 (s, 1H), 7.45–7.23 (m, 5H), 7.18 (s, 1H), 6.07 (d, *J* = 7.2 Hz, 1H), 5.65 (s, 1H), 5.34 (d, *J* = 7.3 Hz, 1H), 4.90 (d, *J* = 4.5 Hz, 1H), 3.38 (d, *J* = 18.3 Hz, 1H), 3.03 (d, *J* = 18.4 Hz, 1H), 2.09 (s, 3H), 1.40 (s, 9H). ^1^H consistent with previously reported data.^38^^13^C NMR (101 MHz, chloroform-*d*) *δ* 171.1, 164.2, 163.7, 155.8, 138.0, 131.9, 129.1, 128.6, 127.4, 122.6, 81.1, 59.2, 57.9, 57.6, 30.3, 28.4, 20.1. HRMS: exact mass calculated for [M + H]^+^ (C_21_H_26_N_3_O_6_S) requires *m*/*z* 448.1537, measured *m*/*z* 448.1546. IR (neat): 3324, 2977, 1771, 1682, 1496, 1454, 1366, 1237, 1160, 1049 cm^−1^. HPLC purity (254 nm): 98%.

##### Preparation of 10

(6*R*,7*R*)-7-[(2*R*)-2-(5-Aminopentanamido)-2-phenylacetamido]-3-methyl-8-oxo-5-thia-1-azabicyclo[4.2.0]oct-2-ene-2-carboxylic acid. To a stirred solution of 5-(Boc-amino)pentanoic acid (21) (1 g, 4.608 mmol, 1 equiv.) in anhydrous DCM (30 mL, 0.2 M) at 0 °C, was added NHS (583 mg, 5.069 mmol, 1.1 equiv.). A solution of DCC (1.2 g, 5.991 mmol, 1.3 equiv.) in DCM (10 mL) was added slowly. The reaction mixture was then allowed to warm to room temperature. After 16 h, the reaction was filtered and the filter cake washed with DCM. The filtrate was concentrated under reduced pressure and the residue was purified by column chromatography, eluting with 10–60% EtOAc/petroleum ether, to afford a white oil. Trituration in diethyl ether afforded 2,5-dioxopyrrolidin-1-yl 5-{[(*tert*-butoxy)carbonyl]amino}pentanoate (22) (1.37 g, 95%) as a white solid. ^1^H NMR (400 MHz, chloroform-*d*) *δ* 4.59 (br. s, 1H), 3.16 (q, *J* = 6.7 Hz, 2H), 2.84 (d, *J* = 3.7 Hz, 4H), 2.64 (t, *J* = 7.3 Hz, 2H), 1.78 (p, *J* = 7.4 Hz, 2H), 1.60 (p, *J* = 7.0 Hz, 2H), 1.44 (s, 9H). ^1^H is consistent with previously reported data.^39^^13^C NMR (101 MHz, chloroform-*d*) *δ* 169.3, 168.6, 156.1, 79.4, 40.0, 34.1, 29.2, 28.5, 25.7, 25.1, 21.9. HRMS: exact mass calculated for [M + H]^+^ (C_14_H_22_N_2_NaO_6_) requires *m*/*z* 337.1370, measured *m*/*z* 337.1371. IR (neat): 3318, 2975, 2932, 2851, 1812, 1777, 1726, 1682, 1626, 1572, 1514, 1364, 1274, 1201, 1170, 1069, 1056, 1008 cm^−1^.

Et_3_N (114 μL, 0.822 mmol, 1.5 equiv.) was added to a suspension of cephalexin monohydrate (200 mg, 0.548 mmol, 1 equiv.) in MeCN (50 mL). After 5 min dissolution occurred to give a yellow solution. 2,5-Dioxopyrrolidin-1-yl 5-{[(*tert*-butoxy)carbonyl]amino}pentanoate (22) (258 mg, 0.822 mmol, 1.5 equiv.) was then added and the reaction was allowed to stir at room temperature for 16 h. The reaction mixture was concentrated under reduced pressure and the residue dissolved in EtOAc. The organic solution was washed with 0.01 M HCl (3 × 20 mL), then dried (MgSO_4_), and concentrated under reduced pressure to afford a pale yellow solid. Trituration in diethyl ether afforded (6*R*,7*R*)-7-[(2*R*)-2-(5-{[(*tert*-butoxy)carbonyl]amino}pentanamido)-2-phenylacetamido]-3-methyl-8-oxo-5-thia-1-azabicyclo[4.2.0]oct-2-ene-2-carboxylic acid (23) (169 mg, 56%) as a cream solid. ^1^H NMR (400 MHz, DMSO-*d*_6_) *δ* 9.27 (d, *J* = 8.3 Hz, 1H), 8.49 (d, *J* = 8.3 Hz, 1H), 7.41 (d, *J* = 7.2 Hz, 2H), 7.34–7.22 (m, 3H), 6.77 (t, *J* = 5.7 Hz, 1H), 5.67 (d, *J* = 8.2 Hz, 1H), 5.60 (dd, *J* = 8.3, 4.6 Hz, 1H), 4.94 (d, *J* = 4.6 Hz, 1H), 3.45 (d, *J* = 18.3 Hz, 1H), 3.27 (d, *J* = 18.3 Hz, 1H), 2.86 (q, *J* = 6.6 Hz, 2H), 2.18 (t, *J* = 7.3 Hz, 2H), 1.97 (s, 3H), 1.44 (p, *J* = 7.0 Hz, 2H), 1.35 (s, 9H), 1.33–1.27 (m, 2H). ^13^C NMR (101 MHz, DMSO-*d*_6_) *δ* 172.0, 170.9, 164.1, 163.5, 155.6, 138.4, 129.8, 128.2, 127.6, 127.1, 122.7, 77.4, 58.4, 57.2, 55.5, 47.5, 34.6, 29.2, 28.9, 28.3, 22.7, 19.4. HRMS: exact mass calculated for [M + Na]^+^ (C_26_H_34_N_4_NaO_7_S) requires *m*/*z* 569.2040, measured *m*/*z* 569.2041. IR (neat): 3284, 2931, 1764, 1639, 1520, 1452, 1364, 1221, 1164, 1067, 1040 cm^−1^.

To a solution of (6*R*,7*R*)-7-[(2*R*)-2-(5-{[(*tert*-butoxy)carbonyl]amino}pentanamido)-2-phenylacetamido]-3-methyl-8-oxo-5-thia-1-azabicyclo[4.2.0]oct-2-ene-2-carboxylic acid (23) (100 mg, 0.183 mmol, 1 equiv.) in DCM (4 mL, 0.04 M) was added and 0.1 mL triethyl silane followed by 0.5 mL TFA. The reaction mixture was stirred at room temperature for 3 h, after which it was concentrated to an orange oil. The residue was then azeotroped with DCM (3 × 10 mL) to afford a cream solid. This solid was triturated with diethyl ether to afford (6*R*,7*R*)-7-[(2*R*)-2-(5-aminopentanamido)-2-phenylacetamido]-3-methyl-8-oxo-5-thia-1-azabicyclo[4.2.0]oct-2-ene-2-carboxylic acid (10) (77 mg, 94%) as a cream solid. ^1^H NMR (400 MHz, DMSO-*d*_6_) *δ* 9.31 (d, *J* = 8.4 Hz, 1H), 8.57 (d, *J* = 8.2 Hz, 1H), 7.67 (br. s, 2H), 7.47–7.41 (m, 2H), 7.35–7.26 (m, 3H), 5.69 (d, *J* = 8.2 Hz, 1H), 5.62 (dd, *J* = 8.4, 4.6 Hz, 1H), 4.96 (d, *J* = 4.7 Hz, 1H), 3.47 (d, *J* = 18.8 Hz, 1H), 3.28 (d, *J* = 18.2 Hz, 1H), 2.77 (br. s, 2H), 2.31–2.22 (m, 2H), 1.98 (s, 3H), 1.59–1.48 (m, 4H). ^13^C NMR (101 MHz, DMSO-*d*_6_) *δ* 171.6, 170.9, 164.1, 163.5, 138.3, 129.6, 128.2, 127.6, 127.1, 122.7, 58.4, 57.2, 55.5, 38.7, 34.1, 28.9, 26.7, 22.1, 19.4. HRMS: exact mass calculated for [M + H]^+^ (C_21_H_27_N_4_O_5_S) requires *m*/*z* 447.1697, measured *m*/*z* 447.1698. IR (neat): 3250, 3050, 2939, 1763, 1639, 1525, 1363, 1184, 1130, 1070 cm^−1^. HPLC purity (254 nm): 97%.

### Biology

#### Materials

AmpC (Uniprot ID: P00811) was purchased from Abcam plc (Cambridge, UK). PBP4 (Uniprot ID: P32959) was purchased from Generon (Slough, UK).

#### Expression and purification of PBP3

A truncated version of the ftsI gene formed of residues W44-S588, encoding only the soluble domain, was amplified from *E. coli* BW25113. The resulting construct was inserted into the vector pBADnLIC2005,^40^ introducing an N-terminal deca-histidine tag when expressed. The resulting vector was transformed into *E. coli* MC1061 for expression. Cultures for protein expression were grown in 1 L of Luria–Bertani (LB) broth at 37 °C on an orbital shaker. Expression was induced by addition of 0.01% l-arabinose during mid-log phase of growth. Cultures were further incubated for 16 h at 20 °C, then the cells were harvested by centrifugation. Cell pellets were resuspended in 50 mM KPi pH 7.8, 200 mM NaCl, 10 mM imidazole, 20% glycerol with 1 mM phenylmethylsulfonyl fluoride followed by sonication. The lysate was clarified by centrifugation before loading onto a HisTrap HF column (GE Healthcare). To remove any pre-bound ligands, refolding purification was performed by initially washing with the protein unfolding buffer [2 M guanidine HCl, 50 mM KPi (pH 7.8), 200 mM NaCl, 20% glycerol, and 20 mM imidazole] and then performing a gradient wash to the protein refolding buffer [50 mM KPi (pH 7.8), 200 mM NaCl, 20% glycerol, and 20 mM imidazole] before the elution of the protein using the elution buffer [50 mM KPi (pH 7.8), 200 mM NaCl, 20% glycerol, and 500 mM imidazole]. The eluted protein was buffer-exchanged to the buffer 50 mM KPi (pH 7.8), 200 mM NaCl using a HisTrap Desalting (GE Healthcare) column.

#### Thermal shift assay

The thermal shift assay was carried out using the Protein Thermal Shift™ assay kit (Applied Biosystems). 3 μM of purified *E. coli* PBP3 was incubated with the β-lactam analogues at 300 μM concentration in a mixture containing the Protein Thermal Shift™ Dye. The samples were then heated in a StepOnePlus™ Real-Time PCR System from 25 to 95 °C at a rate of 1 °C min^−1^. Tests were carried out in triplicate and the averages plotted as the negative first derivative *vs.* temperature. Reference wells, *i.e.* solutions consisting only of only PBP3 with dye, PBP3 only, and dye only, were used as controls. Melting temperature (*T*_m_) values were determined with and without each compound, and the change in melting temperature (Δ*T*_m_) was obtained, Fig. 2. The thermal shift assay with PBP4 was carried out using the protocol described for PBP3, with the follow deriviatisation: 500 nM of purified *Bacillus subtilis* PBP4 was incubated with 50 μM of the β-lactam analogues.

To determine the approximate affinity of PBP3 for compounds 1 to 12, 2 μM of purified *E. coli* PBP3 was incubated with a range of concentrations of each compound. The mixtures were incubated at room temperature for 25 minutes prior to the start of the protein thermal shift assay program on the qPCR machine. The signals from the hydrophobic fluorescent dye were monitored as the mixtures were heated from 35 to 70 °C at a ramp rate of 0.3%. The *T*_m_ values of PBP3 incubated with each compound at the various concentrations were recorded. The relative changes in the *T*_m_ values, relative to the highest *T*_m_ change seen for each compound, are reported as a ratio in the ESI.† The concentration at which the *T*_m_ rose to >*T*_m1/2_ was used to approximate the relative affinities.

#### Expression and purification of TEM-1

TEM-1 (Uniprot ID: A5PHA6) was cloned into pBKR, a pBADcLIC2005 derivative with a kanamycin resistance cassette in place of the ampicillin resistance cassette. The resulting plasmid, pBKR-TEM1, was transformed into the expression strain *E. coli* MC1061. Starting cultures were grown in 10 mL LB broth at 37 °C overnight with shaking. A litre of expression culture was prepared and grown to an OD_600_ of between 0.4–0.6 before induction with 0.01% (w/v) l-arabinose. The induced culture was allowed to grow for 18 h at 30 °C. The resulting culture was harvested by centrifugation at 5000*g*. The pellet was then resuspended in 30 mL of sterile SET buffer (0.5 M sucrose, 5 mM EDTA, 50 mM Tris–HCl pH 7.8). 13 mg of lysozyme was added to the mixture before incubation for 1 h at 30 °C. To isolate the periplasmic fraction, the treated sample was then clarified by centrifugation at 27 000*g*. The supernatant was dialysed into 50 mM KPi pH 7.8, 200 mM NaCl over 18 hours at 4 °C. After dialysis, the periplasmic fraction was loaded onto an equilibrated 5 mL HisTrap column. The bound His-tagged TEM-1 were washed with 10 column volumes of wash buffer (50 mM KPi pH 7.8, 200 mM NaCl, 20% glycerol, 40 mM imidazole). To elute the bound protein, 5 column volumes of elution buffer (50 mM KPi pH 7.8, 200 mM NaCl, 20% glycerol, 500 mM imidazole) was flowed through the column while collecting the flow through. For downstream analysis, the protein was buffer exchanged into 50 mM KPi pH 7.8, 200 mM NaCl using the HisTrap desalting column.

#### Expression and purification of CTX-M-15

CTX-M-15 (Uniprot ID: Q9EXV5) was synthesised using the protocol described above in the expression and purification of TEM-1, with the following deviations: the gene coding for NDM-1 was synthesised as a gBlock (IDT). Following induction with l-arabinose, 1 L cultures were incubated for 20 h at 20 °C.

#### Expression and purification of NDM-1

NDM-1 (Uniprot ID: C7C422, residues G29-R270) was synthesised using the protocol described above in the expression and purification of TEM-1, with the following deviations: the gene coding for NDM-1 was synthesised as a gBlock (IDT). Following induction with l-arabinose, 1 L cultures were incubated for 20 h at 20 °C.

#### Enzyme kinetics

The rate of β-lactamase-mediated hydrolysis was montiored using a previously reported absorbance assay.^19^ In a 96-well plate, 200 μL of the test compounds at the desired concentrations with the relevant β-lactamases were incubated and the absorbance at 260 nm was monitored. Experiments using NDM-1 included 1 equivalent of ZnCl_2_. All studies were carried out using one batch of purified enzyme. Experiments were carried out at 37 °C and measurements were taken every 60 s (Epoch 2 Microplate Spectrophotometer, BioTek) for 1 h. Each sample was carried out in triplicate and the average was used for further calculations. *k*_cat_/*K*_m_ was determined from *v* = (*k*_cat_/*K*_m_)[E][S]. The initial velocity for each compound was determined and used as “*v*”.

#### MIC growth assay

Stock solutions of each compound were prepared in 50% DMSO/water at ×100 the final concentration. In a 96-well plate, 2 μL of the test compounds were added to each well. Each plate included the positive control (750 μg mL^−1^ chloramphenicol), the negative control (50% DMSO/water) and media only wells. To each test well was then added 198 μL of the required bacteria stock (OD 0.05). *E. coli* assays were carried out using LB broth, *S. aureus* were carried out using tryptic soy broth (TSB). Plates were incubated at 37 °C and the OD_600_ was measured (Epoch 2 Microplate Spectrophotometer, BioTek) every 30 min over a 17 h period. Tests were carried out in triplicate, the background absorbance was removed using reference wells, and the averages were determined. Dose curves were plotting using the OD_600_ after 16 h growth *vs.* compound concentration. MICs were defined as the minimum concentration of compound at which no significant growth was observed after 16 h incubation. MIC values were determined using GraphPad Prism (version 8.3.0).

## Funding

This work was funded by EPSRC (EP/P02324X/1).

## Conflicts of interest

There are no conflicts to declare.

## Supplementary Material

RA-010-D0RA04893C-s001
