# Supplementary material for: Synthesis and biochemical evaluation of cephalosporin analogues equipped with chemical tethers
Source: RSC Adv. 2020 Oct 2;10(60):36485–94. doi: 10.1039/d0ra04893c (PMC9056950; doi:10.1039/d0ra04893c)
Supplement: RA-010-D0RA04893C-s001 [file RA-010-D0RA04893C-s001.pdf]

Electronic Supplementary Material (ESI) for RSC Advances.
This journal is © The Royal Society of Chemistry 2020

Supporting Information

Synthesis and Biochemical Evaluation of Cephalosporin Analogues Equipped with Chemical Tethers

Lisa M. Miller,*‡ Reyme Herman,§ Ivan S. Gyulev,§ Thomas F. Krauss,⊥ Gavin H. Thomas,§ and Anne-Kathrin Duhme-Klair‡

‡Department of Chemistry, University of York, Heslington, YO10 5DD.

§Department of Biology, University of York, Heslington, YO10 5DD.

⊥Department of Physics, University of York, Heslington, YO10 5DD.

*lisa.miller@york.ac.uk

Table of Contents

S1. Compound Synthesis and Characterisation………………………………………… S-3

S1.1. General Experimental……………….……………………………………… S-3

S1.2. Materials……………………………………………………………………S-3

S1.3. Instrumentation…………………………………………………………… S-3

S1.4. Compound Spectra………………………………………………………… S-4

S2. PBP Thermal Shift Assay…………………………………………………….... S-18

S2.1. PBP3 data…………………………………………………………...........S-18

S2.2. PBP4 data…………………………………………………………...........S-20

S3. MIC/MBC_{50} Assay Results……………………………………………………..S-21

S3.1. *S. aureus* MIC curves……………………………………………………. S-21

S3.2. *E. coli* MIC curves…………………………………………………….......S-25

S3.3. MBC_{50} data……………………………………………………………….. S-30

S4. Kinetics Assay Results…………………………………………………………S-31

S1. Compound Synthesis and Characterisation.

S1.1. General Experimental

Analytical thin layer chromatography (TLC) was performed with EM Science silica gel 60 F254 aluminium plates. Visualisation was carried out using a UV lamp (254 nm) and by immersion in potassium permanganate ($KMnO_4$), followed by heating using a heat gun. Organic solutions were concentrated by rotary evaporation at 40-45 °C. Purification of reaction products were generally done by flash column chromatography using Fluka Silica, pore size 60Å, 220–440 mesh, 35–75 µm.

S1.2. Materials

Unless otherwise noted, all purchased materials were used without purification. All standard solvents were purchased from Sigma Aldrich. All standard acids, bases, and drying agents were purchased from Fisher Scientific. *N*-Hydroxy succinimide (NHS) and *N*, *N*-diisopropylethylamine (DIPEA) were purchased from Acros Organics. Cephalexin monohydrate and Boc_2O were purchased from Fluorochem. Pentynoic acid, *N*,*N'*-dicyclohexylcarbodiimide (DCC), lipoic acid, glutaric anhydride, oxalyl chloride, and Et_3N were purchased from Sigma Aldrich. DMAP was purchased from TCI.

S1.3. Instrumentation

^{1}H and ^{13}C NMR spectra were recorded on a Jeol ECS 400 (400 MHz for ^{1}H, 101 MHz for ^{13}C) at ambient temperature. Chemical shifts are reported relative to residual solvent peaks and coupling constants (*J*) are given in Hertz. High-resolution ESI mass spectra were recorded on a Bruker microTOF electrospray mass spectrometer. Infrared (IR) spectra were recorded on a PerkinElmer Spectrum Two (ATIR). Analytical HPLC measurements were performed on a Shimadzu HPLC system (Prominence) equipped with a LC-20AD pump, SIL-20A autosampler, DGU-20AS degasser, CTO-20AC column oven, CBM-20A communication bus module and SPD-M20A diode array detector using a SunFire C18 column (Waters, 4.6 x 150 mm, 5 µm). Eluent gradient: 5-95% $MeCN/H_2O$ with a 0.1% formic acid modifier, over 15 minutes.

S1.4. Compound Spectra

Compound 4, (6R,7R)-3-methyl-8-oxo-7-[(2R)-2-(pent-4-ynamido)-2-phenylacetamido]-5-thia-1-azabicyclo[4.2.0]oct-2-ene-2-carboxylic acid

^{1}H NMR: Compound 4

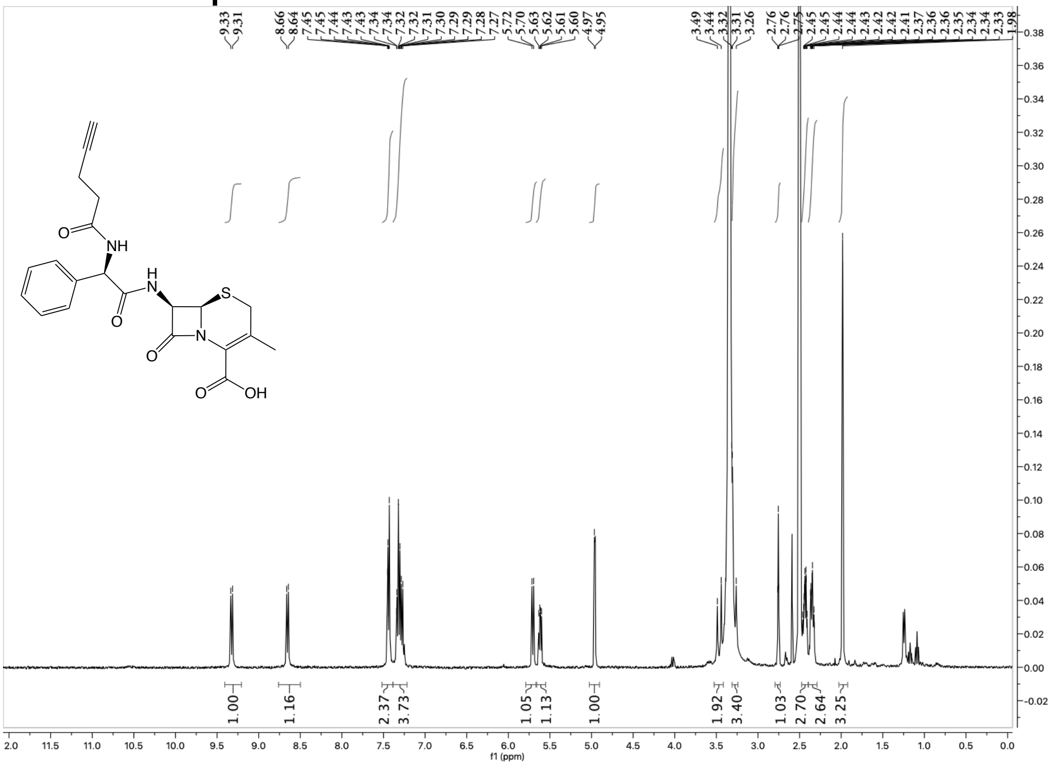

^{13}C NMR: Compound 4

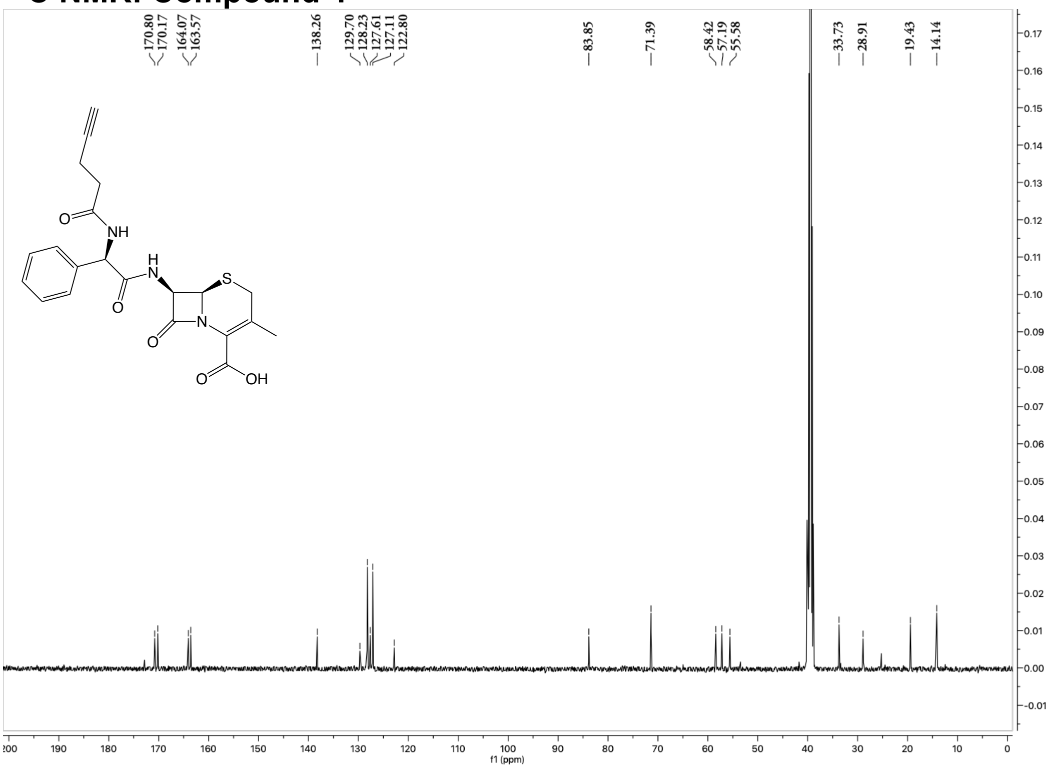

HPLC: Compound 4

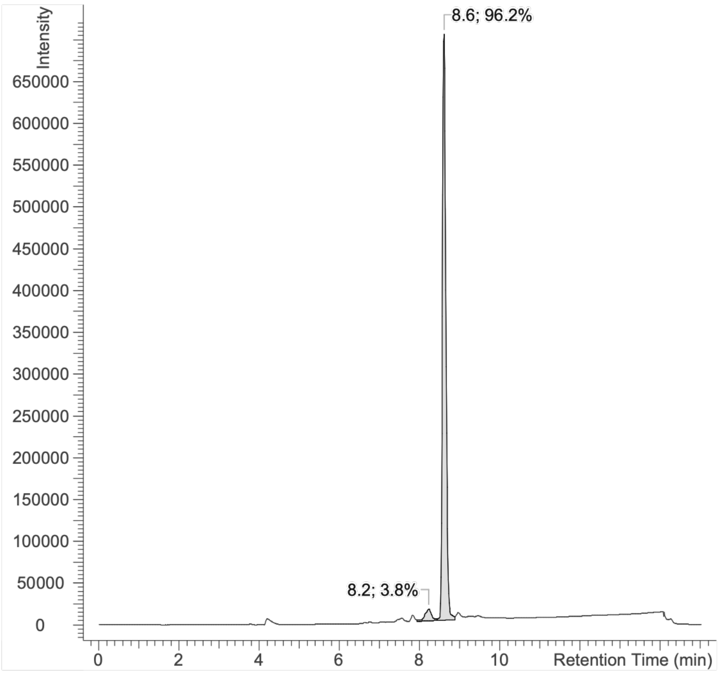

Compound 5, (6R,7R)-7-[(2R)-2-[5-(1,2-dithiolan-3-yl)pentanamido]-2-phenylacetamido]-3-methyl-8-oxo-5-thia-1-azabicyclo[4.2.0]oct-2-ene-2-carboxylic acid

^{1}H NMR: Compound 5

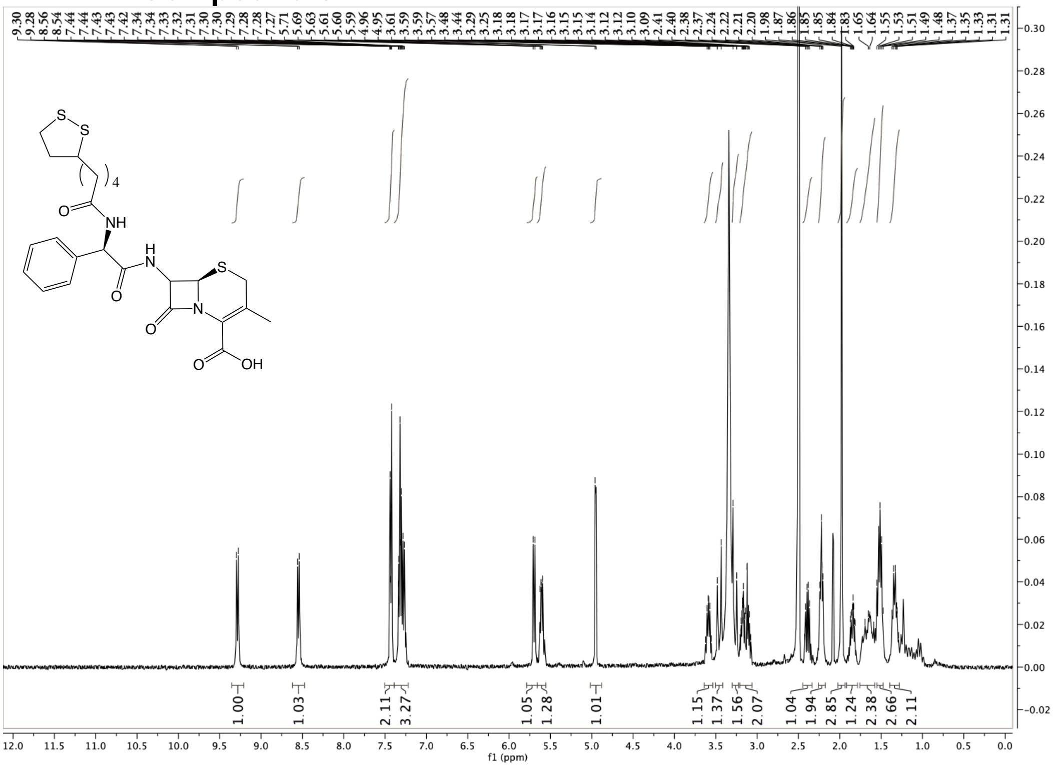

^{13}C NMR: Compound 5

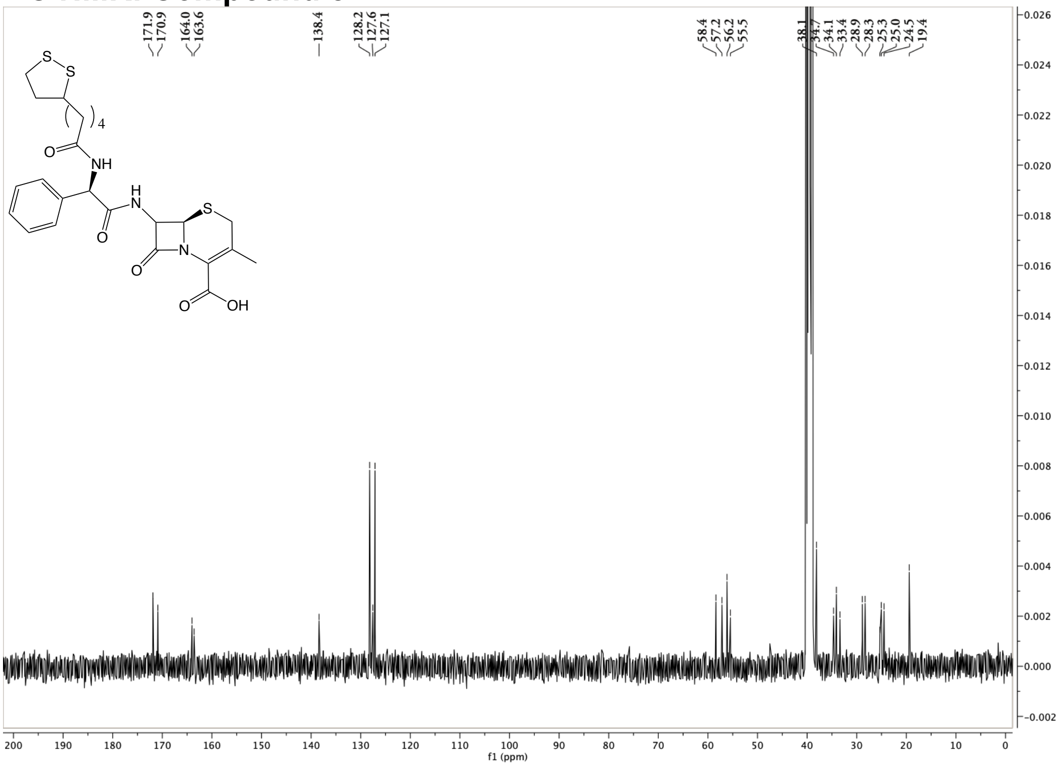

HPLC: Compound 5

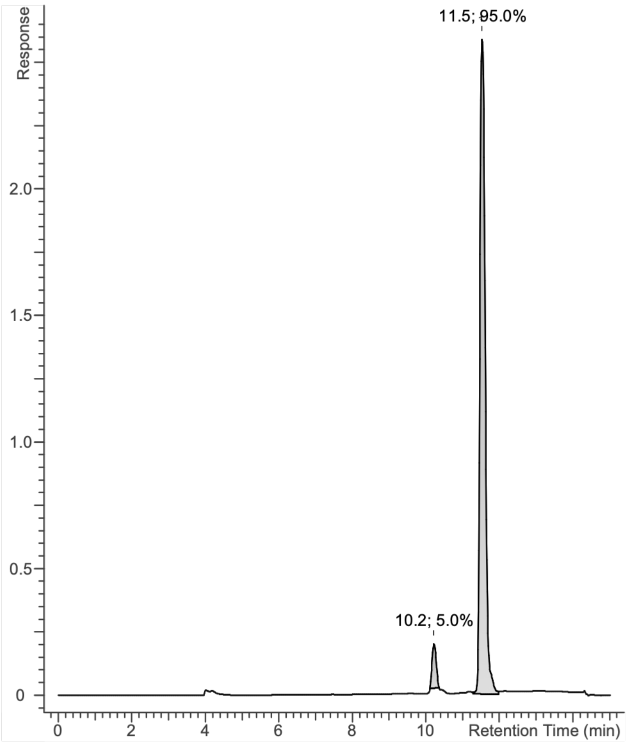

Compound 6, (6R,7R)-7-[(2R)-2-{5-[(2,5-dioxopyrrolidin-1-yl)oxy]-5-oxopentanamido}-2-phenylacetamido]-3-methyl-8-oxo-5-thia-1-azabicyclo[4.2.0]oct-2-ene-2-carboxylic acid

^{1}H NMR: Compound 6

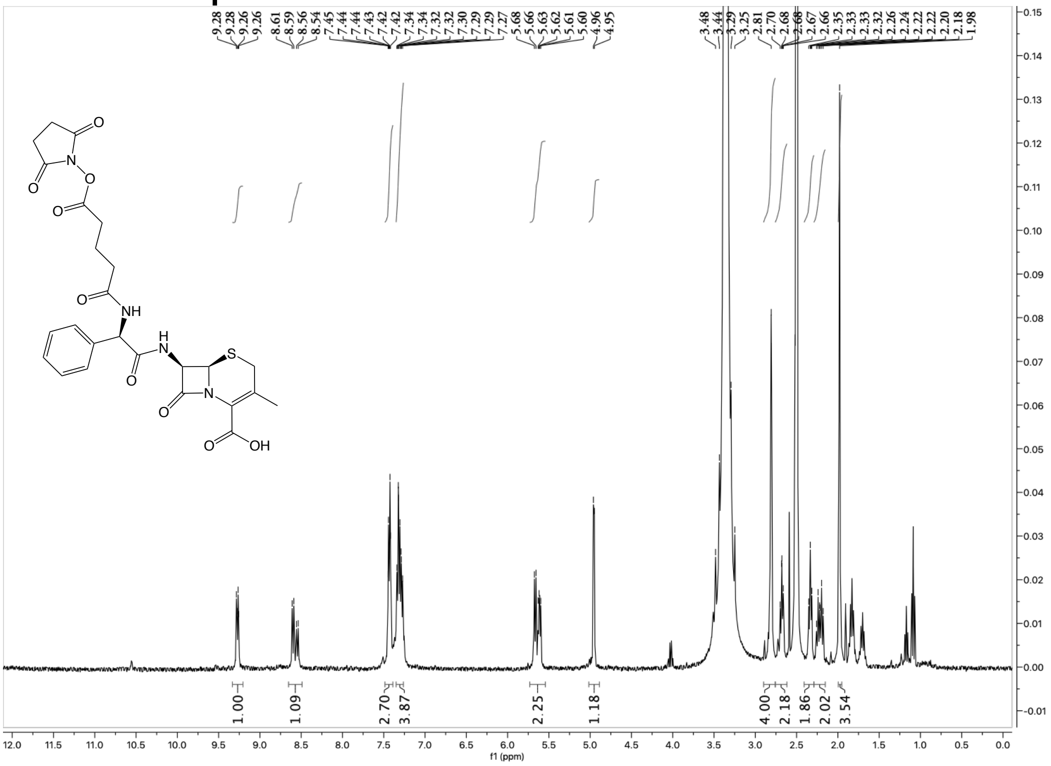

^{13}C NMR: Compound 6

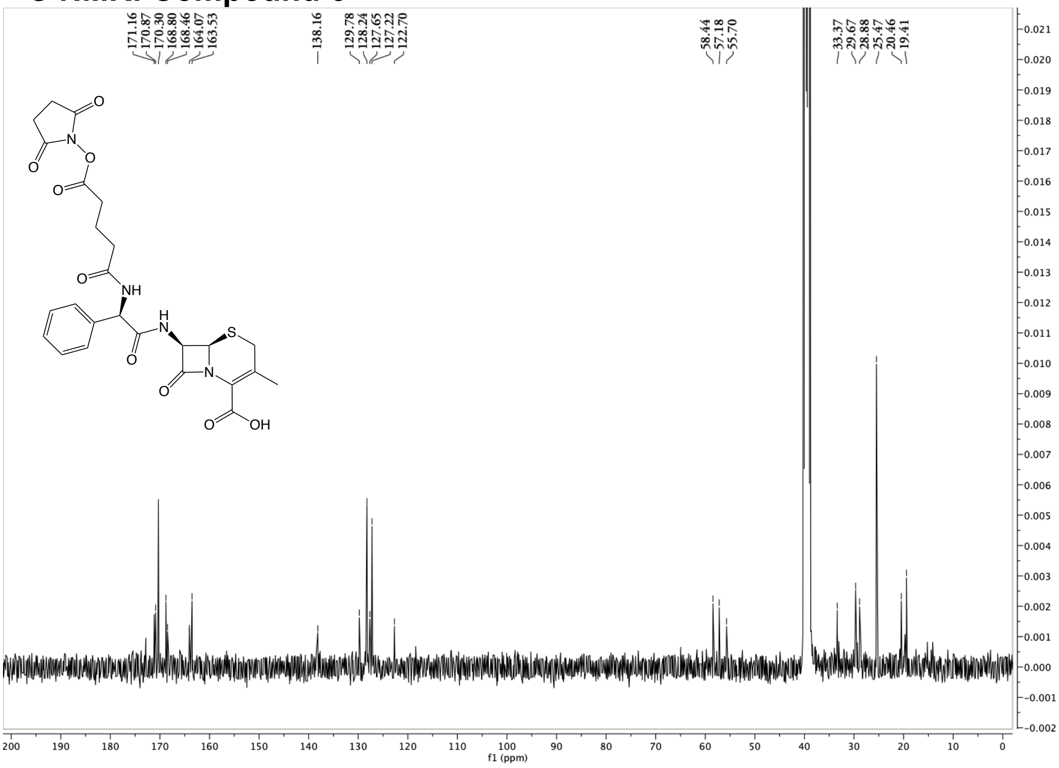

QNMR: Compound 6, ^{1}H NMR in DMSO-d_6 with maleic acid reference

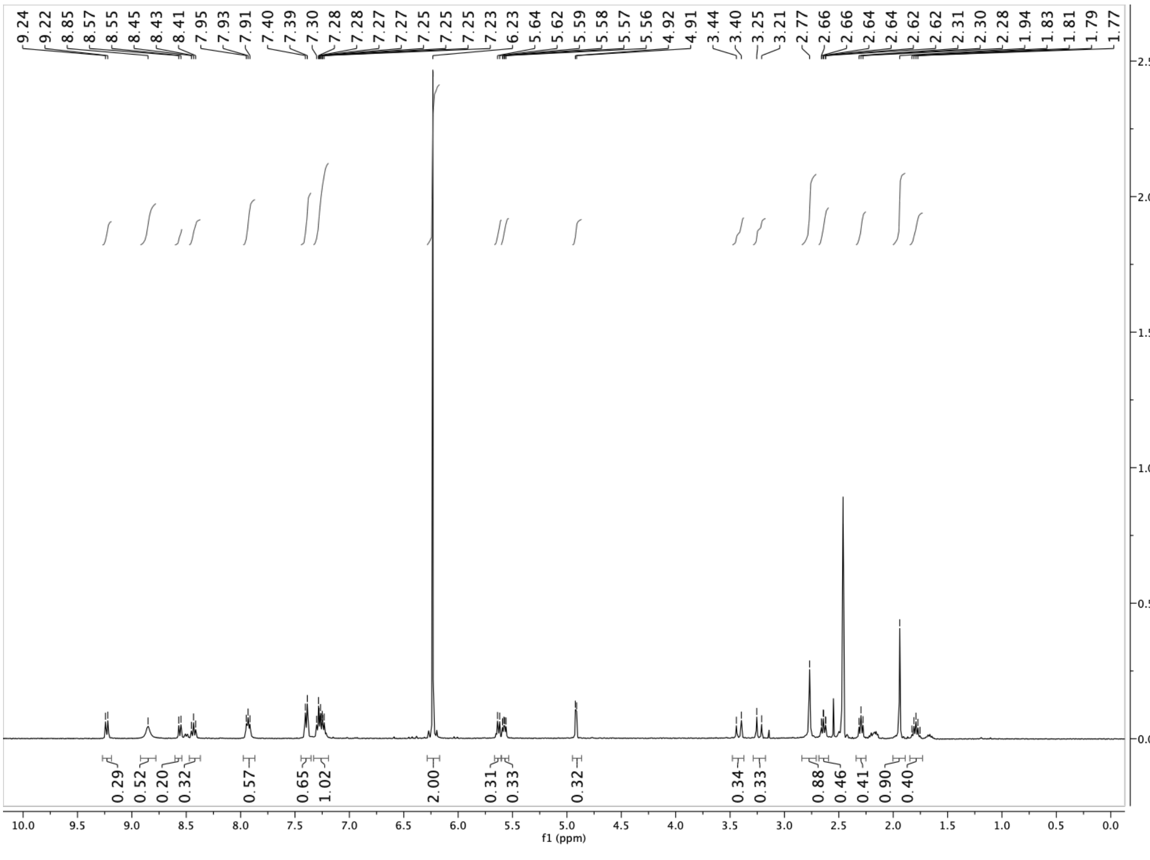

QNMR purity determination:

Reference: Maleic acid, MW 116, mass 2.4 mg, δ 7.23 (s, 2H)

Sample: Compound 6, MW 558, mass 4.1 mg, average of three peaks = 0.32 (δ 5.63 (d, 0.31H), 5.58 (dd, 0.33H), 4.91 (d, 0.32H))

%purity = (measured mmol/predicted mmol) x 100
= (0.0066/0.0073) x 100
= 90.4%

Compound 7, (6R,7R)-7-[(2R)-2-(4-carboxybutanamido)-2-phenylacetamido]-3-methyl-8-oxo-5-thia-1-azabicyclo[4.2.0]oct-2-ene-2-carboxylic acid

^{1}H NMR: Compound 7

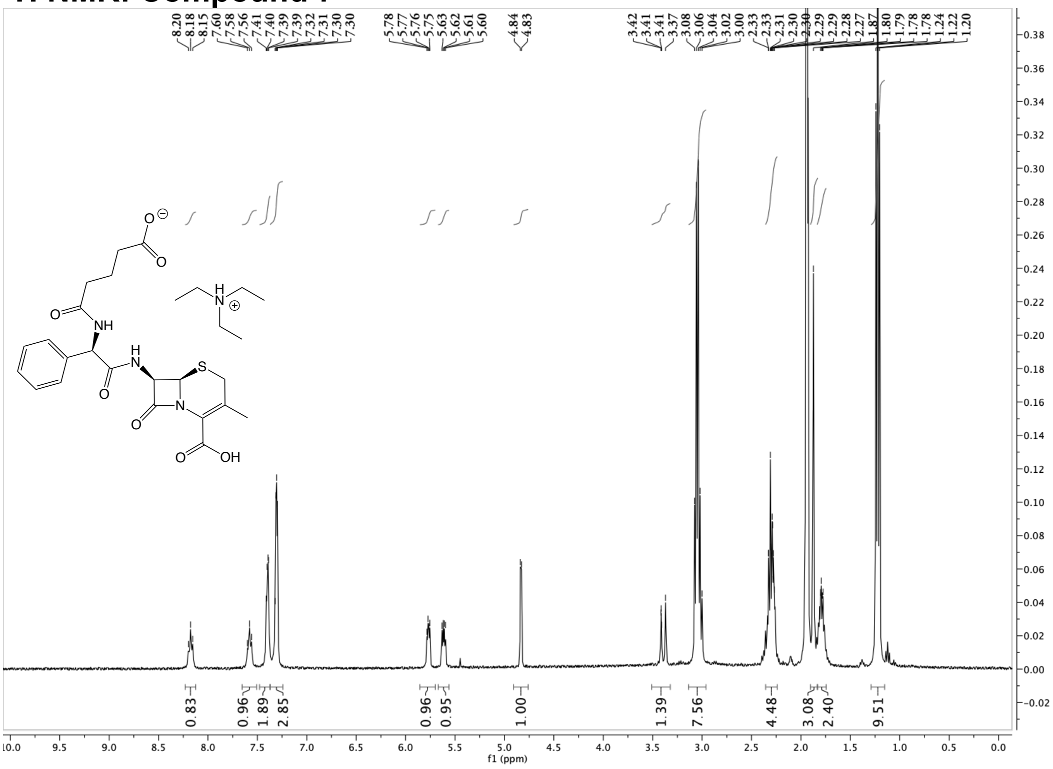

^{13}C NMR: Compound 7

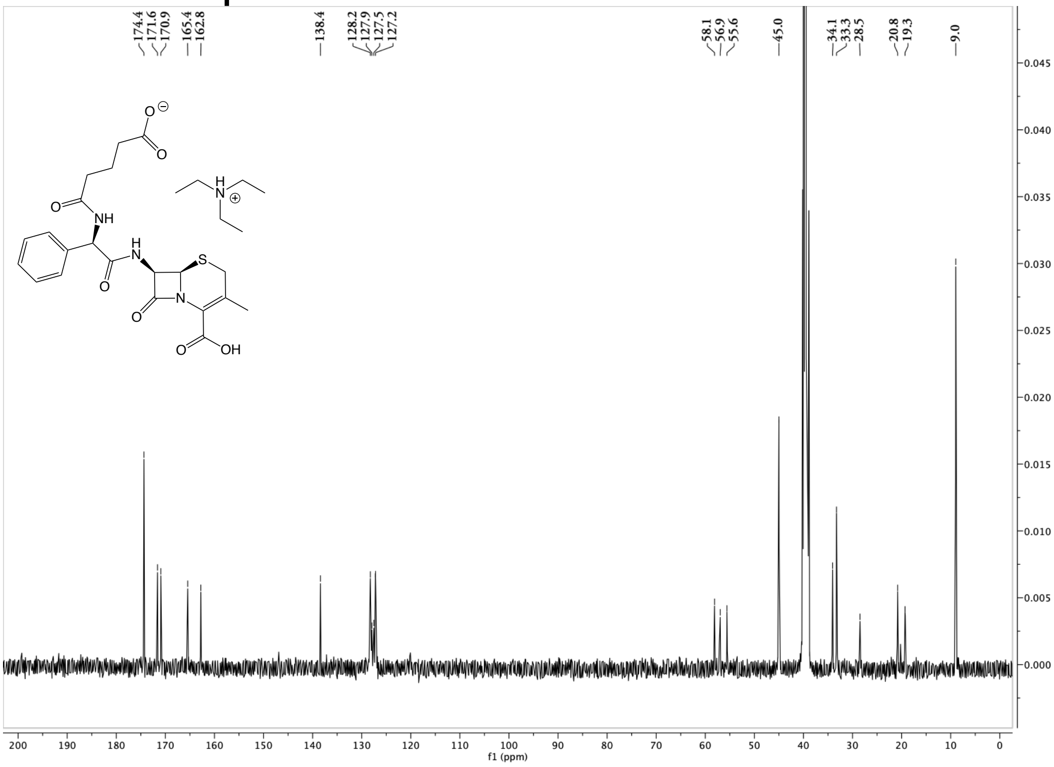

HPLC: Compound 7

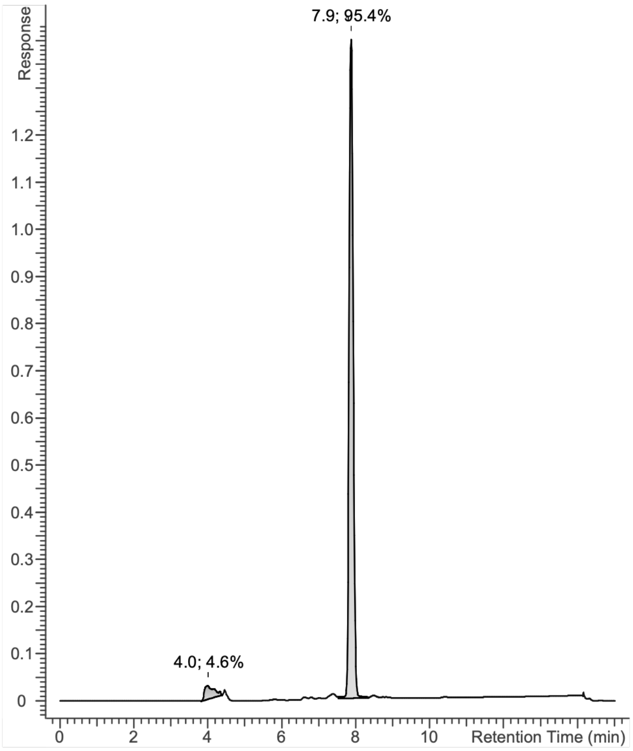

Compound 8, (6R,7R)-7-[(2R)-2-acetamido-2-phenylacetamido]-3-methyl-8-oxo-5-thia-1-azabicyclo[4.2.0]oct-2-ene-2-carboxylic acid

^{1}H NMR: Compound 8

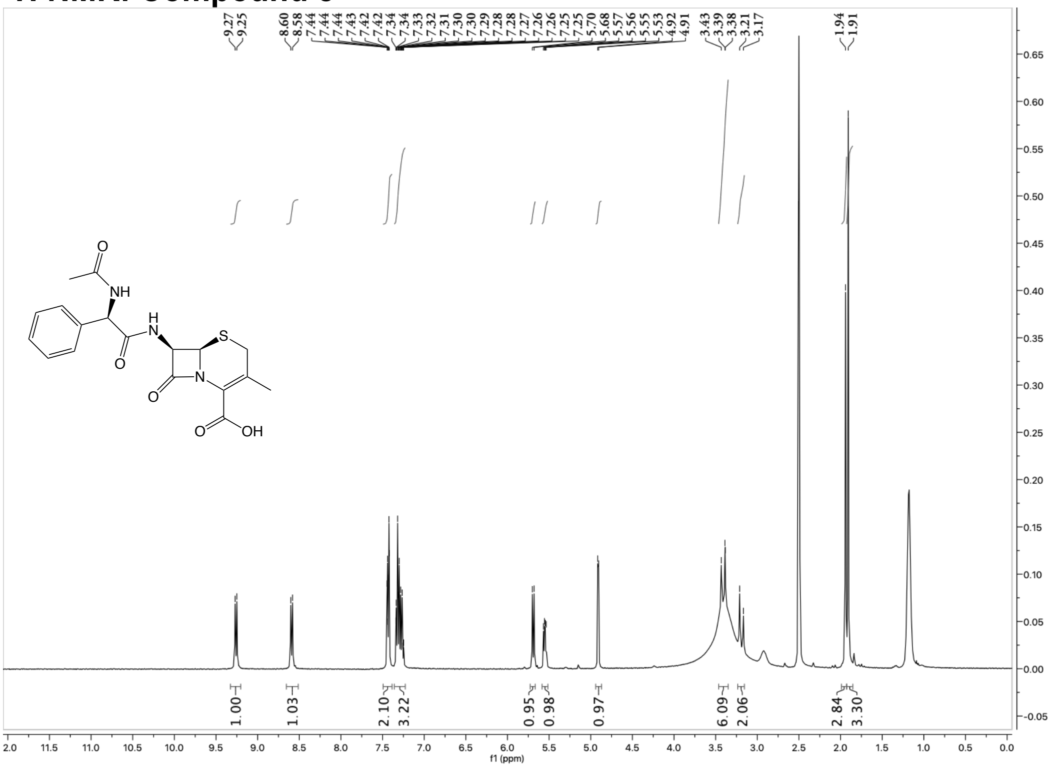

^{13}C NMR: Compound 8

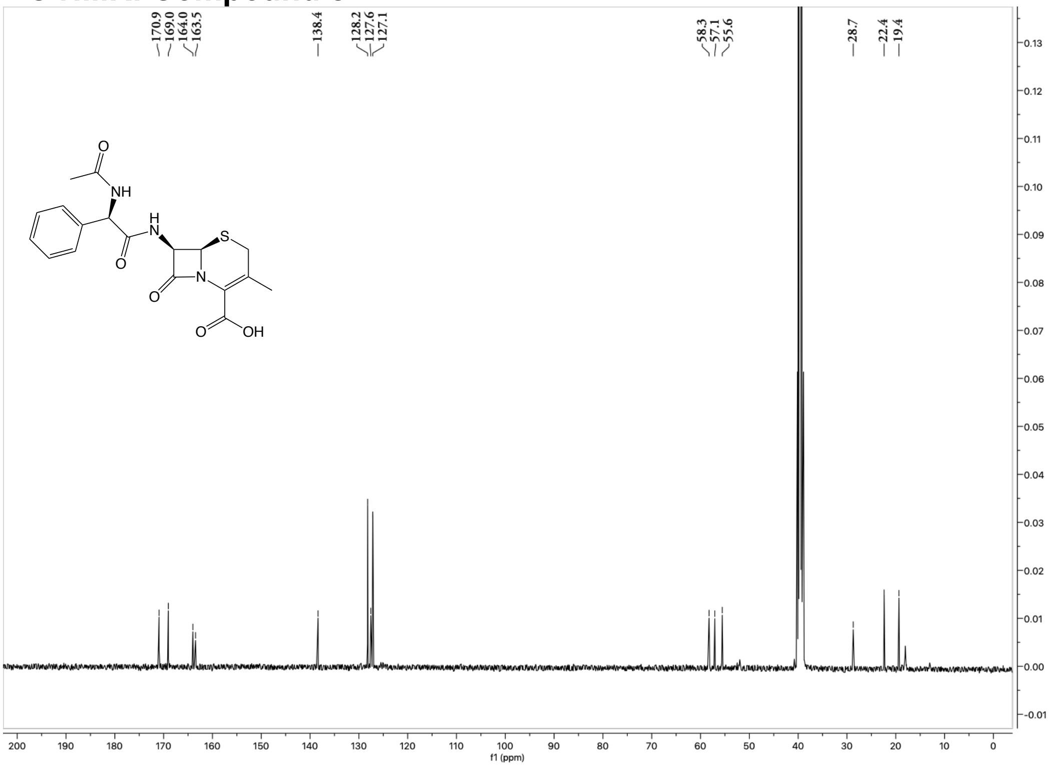

HPLC: Compound 8

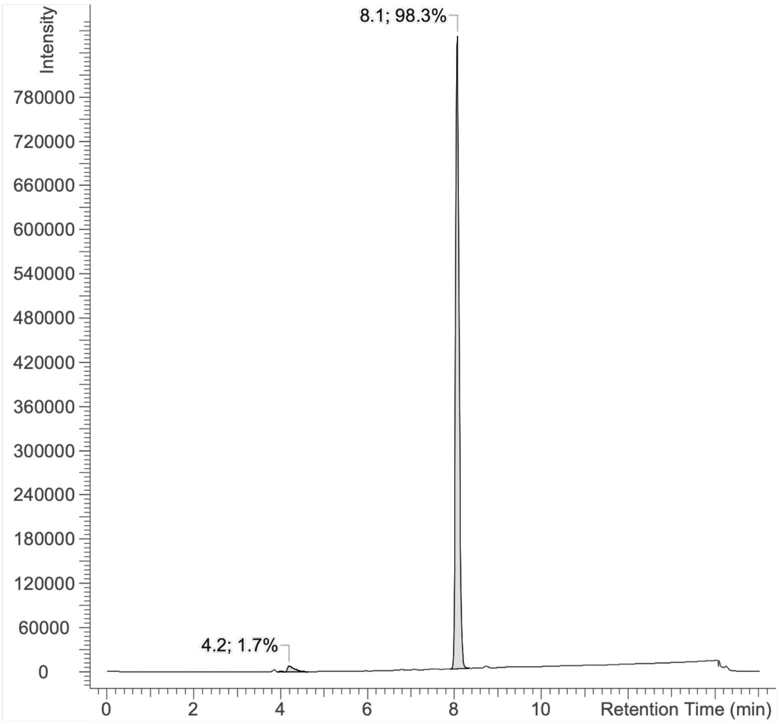

Compound 9, (6R,7R)-7-[(2R)-2-{[(tert-butoxy)carbonyl]amino}-2-phenylacetamido]-3-methyl-8-oxo-5-thia-1-azabicyclo[4.2.0]oct-2-ene-2-carboxylic acid

^{1}H NMR: Compound 9

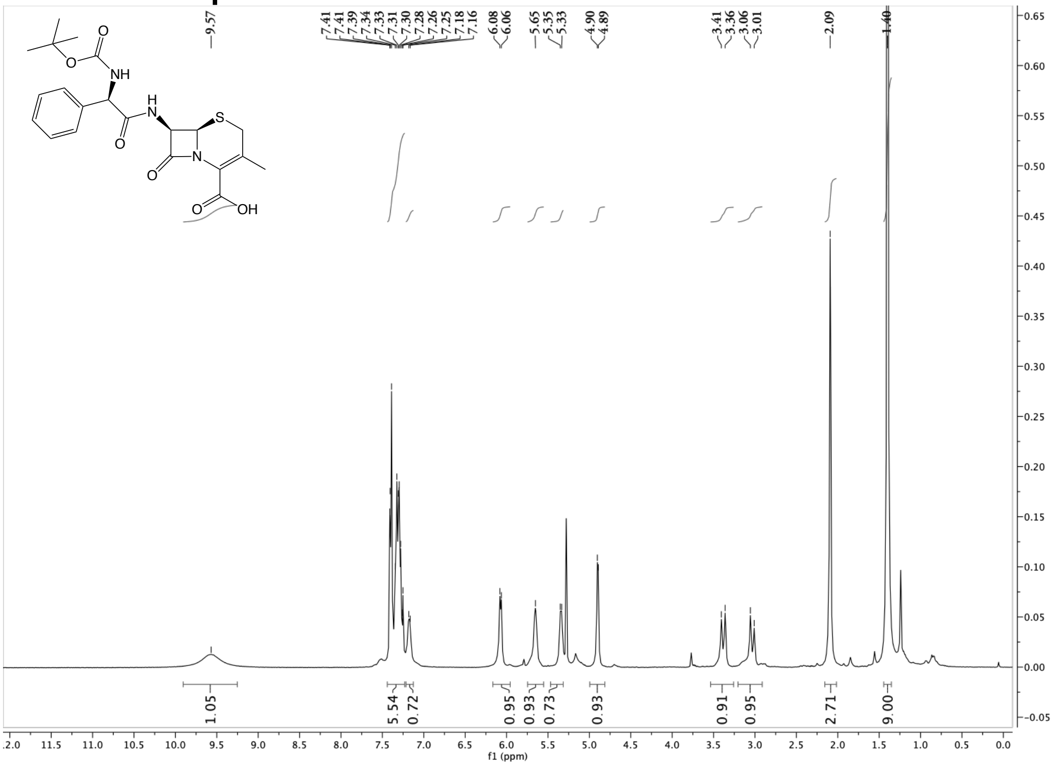

^{13}C NMR: Compound 9

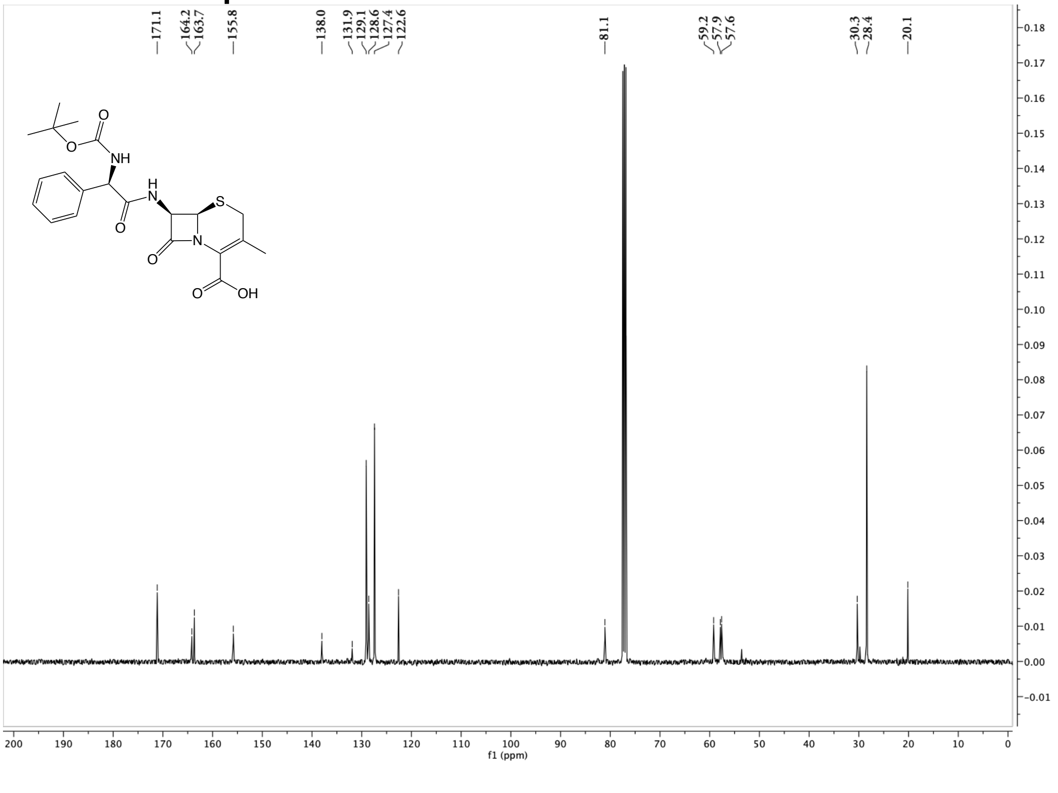

HPLC: Compound 9

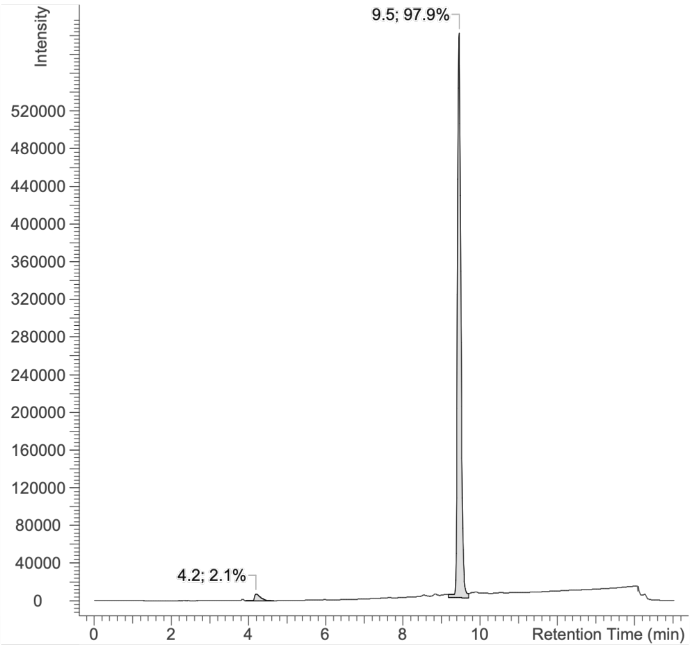

Compound 10, (6R,7R)-7-[(2R)-2-(5-aminopentanamido)-2-phenylacetamido]-3-methyl-8-oxo-5-thia-1-azabicyclo[4.2.0]oct-2-ene-2-carboxylic acid

^{1}H NMR: Compound 10

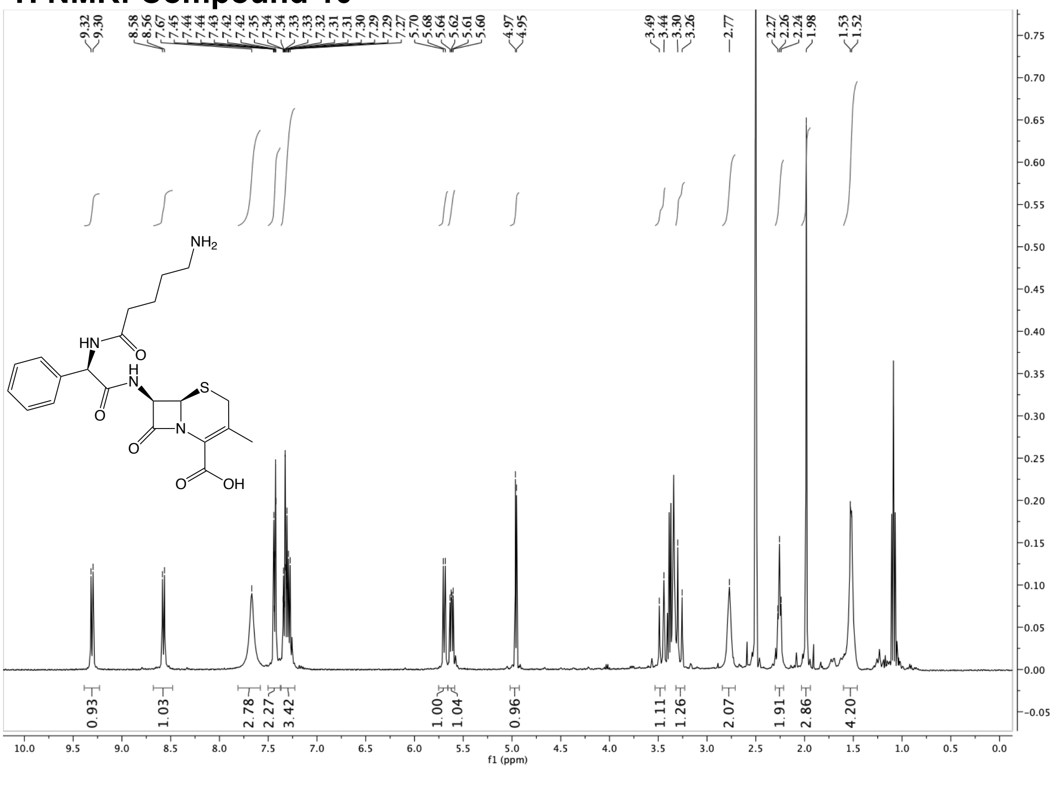

^{13}C NMR: Compound 10

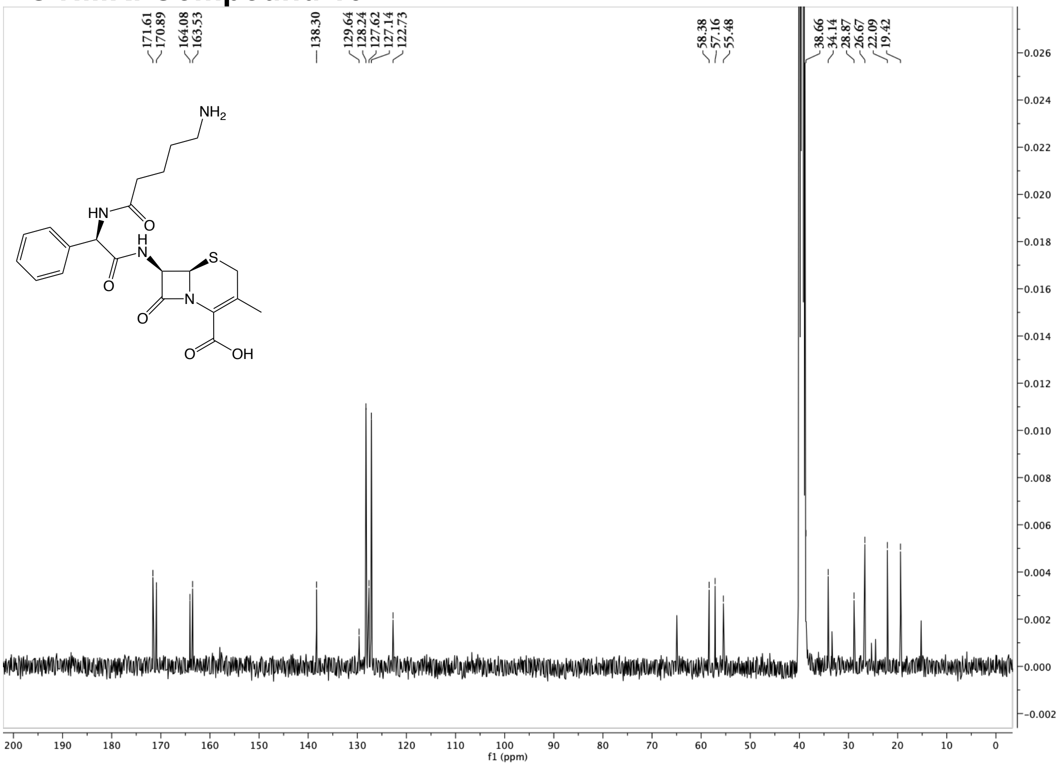

HPLC: Compound 10

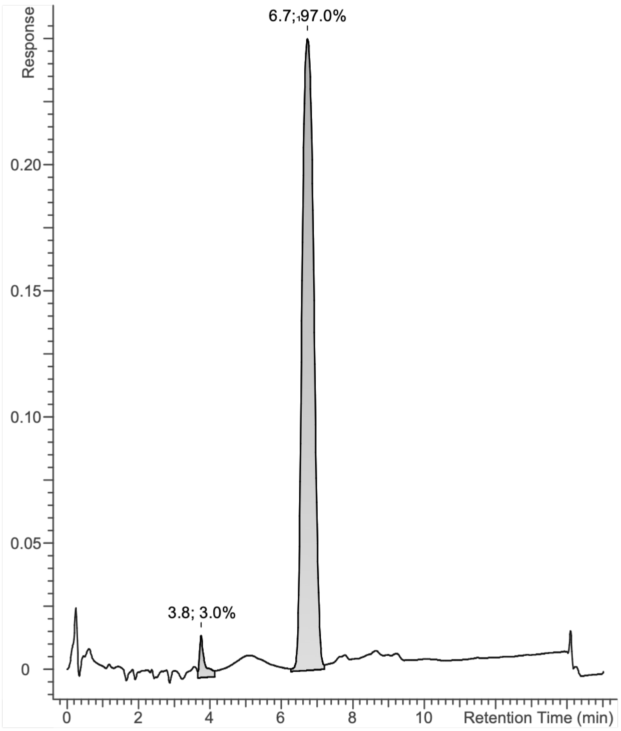

Compound 18, 2,5-dioxopyrrolidin-1-yl 5-(1,2-dithiolan-3-yl)pentanoate

^{1}H NMR: Compound 18

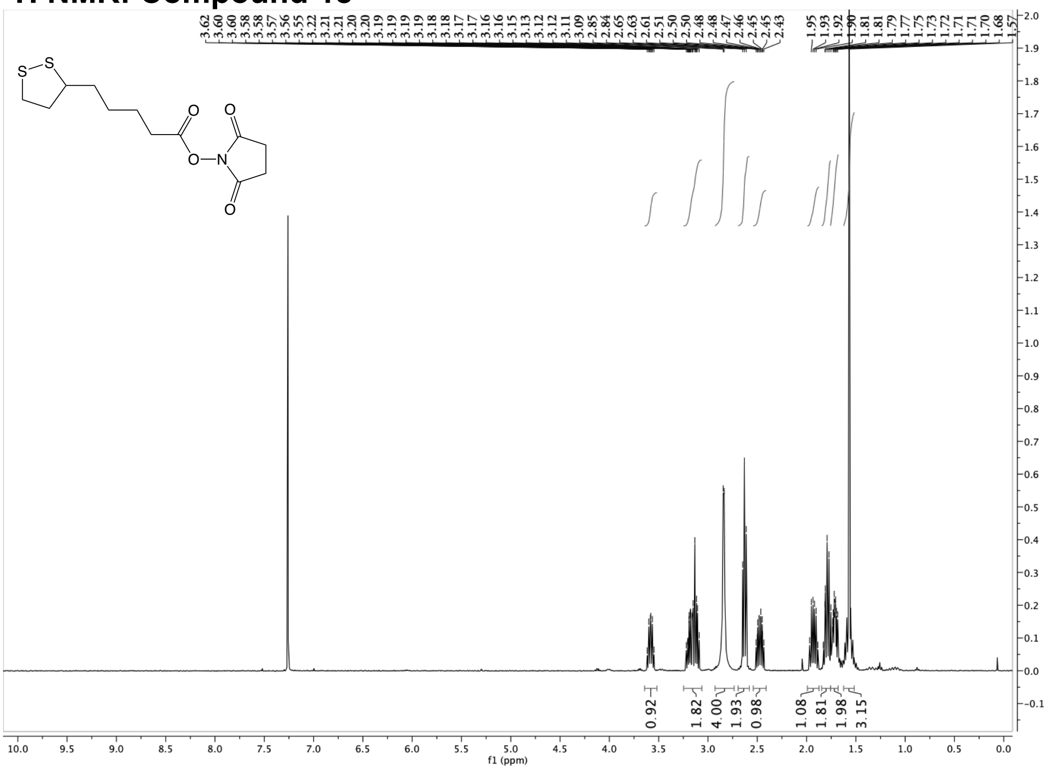

^{13}C NMR: Compound 18

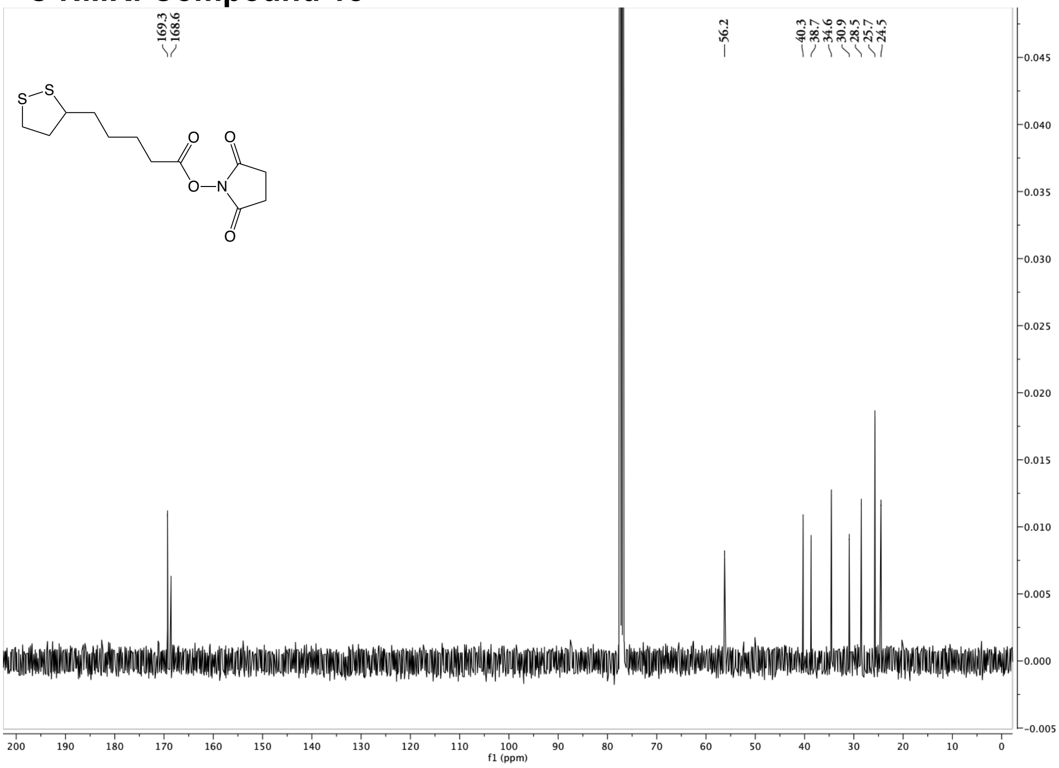

Compound 22, 2,5-dioxopyrrolidin-1-yl 5-{[(*tert*-butoxy)carbonyl]amino} pentanoate

^{1}H NMR: Compound 22

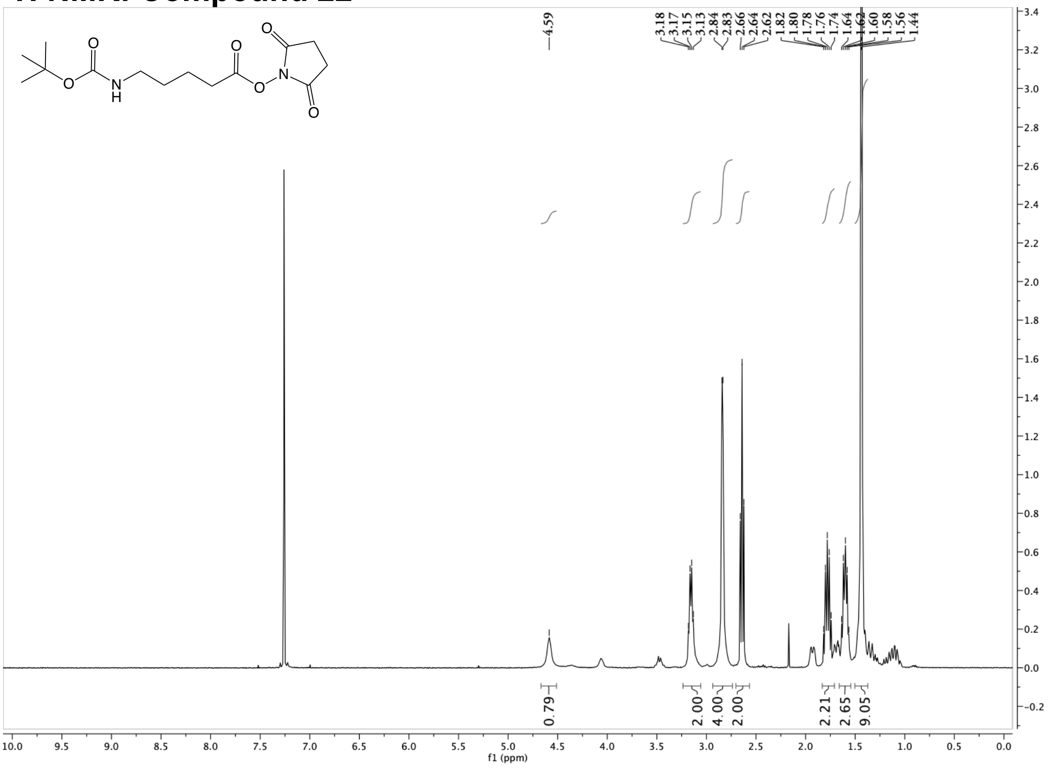

^{13}C NMR: Compound 22

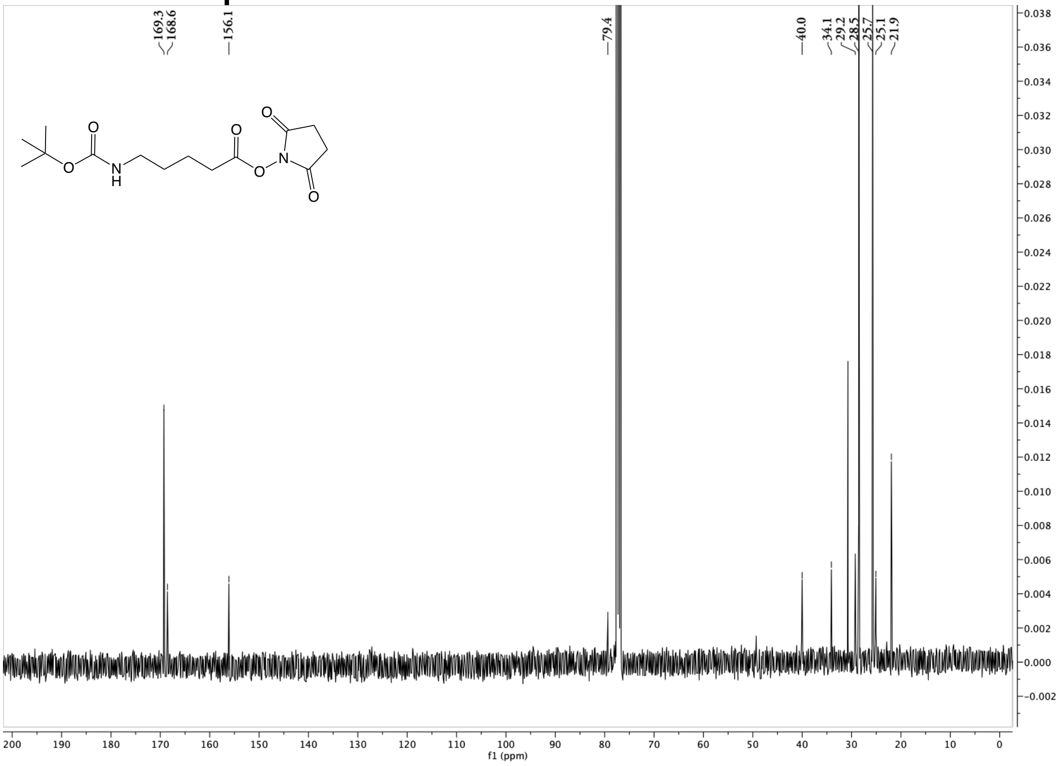

Compound 23, (6R,7R)-7-[(2R)-2-(5-{[(*tert*-butoxy)carbonyl]amino}pentanamido)-2-phenylacetamido]-3-methyl-8-oxo-5-thia-1-azabicyclo[4.2.0]oct-2-ene-2-carboxylic acid

^{1}H NMR: Compound 23

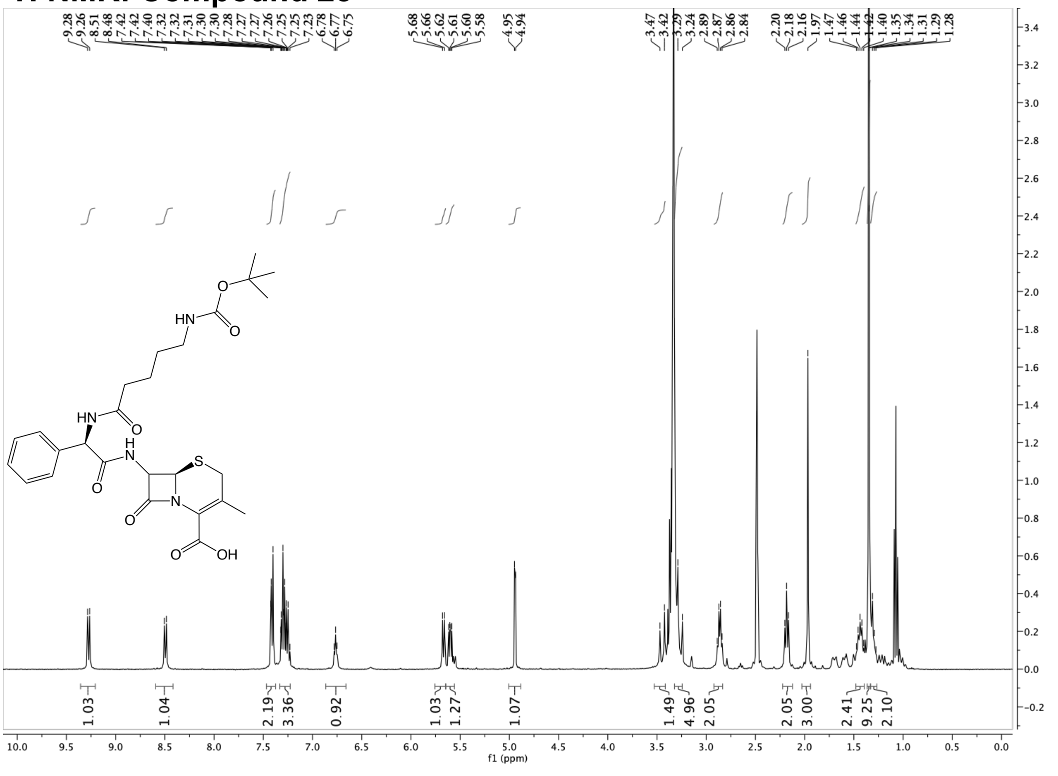

^{13}C NMR: Compound 23

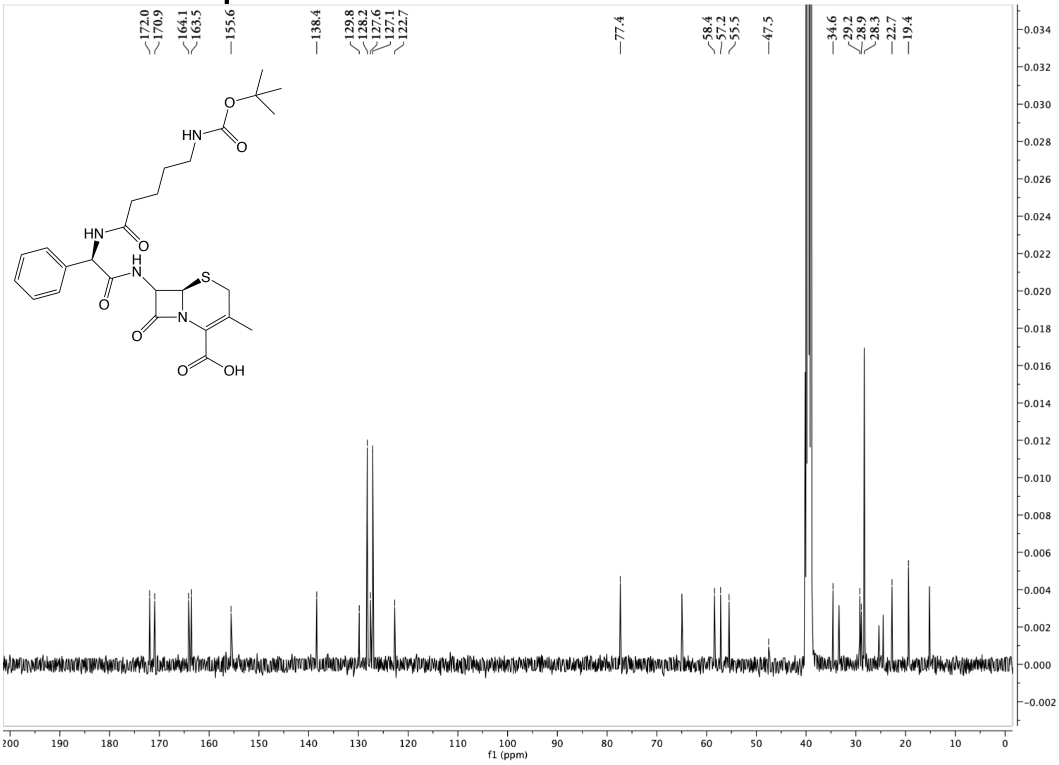

S2. PBP Thermal Shift Assay

S2.1. PBP3 Data

Table S1: Thermal shift assay results with PBP3

Sample	Tm 1/°C	Tm 2/°C	Tm 3/°C	Average Tm/°C	
**PBP3**	55.2570138	55.2570138	55.2570138	55.2570138	
	**ΔTm 1/°C**	**ΔTm 2/°C**	**ΔTm 3/°C**	**Average ΔTm/°C**	**ΔTm/°C, ±s.d.**
**Compound 1**	+1.8065	+1.8065	+1.2076	+1.6069	+1.61, ±0.35
**Compound 2**	+4.7994	+4.7994	+4.5585	+4.7191	+4.51, ±0.14
**Compound 3**	+3.5988	+3.5988	+1.8065	+3.0014	+3.00, ±1.03
**Compound 4**	+3.009	+3.5988	+2.4077	+3.0052	+3.01, ±0.60
**Compound 5**	+3.009	+3.009	+3.009	+3.009	+3.01, ±0
**Compound 6**	+3.5988	+3.009	+3.009	+3.2056	+3.21, ±0.34
**Compound 7**	+3.5988	+3.5988	+3.5988	+3.5988	+3.60, ±0
**Compound 8**	+3.009	+3.009	+3.009	+3.009	+3.01, ±0
**Compound 9**	+1.8065	+1.8065	+1.2076	+1.6069	+1.61, ±0.34
**Compound 10**	+3.009	+2.4077	+3.009	+2.8086	+2.81, ±0.35
**Compound 11**	+3.5988	+3.009	+3.5988	+3.4022	+3.40, ±0.34
**Compound 12**	+4.2194	+4.816	+4.2194	+4.4182	+4.42, ±0.34

Relative Tm values vs. ligand equivalents for compounds **1-12** with PBP3. Relative Tm values were calculated as a ratio relative to the highest Tm value and plotted against the equivalents of ligand used. Values are an average of three runs.

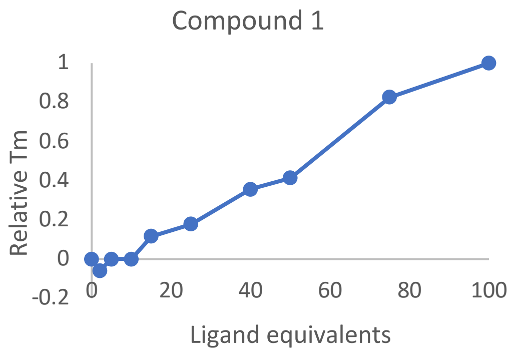

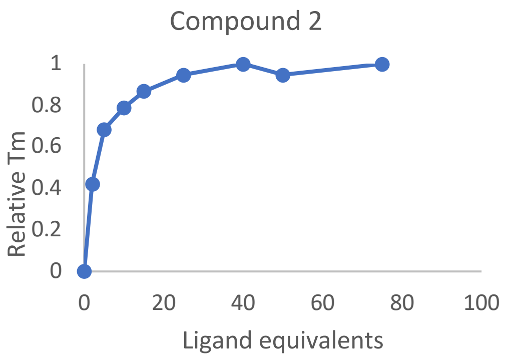

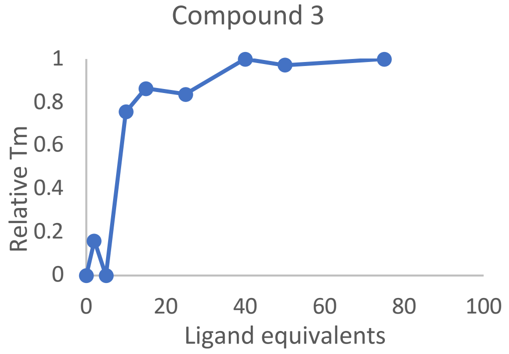

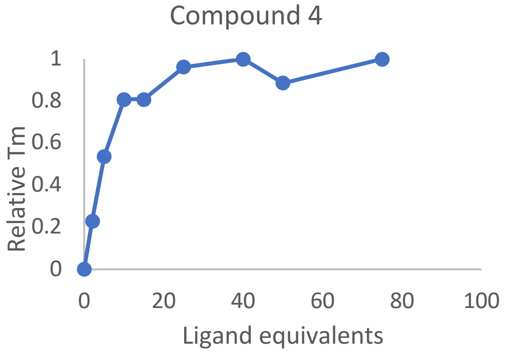

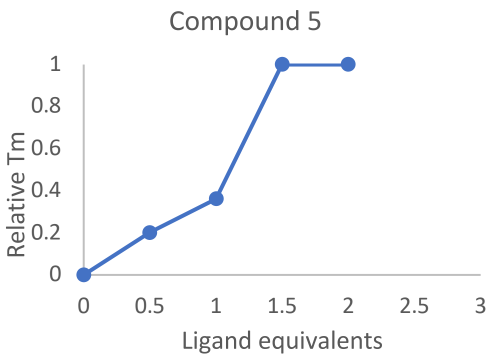
Compound 5
Relative Tm
Ligand equivalents
0
0.2
0.4
0.6
0.8
1
0
0.5
1
1.5
2
2.5
3
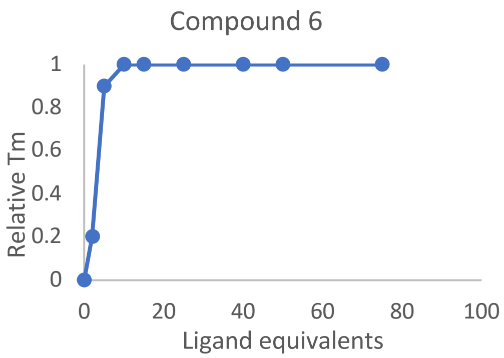
Compound 6
Relative Tm
Ligand equivalents
0
0.2
0.4
0.6
0.8
1
0
20
40
60
80
100
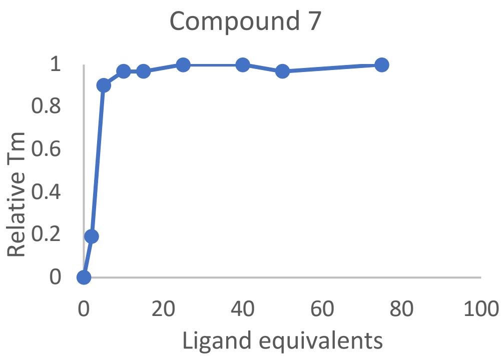
Compound 7
Relative Tm
Ligand equivalents
0
0.2
0.4
0.6
0.8
1
0
20
40
60
80
100
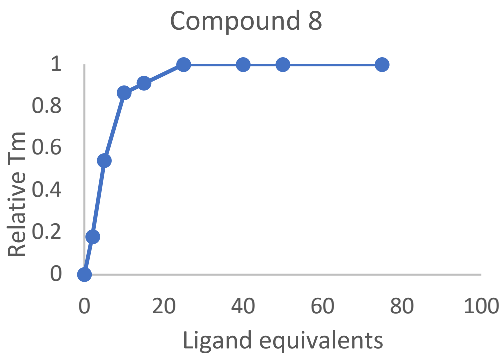
Compound 8
Relative Tm
Ligand equivalents
0
0.2
0.4
0.6
0.8
1
0
20
40
60
80
100
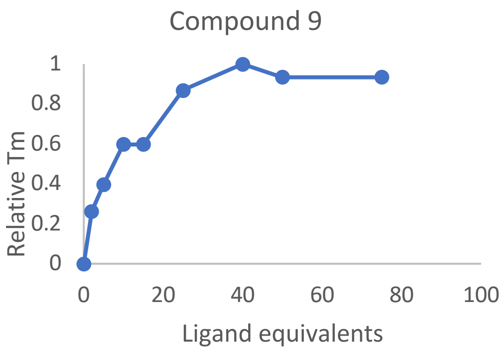
Compound 9
Relative Tm
Ligand equivalents
0
0.2
0.4
0.6
0.8
1
0
20
40
60
80
100
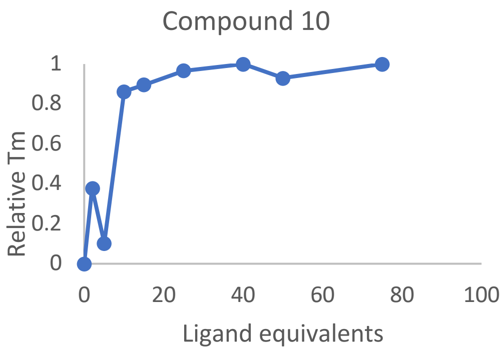
Compound 10
Relative Tm
Ligand equivalents
0
0.2
0.4
0.6
0.8
1
0
20
40
60
80
100
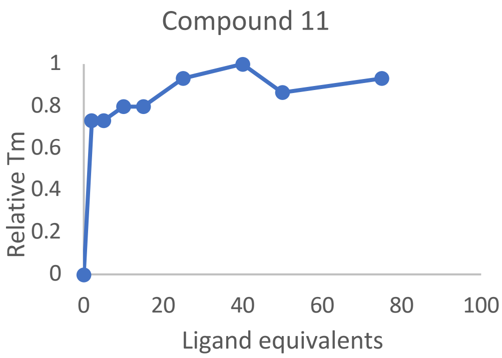
Compound 11
Relative Tm
Ligand equivalents
0
0.2
0.4
0.6
0.8
1
0
20
40
60
80
100
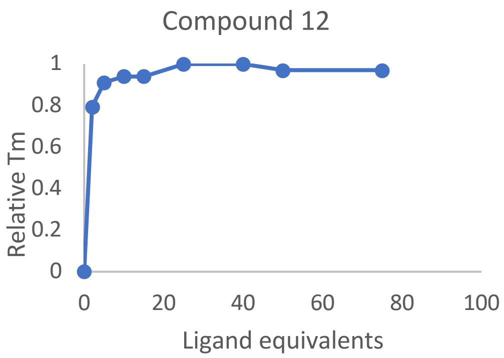
Compound 12
Relative Tm
Ligand equivalents
0
0.2
0.4
0.6
0.8
1
0
20
40
60
80
100

S2.2. PBP4 Data

Table S3: Thermal shift assay results with PBP4

Sample	Tm 1/°C	Tm 2/°C	Tm 3/°C	Average Tm/°C	
**PBP4**	54.24049	54.2415771	54.4431267	54.3083979	
	**ΔTm 1/°C**	**ΔTm 2/°C**	**ΔTm 3/°C**	**Average ΔTm/°C**	**ΔTm/°C, ±s.d.**
**Compound 1**	-1.0801036	-0.8823153	-0.6821696	-0.8815295	-0.88, ±0.20
**Compound 2**	-2.3243078	-2.1986516	-1.33793	-1.9536298	-1.95, ±0.54
**Compound 3**	-0.2168897	-0.0951398	-0.82245	-0.3781598	-0.38, ±0.39
**Compound 4**	-0.8124097	-0.6918615	-1.0553525	-0.8532079	-0.85, ±0.19
**Compound 5**	-0.0615412	-1.2663256	-0.0632731	-0.4637133	-0.46, ±0.70
**Compound 6**	-0.8645109	-0.5057653	-0.7545522	-0.7082761	-0.71, ±0.18
**Compound 7**	-0.2618154	-0.5028661	-0.502477	-0.4223862	-0.42, ±0.14
**Compound 8**	0.24304072	-0.4932111	-0.4936002	-0.2479235	-0.25, ±0.43
**Compound 9**	-0.7306188	-0.7299665	-0.9672788	-0.809288	-0.81, ±0.14
**Compound 10**	0.25836054	-0.4749921	0.13492076	-0.0272369	-0.03, ±0.39
**Compound 11**	-0.7074216	-0.5856679	-0.9453786	-0.7461561	-0.75, ±0.18
**Compound 12**	-0.6960424	-0.5755933	-0.8143819	-0.6953392	-0.70, ±0.12

S3. MIC Assay Results

S3.1. *S. aureus* MIC curves

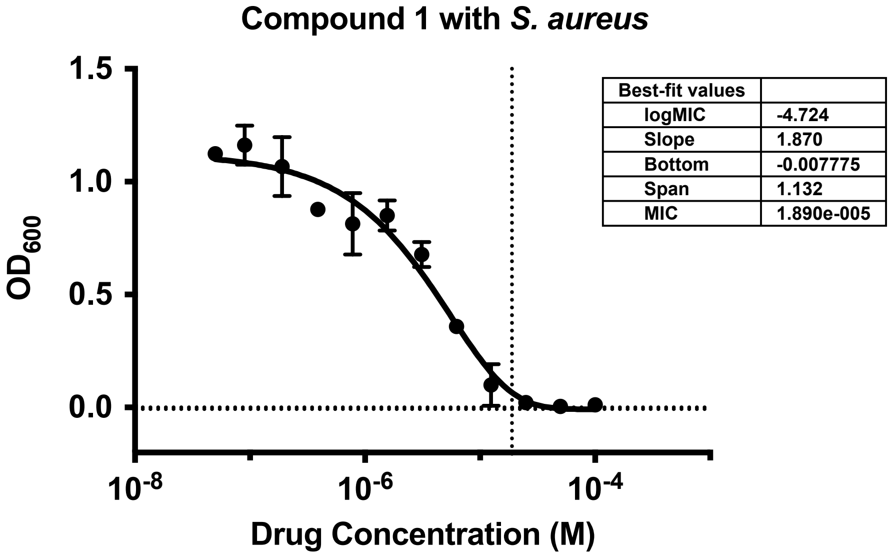

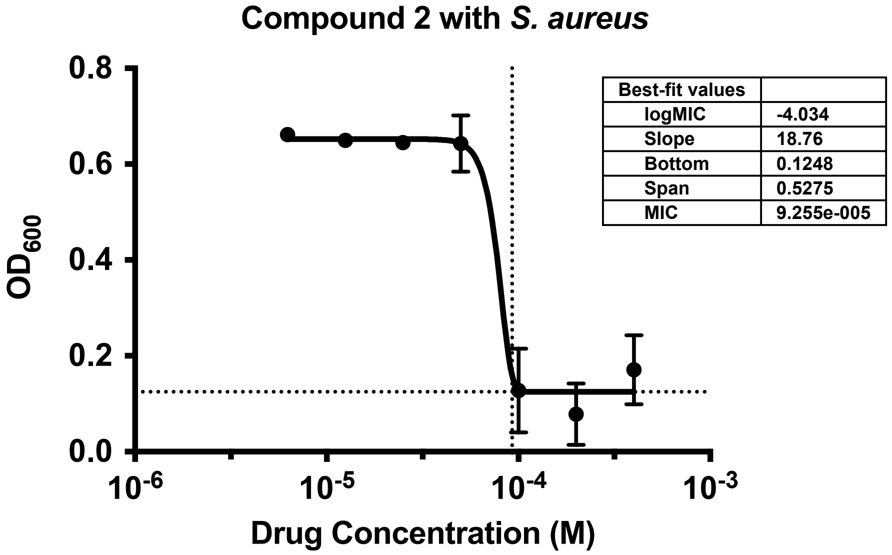

Compound 3 with *S. aureus*

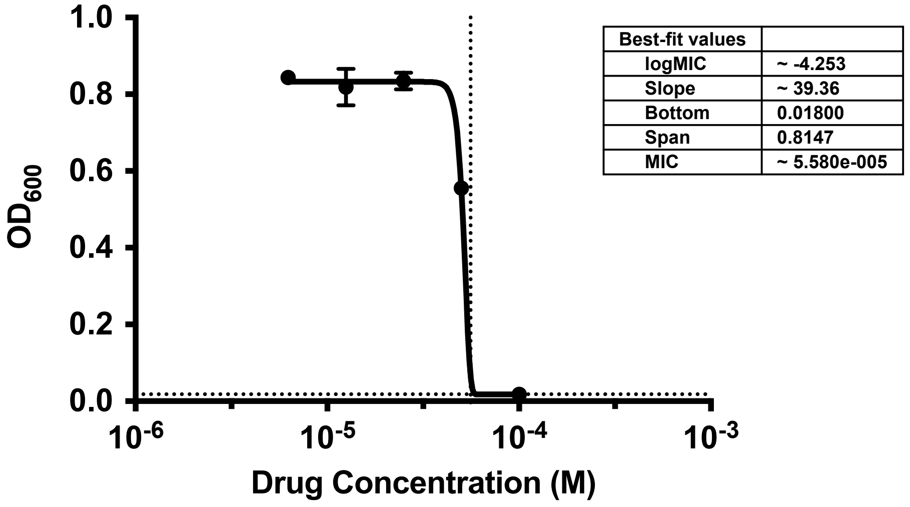

Compound 4 with *S. aureus*

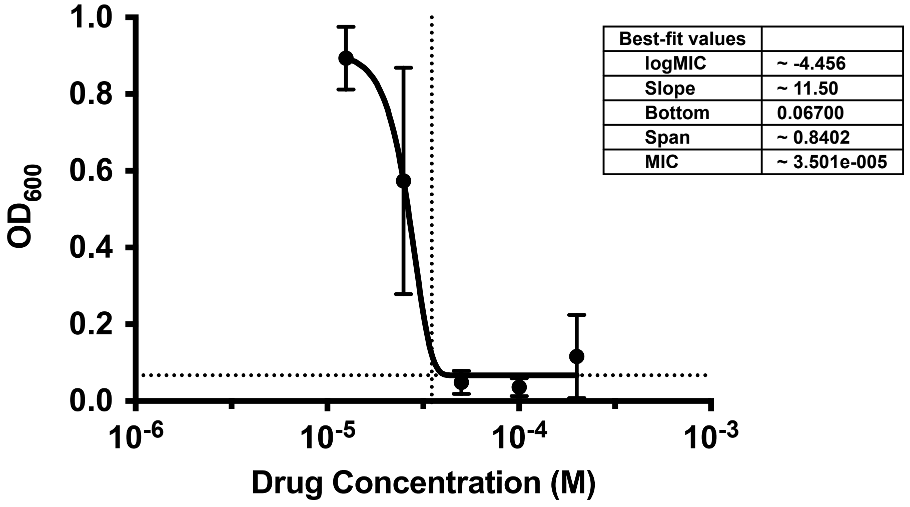

Compound 5 with *S. aureus*

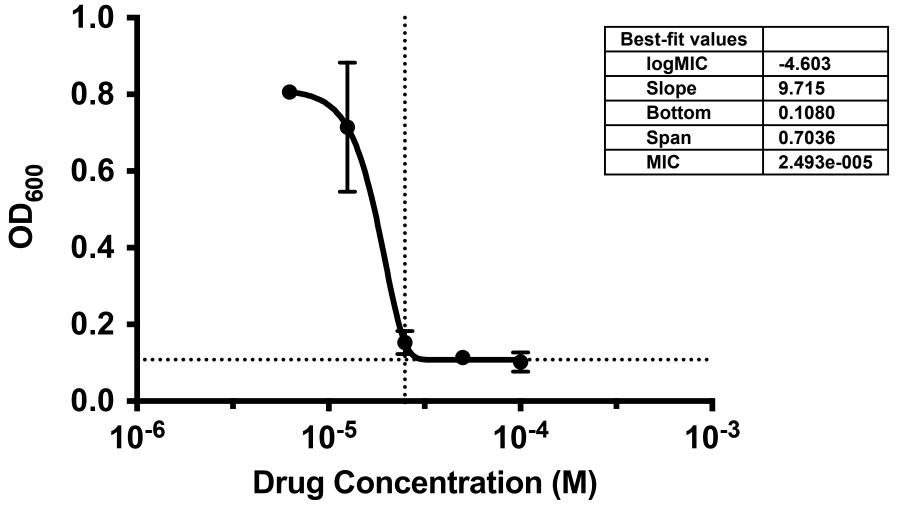

Compound 6 with *S. aureus*

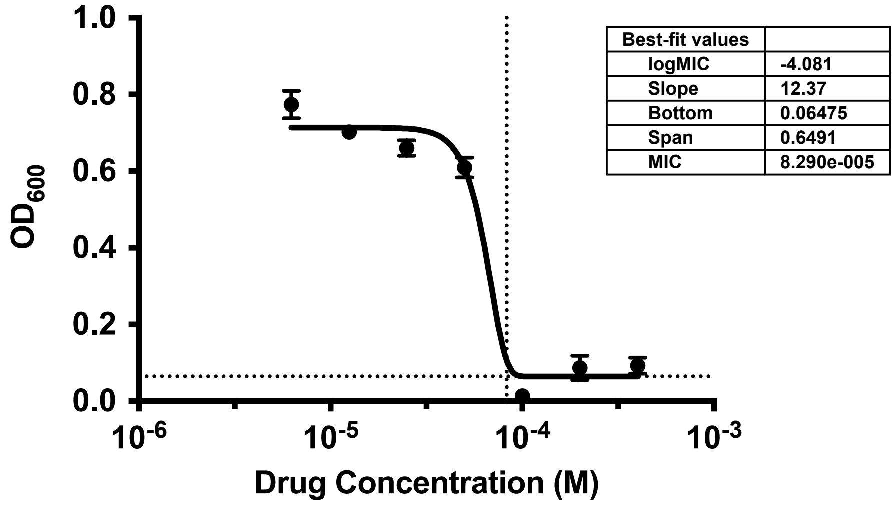

Compound 7 with *S. aureus*

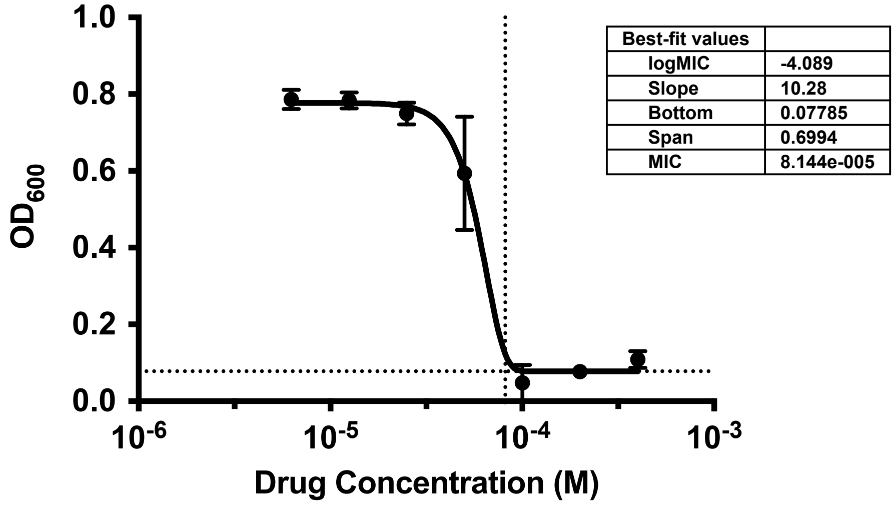

Compound 8 with *S. aureus*

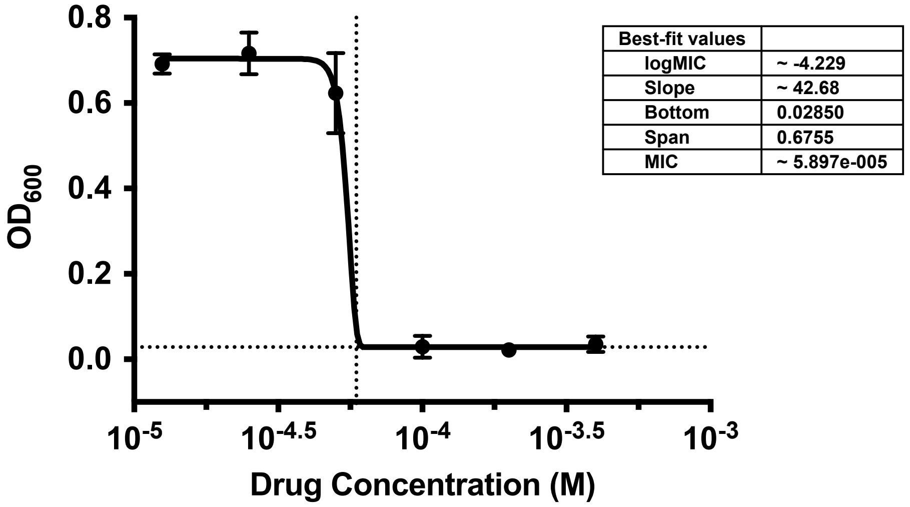

Compound 9 with *S. aureus*

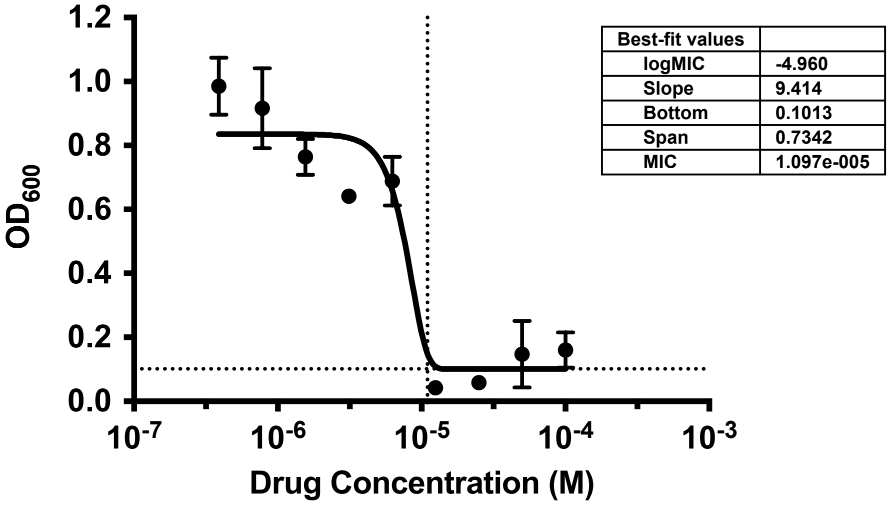

Compound 10 with *S. aureus*

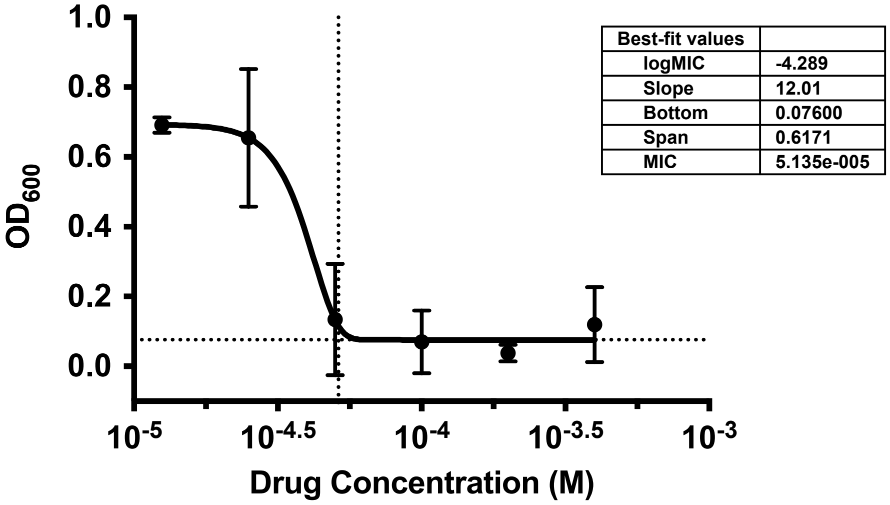

Compound 11 with *S. aureus*

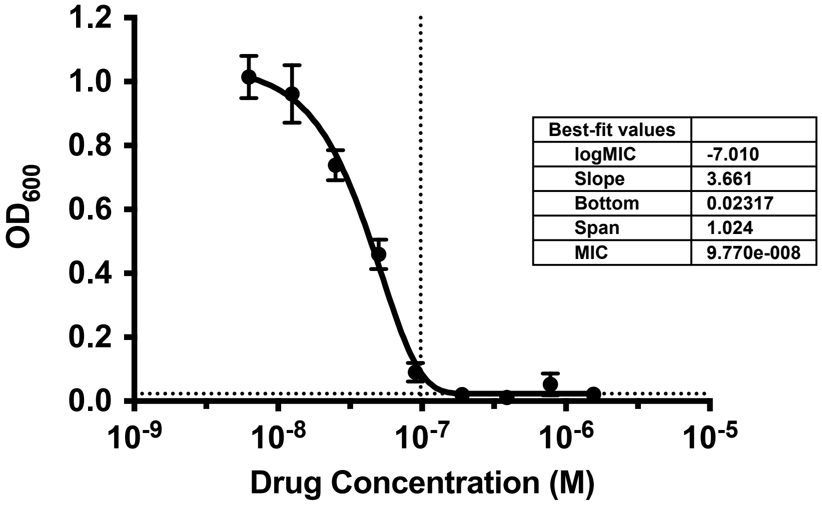

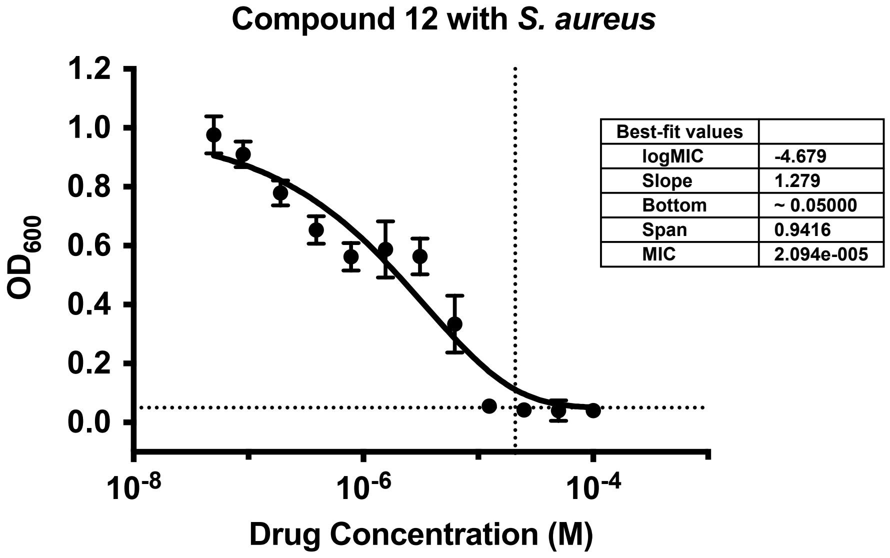

S3.2. *E. coli* MIC curves

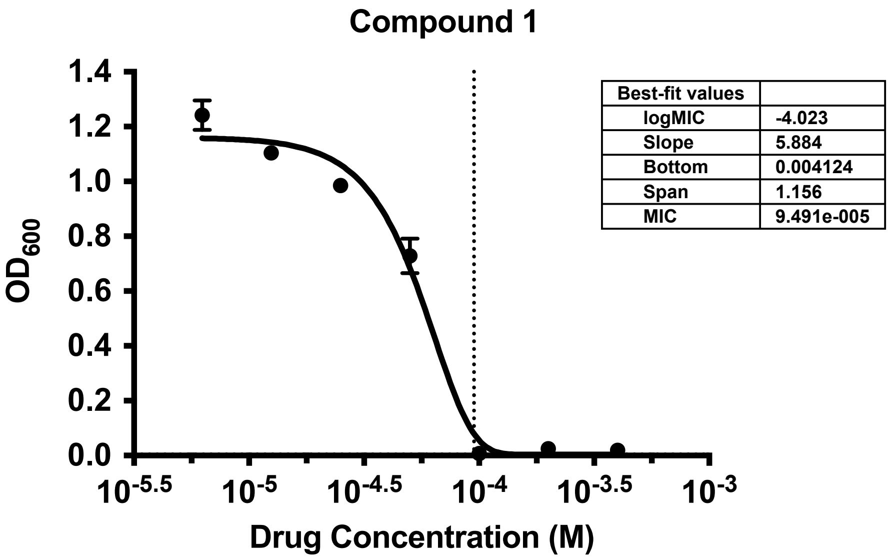

Compound 2 with *E. coli*

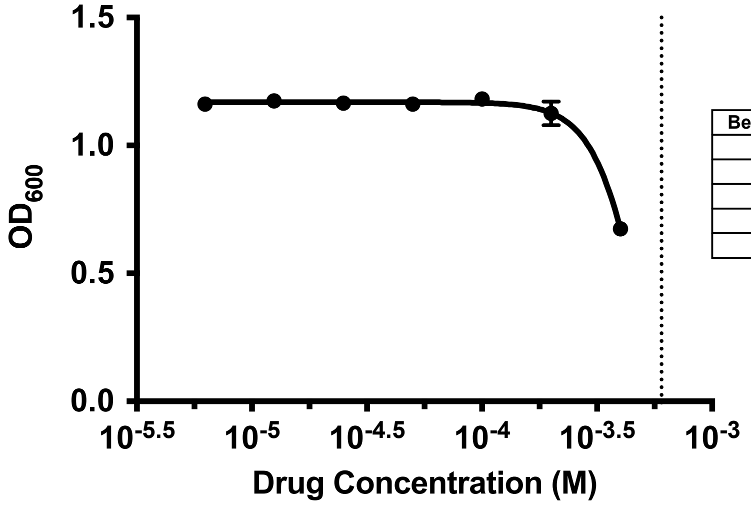

Best-fit values	
logMIC	-3.220
Slope	8.981
Bottom	= 0.000
Span	1.169
MIC	0.0006025

Compound 3 with *E. coli*

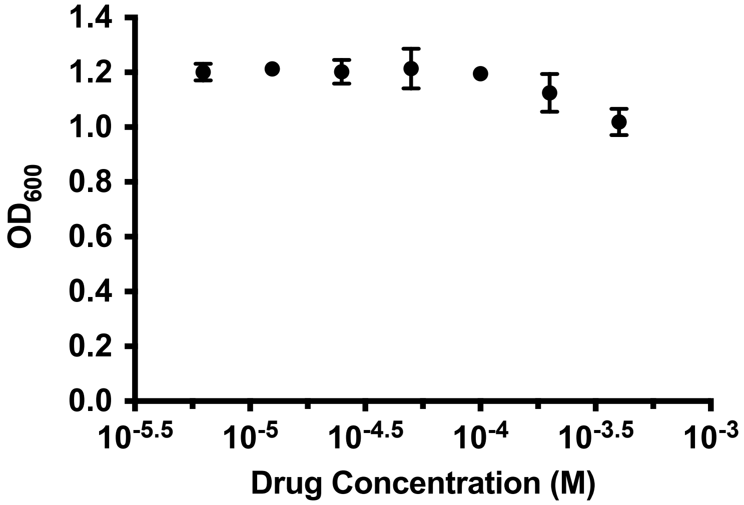

Compound 4 with *E. coli*

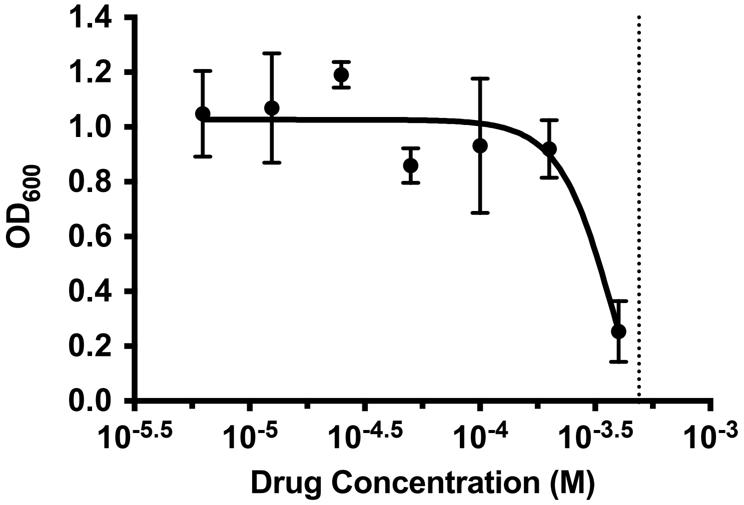

Best-fit values	
logMIC	-3.310
Slope	7.713
Bottom	~ 8.465e-014
Span	~ 1.027
MIC	0.0004898

Compound 5 with *E. coli*

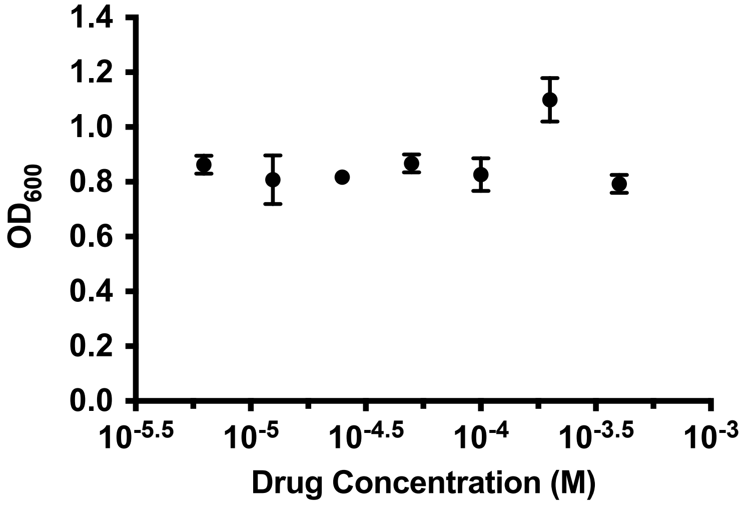

Compound 6 with *E. coli*

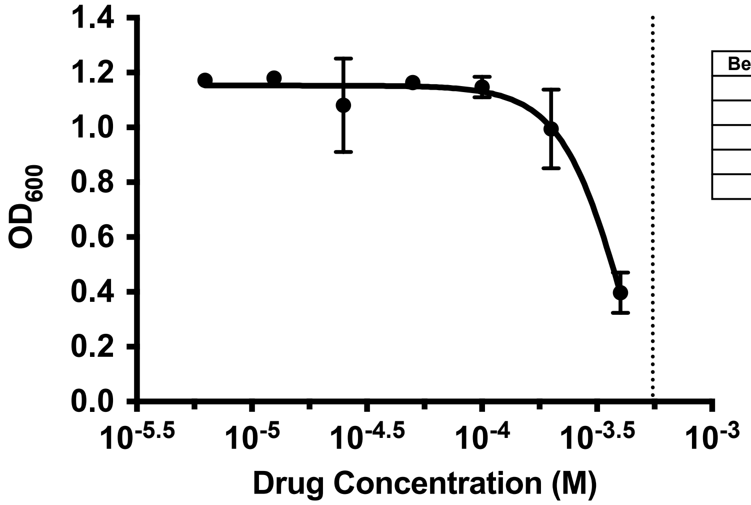

Best-fit values	
logMIC	-3.258
Slope	6.682
Bottom	= 0.000
Span	1.153
MIC	0.0005519

Compound 7 with *E. coli*

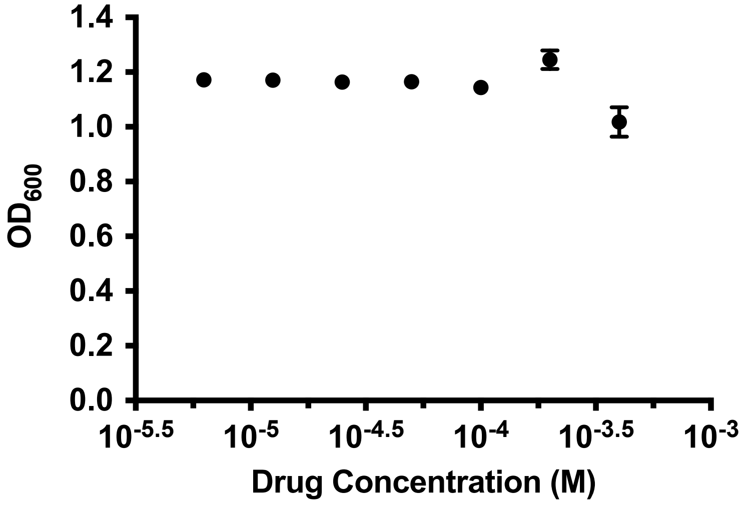

Compound 8 with *E. coli*

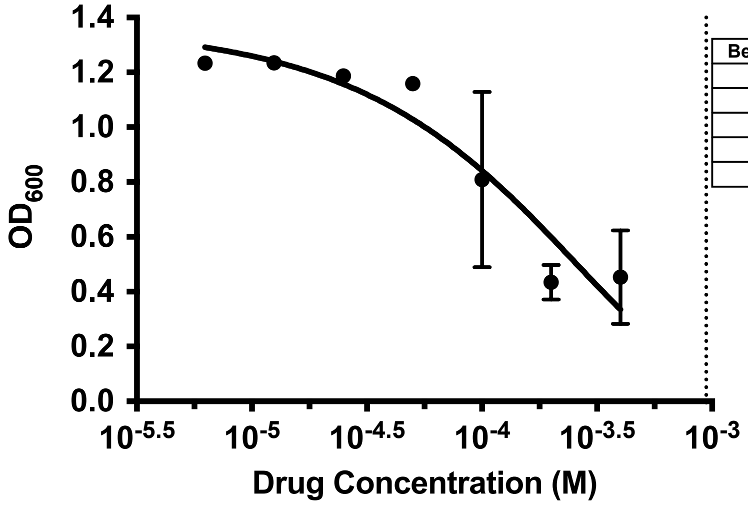

Best-fit values	
logMIC	-3.026
Slope	1.769
Bottom	= 0.000
Span	1.368
MIC	0.0009423

Compound 9 with *E. coli*

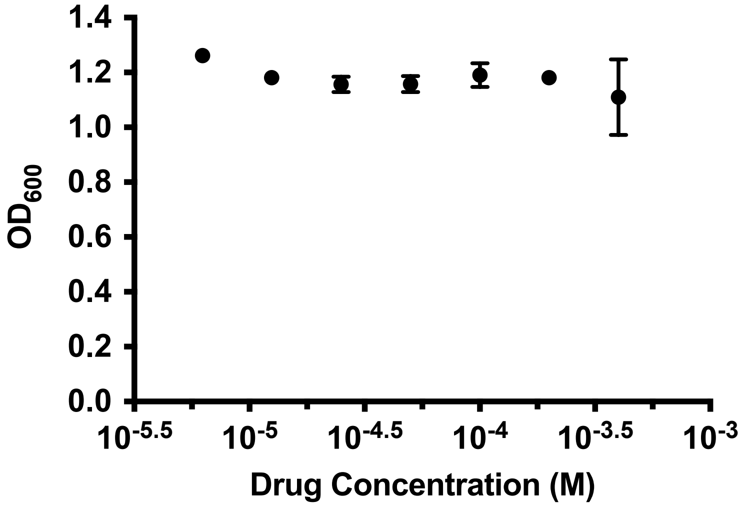

Compound 10 with *E. coli*

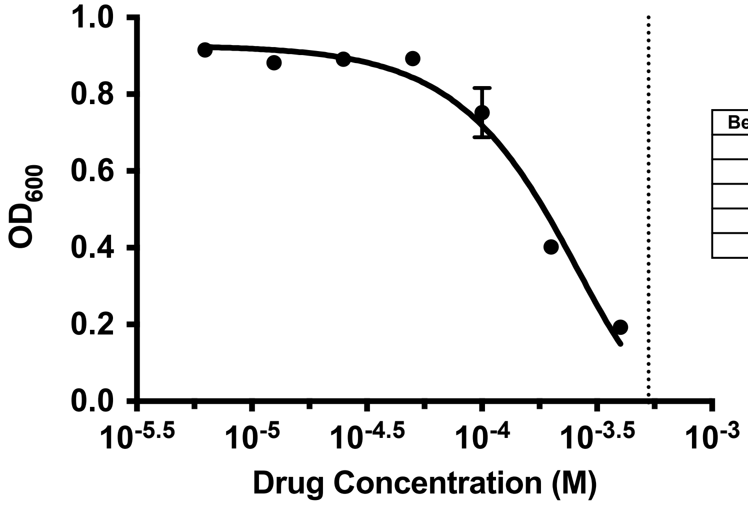

Best-fit values	
logMIC	-3.276
Slope	3.272
Bottom	= 0.000
Span	0.9275
MIC	0.0005292

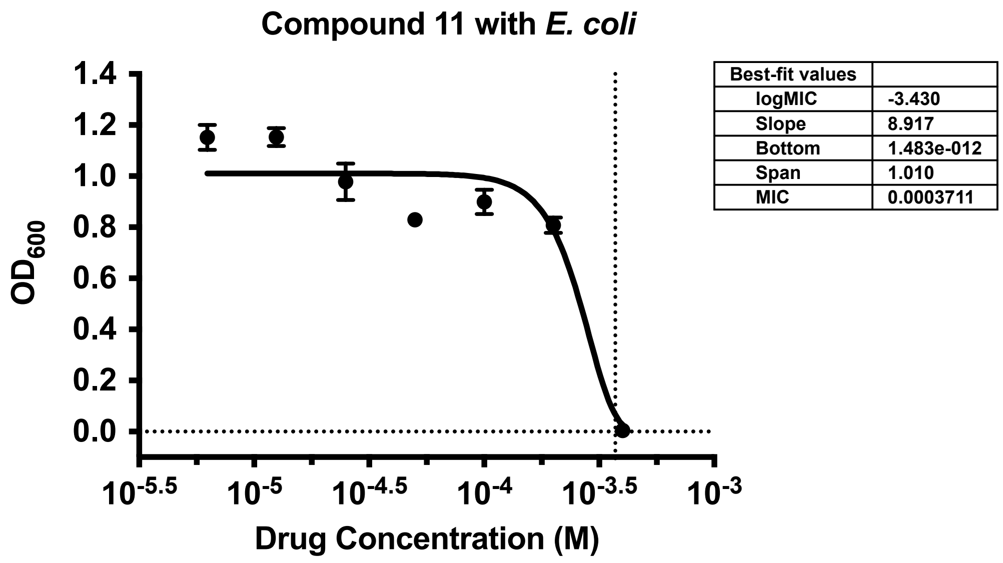
Compound 11 with *E. coli*
OD600
1.4
1.2
1.0
0.8
0.6
0.4
0.2
0.0
10-5.5
10-5
10-4.5
10-4
10-3.5
10-3
Drug Concentration (M)
Best-fit values
logMIC -3.430
Slope 8.917
Bottom 1.483e-012
Span 1.010
MIC 0.0003711

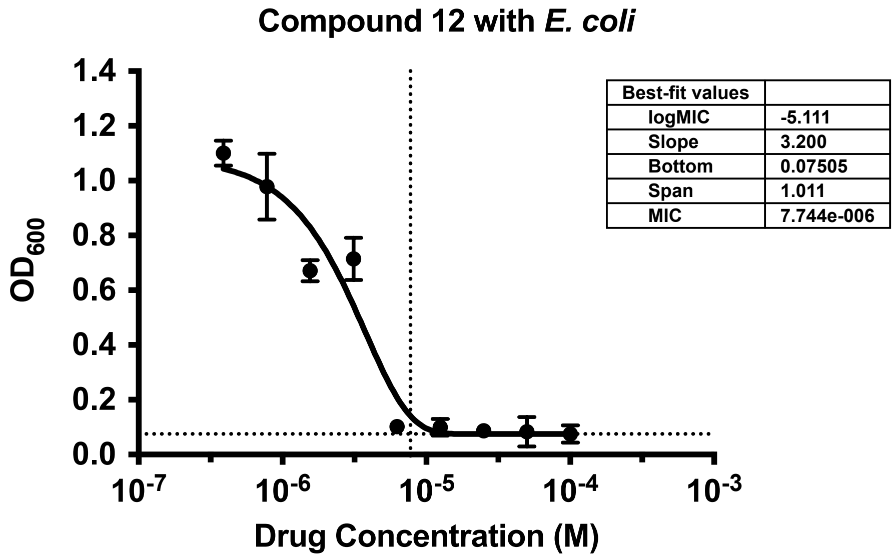
Compound 12 with *E. coli*
OD600
1.4
1.2
1.0
0.8
0.6
0.4
0.2
0.0
10-7
10-6
10-5
10-4
10-3
Drug Concentration (M)
Best-fit values
logMIC -5.111
Slope 3.200
Bottom 0.07505
Span 1.011
MIC 7.744e-006

S3.3. MBC_{50} data

Protocol: After 16 h of incubation with compounds at 37 °C as per the MIC assay protocol, a 1 μL aliquot was taken from wells with no visible bacterial growth and transferred to the surface of the agar plate. Plates were incubated for a further 24 h at 37 °C. MBC_{50} values were the lowest concentration of compound required to kill 50%, plates with no antibiotics were used as references for 100% growth. Experiments were performed in triplicate.

Compound	MBC_{50} *S. aureus* (NCTC 6571)	MBC_{50} *E. coli* (BW25113)
**1**	400 μM	>400 μM[a]
**2**	200 μM	>400 μM[a]
**3**	200 μM	>400 μM[a]
**4**	50 μM	>400 μM[a]
**5**	50 μM	>400 μM[a]
**6**	200 μM	>400 μM[a]
**7**	200 μM	>400 μM[a]
**8**	200 μM	>400 μM[a]
**9**	50 μM	>400 μM[a]
**10**	50 μM	>400 μM[a]
**11**	10 μM	400 μM
**12**	100 μM	100 μM

[a] MBC_{50} not determined in concentration range tested (up to 400 μM).

S4. UV Kinetics Assay

S4.1. Compound 1

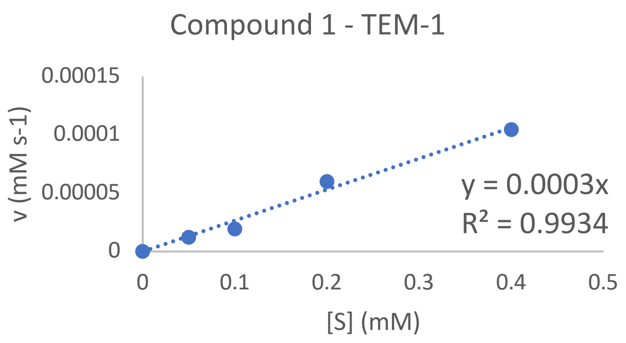

Compound 1 – TEM-1	
[E]	500 nM
Kcat/K_M (mM^{-1} s^{-1})	0.530
Relative k_{cat}/K_M	1.0

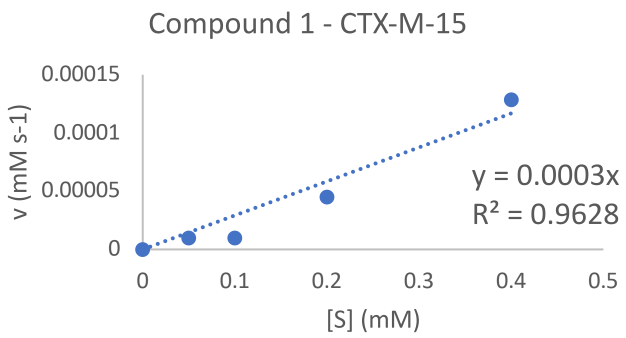

Compound 1 – CTX-M-15	
[E]	100 nM
Kcat/K_M (mM^{-1} s^{-1})	2.922
Relative k_{cat}/K_M	1.0

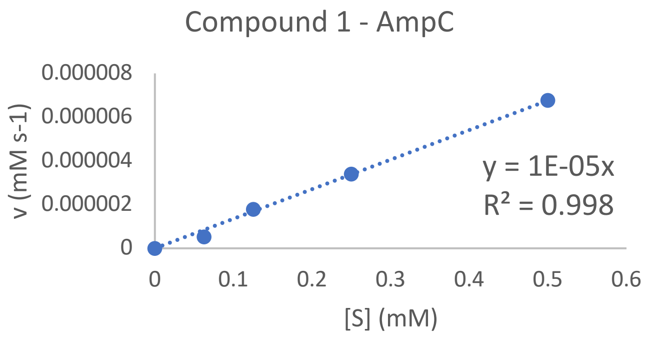

Compound 1 – AmpC	
[E]	50 nM
Kcat/K_M (mM^{-1} s^{-1})	0.270
Relative k_{cat}/K_M	1.0

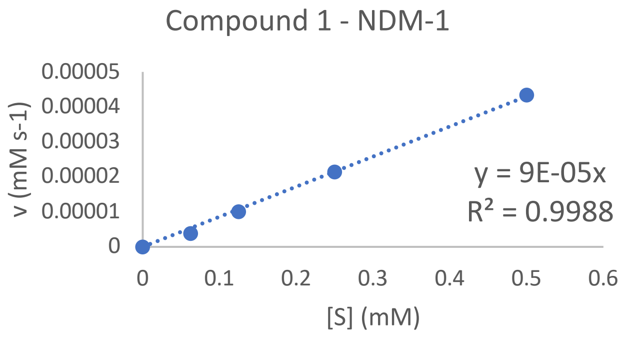

Compound 1 – NDM-1	
[E]	50 nM
Kcat/K_M (mM^{-1} s^{-1})	1.721
Relative k_{cat}/K_M	1.0

S4.2. Compound 2

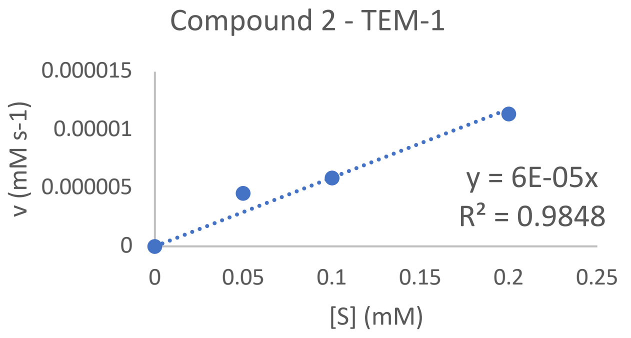

Compound 2 – TEM-1	
**[E]**	500 nM
**Kcat/K_M (mM^{-1} s^{-1})**	0.118
**Relative k_{cat}/K_M**	0.2

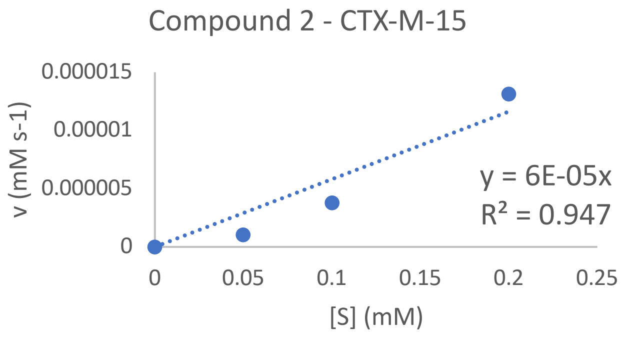

Compound 2 – CTX-M-15	
**[E]**	100 nM
**Kcat/K_M (mM^{-1} s^{-1})**	0.581
**Relative k_{cat}/K_M**	0.2

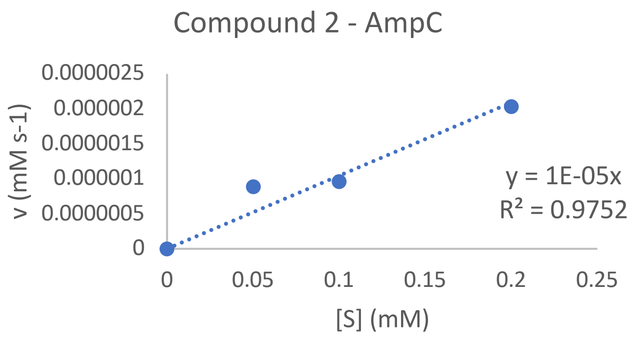

Compound 2 – AmpC	
**[E]**	50 nM
**Kcat/K_M (mM^{-1} s^{-1})**	0.209
**Relative k_{cat}/K_M**	0.7

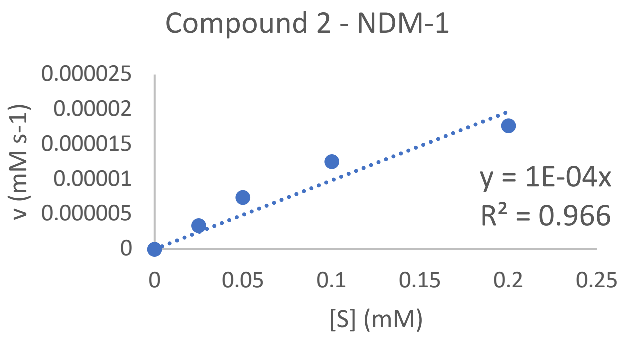

Compound 2 – NDM-1	
**[E]**	50 nM
**Kcat/K_M (mM^{-1} s^{-1})**	1.969
**Relative k_{cat}/K_M**	1.1

S4.3. Compound 3

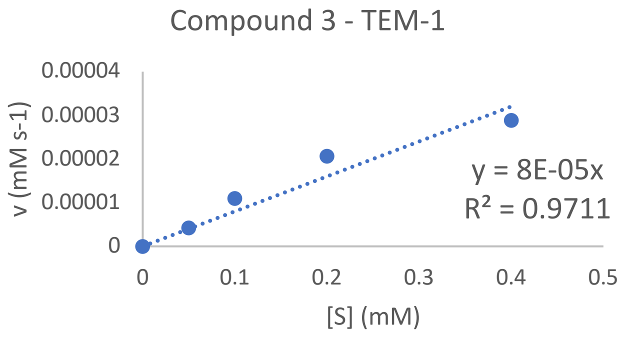

Compound 3 – TEM-1	
**[E]**	100 nM
**Kcat/K_M (mM^{-1} s^{-1})**	0.800
**Relative k_{cat}/K_M**	1.5

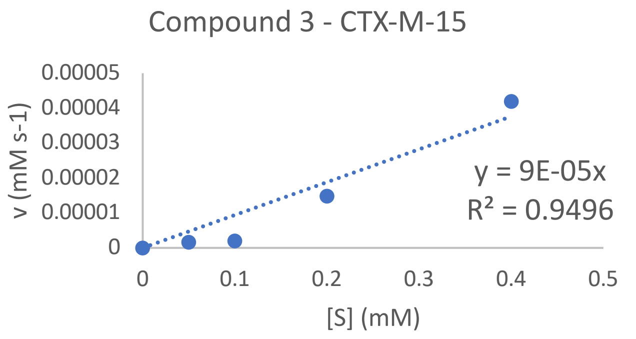

Compound 3 – CTX-M-15	
**[E]**	100 nM
**Kcat/K_M (mM^{-1} s^{-1})**	0.941
**Relative k_{cat}/K_M**	0.3

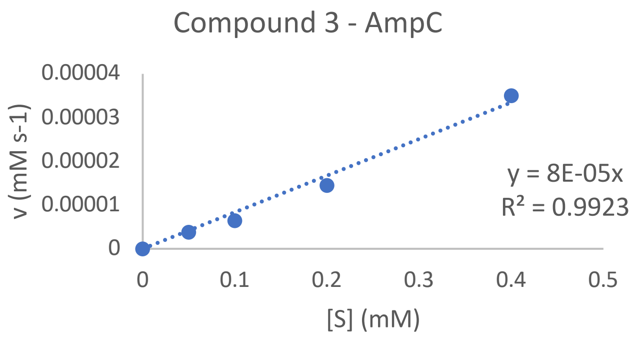

Compound 3 – AmpC	
**[E]**	50 nM
**Kcat/K_M (mM^{-1} s^{-1})**	1.673
**Relative k_{cat}/K_M**	5.7

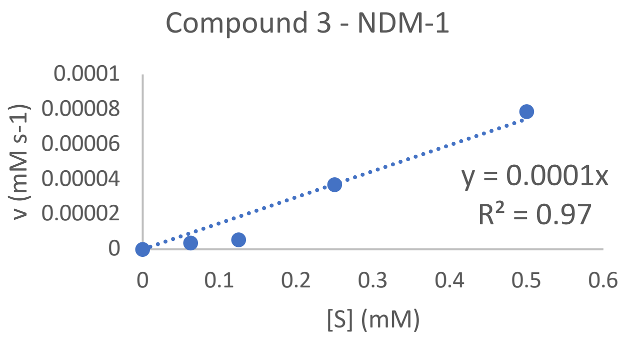

Compound 3 – NDM-1	
**[E]**	50 nM
**Kcat/K_M (mM^{-1} s^{-1})**	2.981
**Relative k_{cat}/K_M**	1.7

S4.4. Compound 4

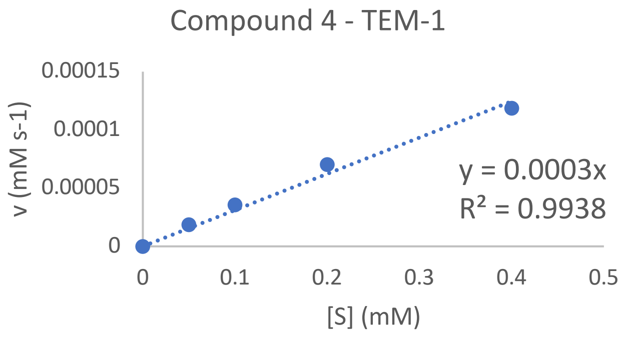

Compound 4 – TEM-1	
**[E]**	500 nM
**Kcat/K_M (mM^{-1} s^{-1})**	0.623
**Relative k_{cat}/K_M**	1.2

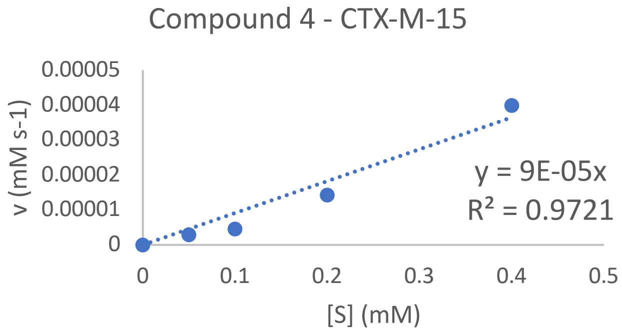

Compound 4 – CTX-M-15	
**[E]**	100 nM
**Kcat/K_M (mM^{-1} s^{-1})**	0.912
**Relative k_{cat}/K_M**	0.3

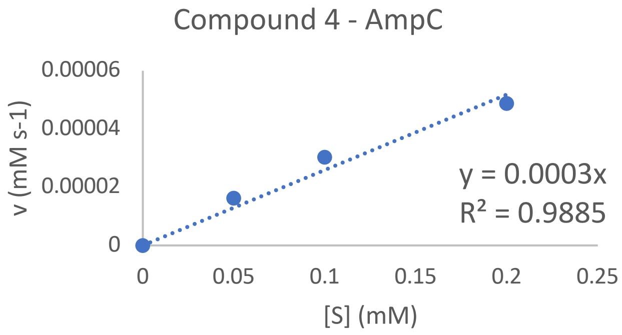

Compound 4 – AmpC	
**[E]**	50 nM
**Kcat/K_M (mM^{-1} s^{-1})**	5.182
**Relative k_{cat}/K_M**	17.3

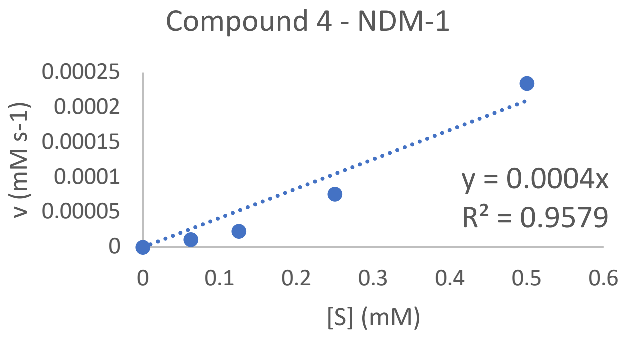

Compound 4 – NDM-1	
**[E]**	50 nM
**Kcat/K_M (mM^{-1} s^{-1})**	8.391
**Relative k_{cat}/K_M**	4.9

S4.5. Compound 5

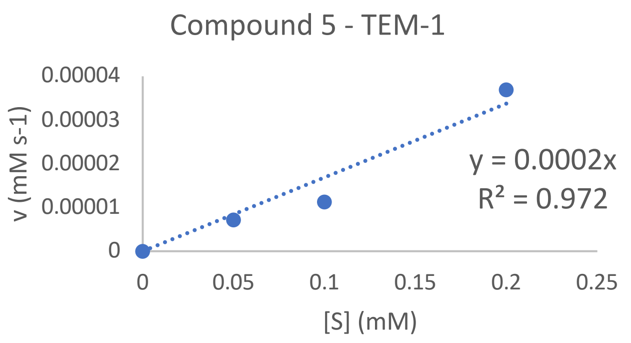

Compound 5 – TEM-1	
**[E]**	500 nM
**Kcat/K_M (mM^{-1} s^{-1})**	0.338
**Relative k_{cat}/K_M**	0.6

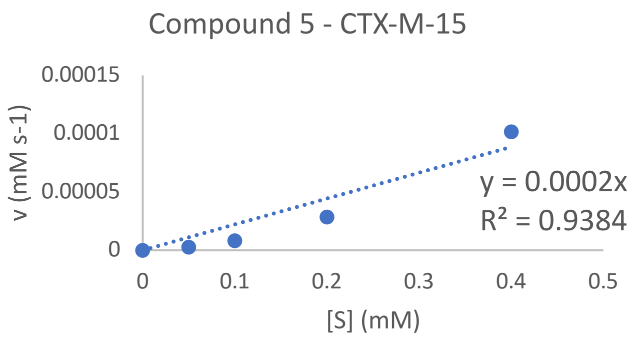

Compound 5 – CTX-M-15	
**[E]**	100 nM
**Kcat/K_M (mM^{-1} s^{-1})**	2.216
**Relative k_{cat}/K_M**	0.8

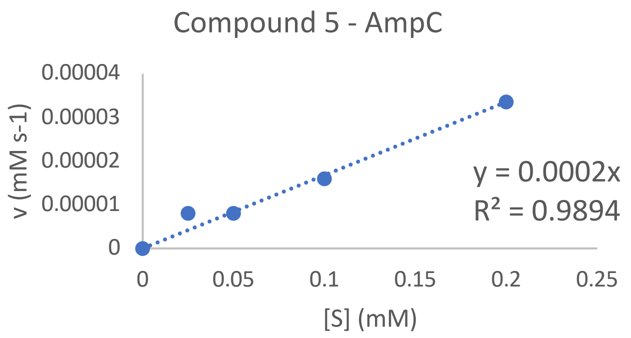

Compound 5 – AmpC	
**[E]**	50 nM
**Kcat/K_M (mM^{-1} s^{-1})**	3.355
**Relative k_{cat}/K_M**	11.3

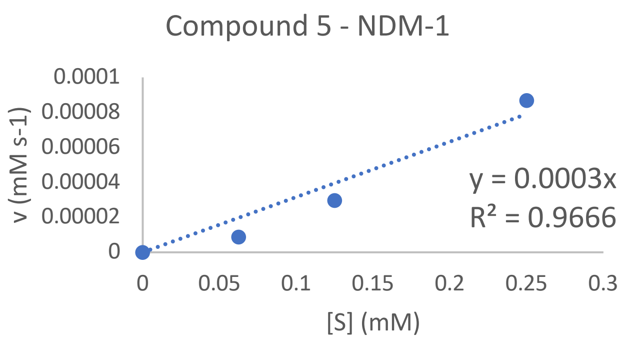

Compound 5 – NDM-1	
**[E]**	50 nM
**Kcat/K_M (mM^{-1} s^{-1})**	6.323
**Relative k_{cat}/K_M**	3.7

S4.6. Compound 6

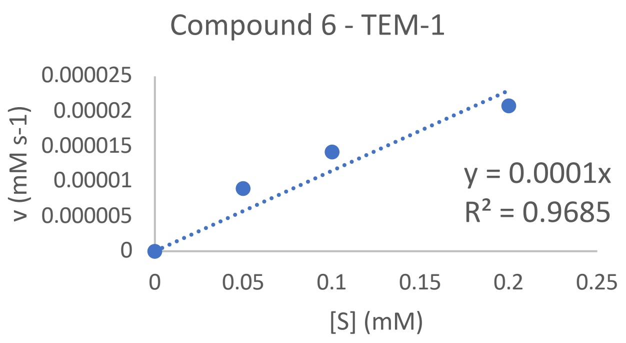

Compound 6 – TEM-1	
[E]	500 nM
Kcat/K_M (mM^{-1} s^{-1})	0.230
Relative k_{cat}/K_M	0.4

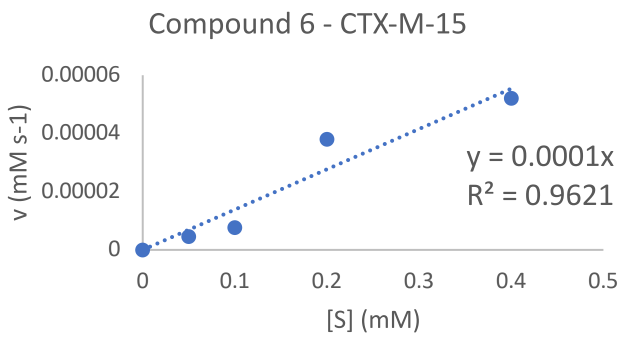

Compound 6 – CTX-M-15	
[E]	100 nM
Kcat/K_M (mM^{-1} s^{-1})	1.385
Relative k_{cat}/K_M	0.5

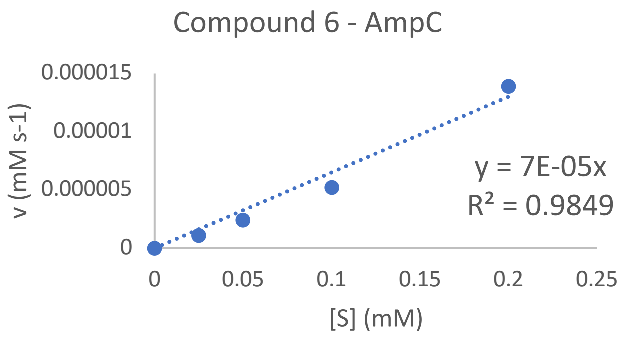

Compound 6 – AmpC	
[E]	50 nM
Kcat/K_M (mM^{-1} s^{-1})	1.301
Relative k_{cat}/K_M	4.3

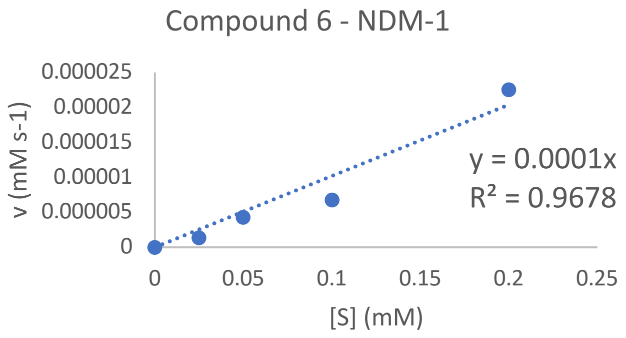

Compound 6 – NDM-1	
[E]	50 nM
Kcat/K_M (mM^{-1} s^{-1})	2.038
Relative k_{cat}/K_M	1.2

S4.7. Compound 7

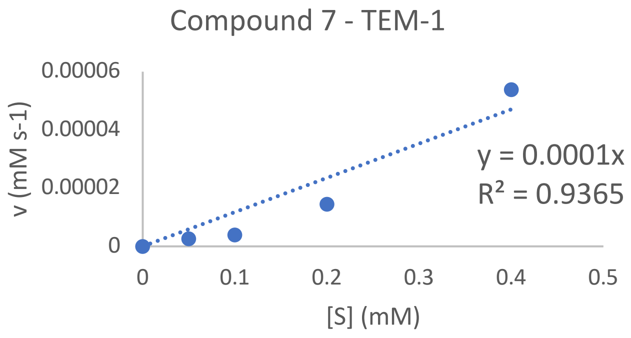

Compound 7 – TEM-1	
**[E]**	100 nM
**Kcat/K_M (mM^{-1} s^{-1})**	1.176
**Relative k_{cat}/K_M**	2.2

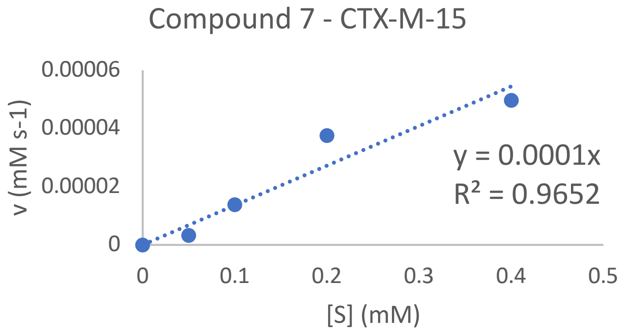

Compound 7 – CTX-M-15	
**[E]**	100 nM
**Kcat/K_M (mM^{-1} s^{-1})**	1.361
**Relative k_{cat}/K_M**	0.5

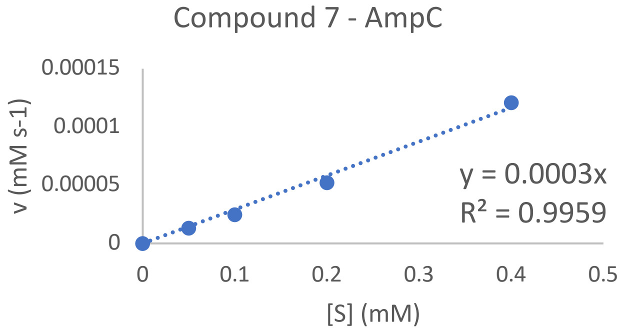

Compound 7 – AmpC	
**[E]**	50 nM
**Kcat/K_M (mM^{-1} s^{-1})**	5.830
**Relative k_{cat}/K_M**	19.3

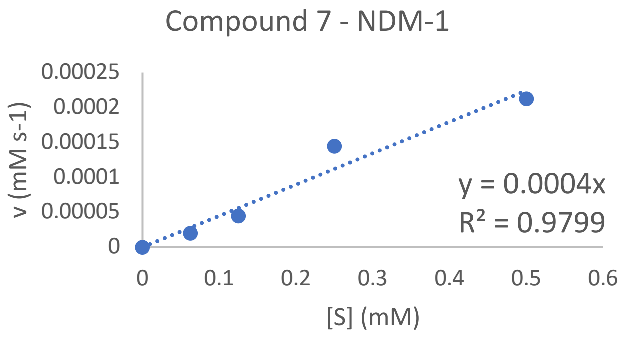

Compound 7 – NDM-1	
**[E]**	50 nM
**Kcat/K_M (mM^{-1} s^{-1})**	8.969
**Relative k_{cat}/K_M**	5.2

S4.8. Compound 8

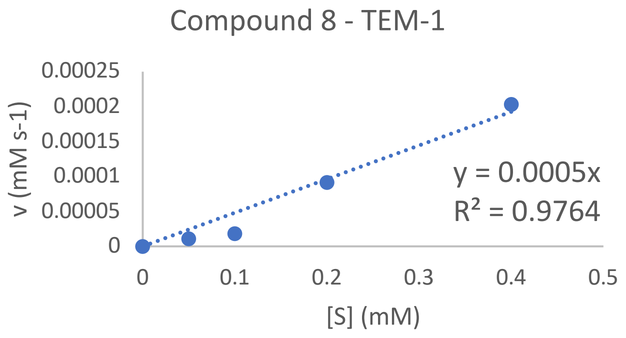

Compound 8 – TEM-1	
[E]	500 nM
Kcat/K_M (mM^{-1} s^{-1})	0.963
Relative k_{cat}/K_M	1.8

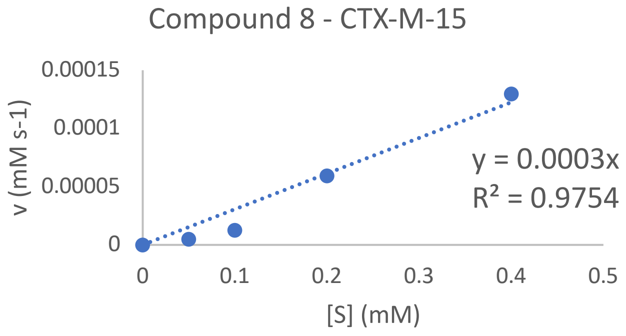

Compound 8 – CTX-M-15	
[E]	100 nM
Kcat/K_M (mM^{-1} s^{-1})	3.061
Relative k_{cat}/K_M	1.0

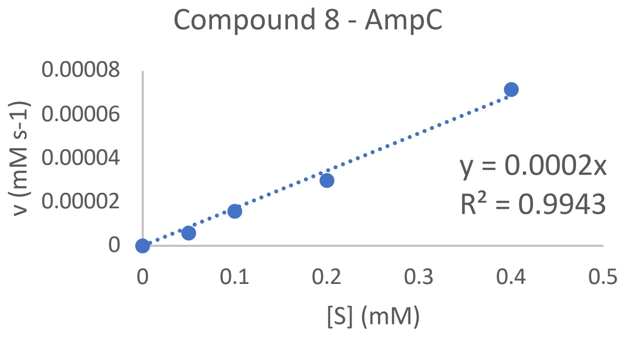

Compound 8 – AmpC	
[E]	50 nM
Kcat/K_M (mM^{-1} s^{-1})	3.432
Relative k_{cat}/K_M	11.3

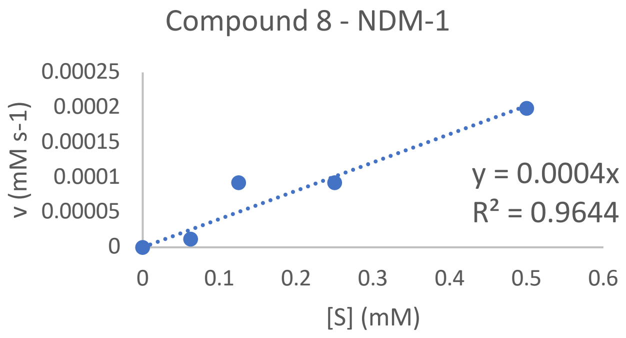

Compound 8 – NDM-1	
[E]	50 nM
Kcat/K_M (mM^{-1} s^{-1})	8.092
Relative k_{cat}/K_M	4.7

S4.9. Compound 9

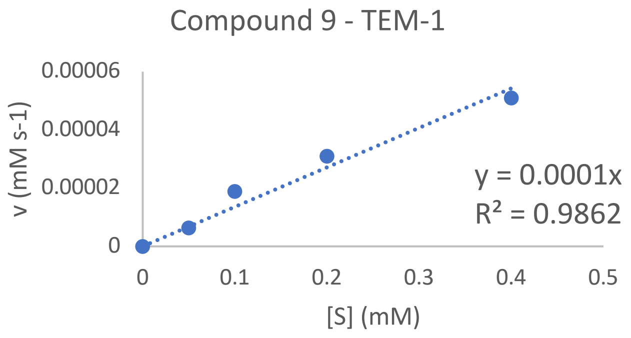

Compound 9 – TEM-1	
**[E]**	100 nM
**Kcat/K$_M$ (mM^{-1} s^{-1})**	1.357
**Relative k$_{cat}$/K$_M$**	2.6

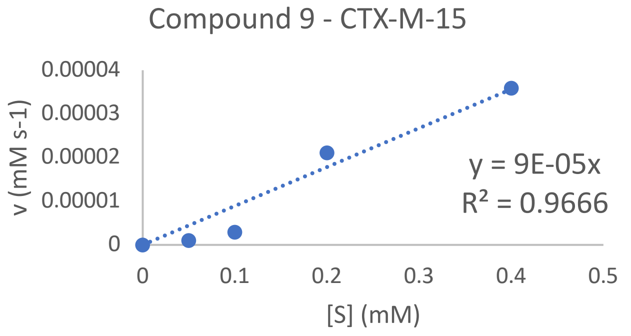

Compound 9 – CTX-M-15	
**[E]**	100 nM
**Kcat/K$_M$ (mM^{-1} s^{-1})**	0.891
**Relative k$_{cat}$/K$_M$**	0.3

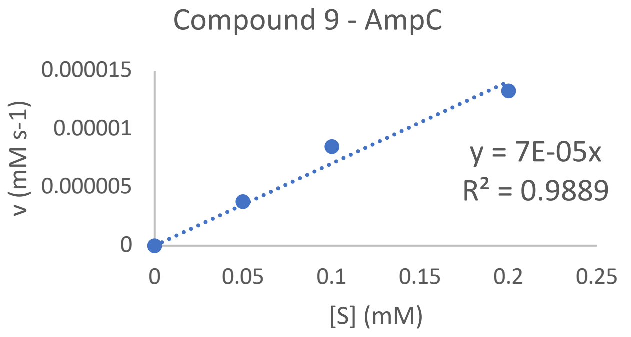

Compound 9 – AmpC	
**[E]**	50 nM
**Kcat/K$_M$ (mM^{-1} s^{-1})**	1.411
**Relative k$_{cat}$/K$_M$**	4.7

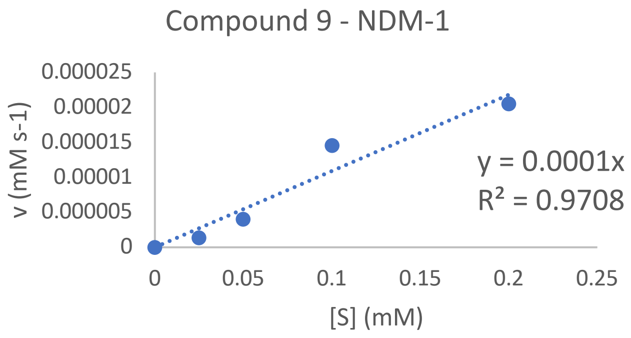

Compound 9 – NDM-1	
**[E]**	50 nM
**Kcat/K$_M$ (mM^{-1} s^{-1})**	2.181
**Relative k$_{cat}$/K$_M$**	1.3

S4.10. Compound 10

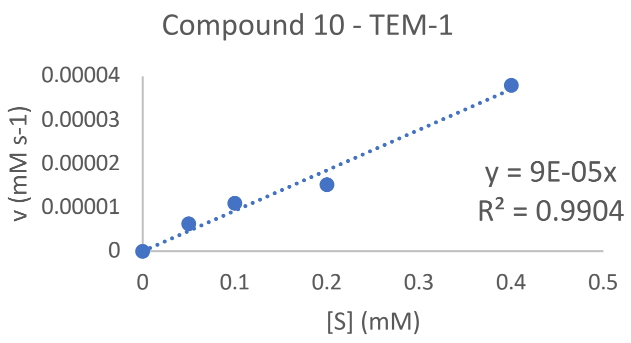

Compound 10 – TEM-1	
**[E]**	500 nM
**Kcat/K_M (mM^{-1} s^{-1})**	0.185
**Relative k_{cat}/K_M**	0.3

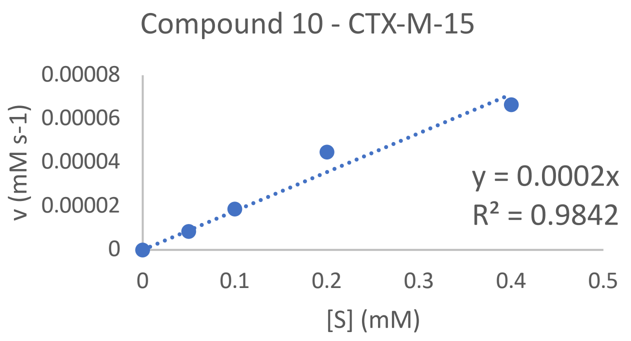

Compound 10 – CTX-M-15	
**[E]**	100 nM
**Kcat/K_M (mM^{-1} s^{-1})**	1.783
**Relative k_{cat}/K_M**	0.6

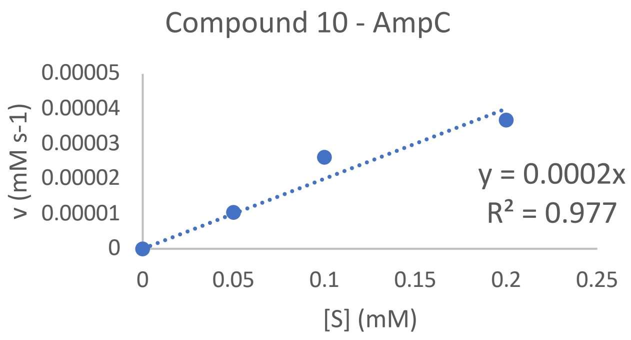

Compound 10 – AmpC	
**[E]**	50 nM
**Kcat/K_M (mM^{-1} s^{-1})**	4.007
**Relative k_{cat}/K_M**	13.3

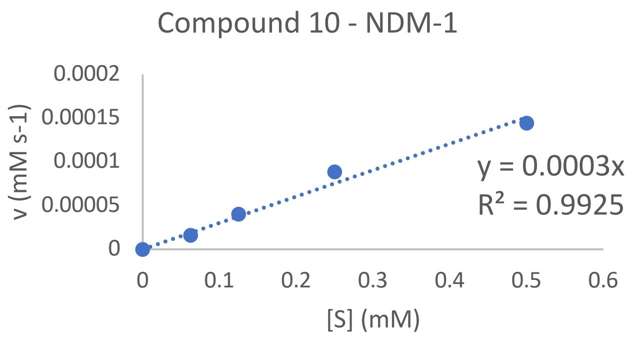

Compound 10 – NDM-1	
**[E]**	50 nM
**Kcat/K_M (mM^{-1} s^{-1})**	6.031
**Relative k_{cat}/K_M**	3.5
